# Synthesis and Antifouling Activity Evaluation of Analogs of Bromosphaerol, a Brominated Diterpene Isolated from the Red Alga *Sphaerococcus coronopifolius*

**DOI:** 10.3390/md20010007

**Published:** 2021-12-22

**Authors:** Kyriakos C. Prousis, Stefanos Kikionis, Efstathia Ioannou, Silvia Morgana, Marco Faimali, Veronica Piazza, Theodora Calogeropoulou, Vassilios Roussis

**Affiliations:** 1 Institute of Chemical Biology, National Hellenic Research Foundation, 48 Vassileos Constantinou Avenue, 11653 Athens, Greece; kyrprous@eie.gr; 2 Section of Pharmacognosy and Chemistry of Natural Products, Department of Pharmacy, National and Kapodistrian University of Athens, Panepistimiopolis Zografou, 15771 Athens, Greece; skikionis@pharm.uoa.gr (S.K.); eioannou@pharm.uoa.gr (E.I.); 3 Institute for the Study of Anthropic Impacts and Sustainability in Marine Environment (IAS), National Research Council (CNR), Via De Marini 6, 16149 Genova, Italy; silvia.morgana@ias.cnr.it (S.M.); marco.faimali@ias.cnr.it (M.F.)

**Keywords:** bromosphaerol, *Sphaerococcus coronopifolius*, synthetic analogs, antifouling activity, *Amphibalanus amphitrite*

## Abstract

Marine biofouling is an epibiotic biological process that affects almost any kind of submerged surface, causing globally significant economic problems mainly for the shipping industry and aquaculture companies, and its prevention so far has been associated with adverse environmental effects for non-target organisms. Previously, we have identified bromosphaerol (**1**), a brominated diterpene isolated from the red alga *Sphaerococcus coronopifolius*, as a promising agent with significant antifouling activity, exerting strong anti-settlement activity against larvae of *Amphibalanus* (*Balanus*) *amphitrite* and very low toxicity. The significant antifouling activity and low toxicity of bromosphaerol (**1**) motivated us to explore its chemistry, aiming to optimize its antifouling potential through the preparation of a number of analogs. Following different synthetic routes, we successfully synthesized 15 structural analogs (**2**–**16**) of bromosphaerol (**1**), decorated with different functional groups. The anti-settlement activity (EC_50_) and the degree of toxicity (LC_50_) of the bromosphaerol derivatives were evaluated using cyprids and nauplii of the cirriped crustacean *A. amphitrite* as a model organism. Derivatives **2**, **4**, and **6**–**16** showed diverse levels of antifouling activity. Among them, compounds **9** and **13** can be considered as well-performing antifoulants, exerting their activity through a non-toxic mechanism.

## 1. Introduction

Marine biofouling is an epibiotic biological process that is characterized by the attachment of various micro- and macro-organisms of the marine environment on submerged surfaces [1,2,3,4,5]. It is a build-up process in which the initial conditioning film formation occurring over swamped surfaces is followed by a biofilm adhesion caused by bacterial and algal cell colonization leading to the settlement of macrofouling organisms [6,7,8,9].

Marine biofouling represents a global phenomenon generating undoubtedly profound economic and ecological problems that needs to be addressed vigorously. It affects almost any kind of submerged surface, including aquaculture systems, coastal electric power stations, various underwater constructions, and marine vessels [10,11,12]. It has been and remains a major issue for the shipping industry with an outstanding financial cost, mandating huge capital investments for its control and efficient management, often associated until recently with detrimental environmental effects. The attachment of microbial slimes, algae, and marine sessile organisms, such as barnacles and mussels, on the rough surfaces of ship hulls results in increased weight and hydrodynamic frictional resistance, accounting for a tremendous increase in fuel consumption, greenhouse gas emissions, dry-docking time, vessel maintenance, and marine transport cost [13,14,15,16,17,18].

Several antifouling methods have been developed over the years, with the most effective being based mainly on biocidal chemicals incorporated in coatings and paints. The antifouling organotin-based paints that have been widely applied, such as tributyltin (TBT), have been proven very effective in preventing fouling [19,20,21,22]. However, they are characterized by severe toxicity for marine life and therefore are nowadays prohibited worldwide by the International Maritime Organization (IMO) [23]. Their replacement by copper-based coatings and booster biocides, such as diuron, Irgarol 1051, SeaNine 211, and zinc pyrithione, failed to adequately address the issue of environmental impact [24], and most of them have already been banned by many European countries [25]. Inert, silicon-based and polymer-based coatings that were subsequently applied as an alternative approach have been proven inefficient since they are expensive, difficult to apply, and not durable enough to provide long-lasting hull protection, while the environmental impact of their additives still remains undetermined [26].

Concerning marine ecosystem and human health, an ecological approach is unquestionably the only way forward for antifouling technology [27,28]. The ideal candidate for biofouling control should be environmentally safe, providing maximum fouling protection. The solution for alternative, effective, non-toxic antifouling agents could be lying in the chemistry of marine natural products [29,30,31,32,33]. In the very competitive marine ecosystem, organisms, such as seaweeds, sponges, corals, and other invertebrates, have developed various chemical and biological defense mechanisms to protect their surfaces from fouling. Secondary metabolites (e.g., sulfated polyphenols, steroids, terpenoids, and alkaloids) and biopolymers have been reported to be involved in different interactions of marine life for the repellence, inhibition, and suppression of settlement and growth of fouling organisms [33]. Many isolated compounds from marine prokaryotes and eukaryotes have shown antifouling activity, fulfilling the U.S. Navy Program standards of EC_50_ values < 25.0 mg/L, with actual EC_50_ values less than 5 mg/L. Among them, macroalgal secondary metabolites have been regarded as non-toxic antifouling agents with great potential [34,35].

In the framework of our research investigations, we have previously identified bromosphaerol (**1**), a brominated diterpene isolated from the red alga *Sphaerococcus coronopifolius*, as a promising agent with significant antifouling activity [36,37]. In these studies, bioassays conducted using larvae of the cirriped crustacean *Amphibalanus* (*Balanus*) *amphitrite* showed that bromosphaerol (**1**) exerted significant anti-settlement activity with an EC_50_ value of 0.23 mg/L, combined with extremely low toxicity (LC_50_ > 100 mg/L), resulting in an impressive therapeutic ratio (TR_C_ = LC_50_/EC_50_) of 434.78. The significant antifouling activity and low toxicity of bromosphaerol (**1**) motivated us to explore its chemistry, aiming to optimize its antifouling potential through the preparation of a number of analogs. Herein, we report the synthesis of a series of bromosphaerol derivatives, along with the evaluation of their antifouling activity.

## 2. Results and Discussion

In order to improve the antifouling potential of the brominated diterpene bromosphaerol (**1**), 15 structural analogs involving transformations at Δ^1^ double bond and positions C-11, C-16, and C-17 (**2**–**16**) were designed and synthesized (Figure 1 and Figures S1–S46). Our strategy for obtaining initial structure-antifouling activity relationships for bromosphaerol involved (a) introducing polar groups at C-1 and/or C-2 (**2**–**6** and **8**), (b) removing the C-11 hydroxyl group **(9** and **10**), and (c) substituting C-2 with functional groups (ester and oxime), while the Δ^1^ double bond was repositioned to C-1–C-10 to allow for the generation of an extended conjugated system (**7** and **11**–**16**).

**Figure 2:**
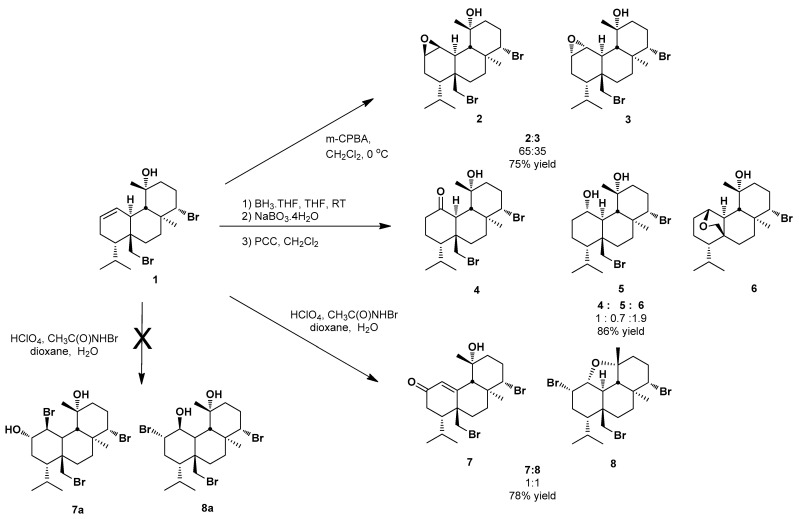
Synthesis of bromosphaerol derivatives **2**–**8**.

Initially, our synthetic efforts were focused on introducing an epoxide ring at the Δ^1^ double bond of **1**. This was achieved using *p*-chloroperbenzoic acid in dichloromethane, which resulted in a mixture of the two diastereomeric epoxides **2** and **3** (Figure 2) in a 65:35 ratio and 75% overall yield, both of which were subsequently isolated in pure form. The configuration at the chiral centers C-1 and C-2 of **2** was deduced as 1*S*,2*R* on the basis of NOE enhancements of H-1 and H-2 with H_3_-19 and H_3_-20, respectively.

Subsequently, we aimed at introducing a carbonyl functionality at C-1 or C-2. Therefore, bromosphaerol (**1**) was subjected to a hydroboration/oxidation sequence, using a borane tetrahydrofuran complex and sodium perborate, followed by pyridinium chlorochromate (PCC)-mediated oxidation of the generated alcohols. The above synthetic strategy resulted in a mixture of the ketone **4** and the precursor alcohol **5** due to incomplete oxidation, in a 1:0.7 ratio and a 41% yield. As a side product of the reaction, compound **6** was isolated in 45% yield (Figure 2). Compound **6**, bearing an oxygen bridge between C-1 and C-17, was formed during the hydroboration step through an SN_2_ nucleophilic attack of the C-1 epimer of **5** on the brominated carbon C-17 and could not be oxidized further upon PCC treatment.

In a next step, we attempted to synthesize the bromohydrin derivatives **7a** and **8a** of **1** through treatment with *N*-bromoacetamide in the presence of perchloric acid in a mixture of water/dioxane. Instead, we isolated the *α,β*-unsaturated ketone **7,** and the C-2 brominated compound **8** (Figure 2). Compound **7** could be generated from the intermediate bromohydrin **7a** through oxidation of the C-2 alcohol followed by E2 elimination of the C-1 bromine and subsequent formation of the C-1–C-10 double bond. Compound **7** was also isolated as the single product from the oxidation of the mixture of the diastereomeric epoxides **2** and **3** using Jones reagent. On the other hand, hypobromous acid generated in situ from NBA and perchloric acid reacted, with the Δ^1^ double bond affording the 2α-bromo C-1 carbocation intermediate, which reacted further with the C-11 alcohol to produce the corresponding compound **8** bearing a bromine atom at C-2 and an additional fused furan ring composed of C-1, C-10, C-9, and C-11. The stereochemistry at the chiral centers C-1, C-2, and C-11 was deduced as 1*R*,2*S*,11*R* on the basis of the cross-peaks observed in the NOESY spectrum, as well as the measured coupling constants. In particular, the coupling constant measured between H-1 and H-10 (*J* = 11.8 Hz) allowed for the determination of the configuration of the oxygenated H-1 as axial, whereas the coupling constant measured between H-1 and H-2 (*J* = 3.2 Hz) allowed for the determination of the configuration of the bromomethine H-2 as equatorial. Additionally, the NOE correlations of H-1 with H-9, H_3_-11, and H-17b, as well as of H_3_-11 with H-9 and H-14, dictated that the stereochemistry at C-11 remained as *R*. This proposed mechanism is supported by the relative *cis* stereochemistry of H-1 and H-2 in compound **8**. In the case of the formation of a bromonium ion intermediate, an *anti*-addition would be expected, resulting in a *trans* relative stereochemistry of H-1 and H-2.

**Figure 3:**
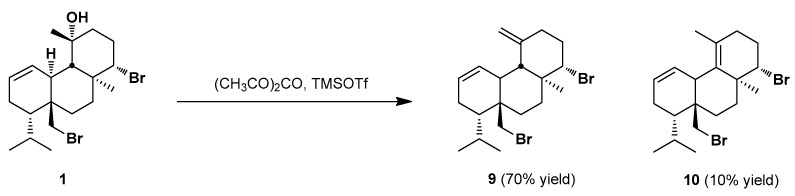
Synthesis of bromosphaerol derivatives **9** and **10**.

Furthermore, the regiomeric olefins **9** and **10** were formed in 70% and 10% yield, respectively, by an elimination reaction of the hydroxyl group at C-11 upon treatment of **1** with trimethylsilyl trifluoromethanesulfonate in the presence of acetic anhydride (Figure 3). Compounds **9** and **10**, named bromosphaerenes B and A, respectively, were previously isolated from *S. coronopifolius* by Fattorusso et al. (1983), and their structures were confirmed through dehydration of bromosphaerol (**1**) upon heating at 100 °C with phosphorus oxychloride–pyridine to afford bromosphaerene A (**10**) as the major product [38]. In contrast, our synthetic method provided preferentially bromosphaerene B (**9**).

**Figure 4:**
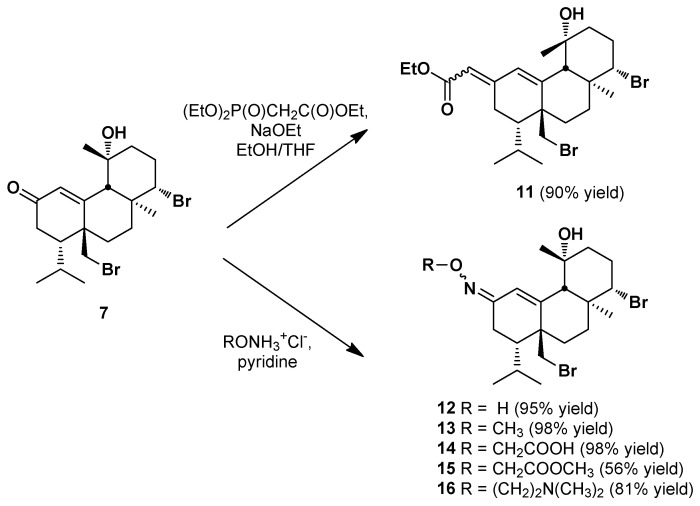
Synthesis of bromosphaerol derivatives **11**–**16**.

The *α,β*-unsaturated ketone **7** was proven to be a valuable synthetic intermediate toward the synthesis of a number of analogs. Thus, the Horner–Wadsworth–Emmons reaction of **7** with triethyl phosphonoacetate afforded the unsaturated ester **11** in 90% yield (Figure 4), as a mixture of *E*,*Z* geometric isomers (in a 6 to 4 ratio). Finally, a series of oxime derivatives (**12**–**16**) were obtained in good to excellent yields (56 to 98%), using ketone **7** and various substituted alkoxyamines in the presence of pyridine (Figure 4).

The results of the settlement inhibition assay performed on cypris larvae of *A. amphitrite* and the mortality test carried out on the stage II nauplii of the same model organism for bromosphaerol derivatives **2**, **4**, and **6**–**16** are shown in Table 1, summarizing the EC_50_ values obtained for cyprids settlement test (after 72 h), the LC_50_ values obtained for cypris larvae mortality (after 72 h), and the LC_50_ values of naupliar mortality (after 48 h). According to the guidelines of the U.S. Navy Program that require an EC_50_ (settlement inhibition) value lower than 25 mg/L for a compound to be considered as a promising natural antifoulant, 9 of the 13 derivatives tested in this study meet this requirement. In particular, compounds **2**, **4**, **6,** and **7,** which bear an oxygen moiety at C-1 or C-2, exhibit similar EC_50_ values ranging from 10.44 to 8.75 mg/L. Conversely, the introduction of a bromine substituent at C-2 abolishes activity. This is also the case for the *α*,*β*-unsaturated ester analog **11**. Interestingly, derivative **9,** in which the hydroxyl at C-11 was eliminated to form the exocyclic double bond, exhibited potent antifouling activity with an EC_50_ < 0.5 mg/L. Surprisingly, the endocyclic elimination congener **10** was less potent (EC_50_ = 3.87 mg/L). Furthermore, the activity of derivatives **12**–**16** was influenced significantly by the nature of the oxime functionality. Thus, the unsubstituted oxime compound **12** and the carboxy oxime derivative **14** were inactive, in contrast to the methoxy oxime analog **13** and the methyl ester congener of **14**, compound **15**, that possess EC_50_ < 0.5 mg/L. This is also the case for dimethylaminoethyl oxime derivative **16** showing EC_50_ < 0.5 mg/L. Thus, analogs **9**, **13**, **15**, and **16** exhibit very promising antifouling efficacy.

Concerning the toxicity observed on cypris larvae, the LC_50_ (72 h) values are >50 mg/L for derivatives **9** and **13**, while **15** and **16** demonstrated substantial toxicity towards this larval stage (LC_50_ values of 2.7 and 12.5 mg/L, respectively). This was also observed for the epoxy derivative **2** and the tetrahydrofuranyl analog **6,** possessing LC_50_ values of 25.2 and 10.2 mg/L, respectively. Gratifyingly, all the other compounds were not toxic against cypris larvae with LC_50_ values > 50 mg/L. However, all synthetic analogs displayed quite high toxicity towards the naupliar stage, with LC_50_ (48 h) values between 1.19 and 21.64 mg/L with the exception of the inactive **8** and **14** demonstrating LC_50_ values > 50 mg/L.

As previously reported [36], although the therapeutic ratio (TR) is traditionally calculated by taking into account naupliar mortality, measuring mortality on the same larval stage on which settlement is evaluated (cypris larvae, competent larval stage) is also important since TR should actually indicate whether the mechanism of settlement inhibition is based on a toxic effect. In addition, the LC_50_ on nauplii can be considered as a good index of toxicity against non-target organisms since nauplii are considered as a representative zooplankton organism. It is evident that naupliar response to the tested bromosphaerol derivatives is very different from that of cyprids; indeed, naupliar mortality occurs at lower concentrations than mortality of cyprids. Table 1 reports the TR values calculated taking into account both nauplii (TR_N_) and cyprids (TR_C_) mortality values. High TR values indicate a low toxicity anti-settlement mechanism for the tested compounds. For derivatives showing promising EC_50_ values (**9**, **13**, **15**, and **16**), the TR_N_ is quite low (ranging from 2.38 to 3.62), while higher values are observed for TR_C_, especially for derivatives **9** and **13** (>100). Concerning cyprids mortality, **9** and **13** showed LC_50(cypris)_ values higher than the maximum tested concentration (50 mg/L). Looking at the two more promising in terms of antifouling efficacy derivatives (**9** and **13**), the TR_N_ values are much lower than the TR_C_ values. Taking into account only the TR_N_ values, we can assert that analogs **9** and **13** are characterized by good antifouling properties (EC_50_ < 0.5 mg/L) but may exhibit some toxicity against non-target organisms, as mentioned above. Conversely, considering the TR_C_ values (>100) of **9** and **13**, both derivatives can be considered as well-performing antifoulants, exerting their activity through a non-toxic mechanism.

## 3. Materials and Methods

### 3.1. General Experimental Procedures

1D and 2D NMR spectra were recorded on Bruker DRX 400 (Bruker BioSpin GmbH, Rheinstetten, Germany) and Varian 300 and Varian 600 (Varian, Inc., Palo Alto, CA, USA) spectrometers, using standard Bruker or Varian pulse sequences. Chemical shifts internally referenced to residual solvent signals are given on a *δ* (ppm) scale. High-resolution ESI and APCI mass spectra were measured on a Thermo Scientific LTQ Orbitrap Velos mass spectrometer (ThermoFisher Scientific, Bremen, Germany). Column chromatography separations were performed with Kieselgel Si 60 (Merck, Darmstadt, Germany). HPLC separations were conducted on a CECIL 1100 Series liquid chromatography pump (Cecil Instruments Ltd., Cambridge, UK) equipped with a GBC LC-1240 refractive index detector (GBC Scientific Equipment, Braeside, VIC, Australia), using a 250 mm × 22 mm i.d. Techsil 10 ODS column (Wellington House, Cheshire, UK) for reversed-phase HPLC or a 250 mm × 10 mm i.d. Kromasil 100-10-SIL (Akzonobel, Eka Chemicals AB, Separation Products, Bohus, Sweden) for normal-phase HPLC. TLC was performed on Kieselgel 60 F_254_ (0.2 mm) precoated aluminum or glass plates (Merck, Darmstadt, Germany), and spots were visualized after spraying with H_2_SO_4_ in MeOH (20% *v*/*v*) reagent and heating at 100 °C for 1 min.

### 3.2. Biological Material

Specimens of *S. coronopifolius* were collected by scuba diving in the bay of Palaiokastritsa, Corfu, Greece, at a depth of 15–40 m in August 2013. A voucher specimen of the alga has been deposited at the Herbarium of the Section of Pharmacognosy and Chemistry of Natural Products, Department of Pharmacy, National and Kapodistrian University of Athens (ATPH/MP0226).

### 3.3. Extraction and Isolation

Air-dried algal tissues (0.5 kg dry weight) were exhaustively extracted with mixtures of CH_2_Cl_2_/MeOH (3:1) at room temperature. Evaporation of the solvents in vacuo afforded a dark green oily residue (18 g) that was subjected to vacuum column chromatography over silica gel, using cHex with increasing amounts of EtOAc and subsequently EtOAc with increasing amounts of MeOH as the mobile phase to yield 15 fractions (1–15). Fraction 3 (cHex/EtOAc 80:20, 10.9 g) was subjected to preparative reversed-phase HPLC using MeOH 100% as the mobile phase to afford sphaerococcenol A (2.1 g) and bromosphaerol (**1**) (4.45 g).

### 3.4. Synthesis of Analogs of Bromosphaerol

#### 3.4.1. Synthesis of Analogs **2** and **3**

To a stirred solution of bromosphaerol (**1**) (19.3 mg, 0.043 mmol) in anhydrous CH_2_Cl_2_ (0.8 mL), a solution of *m*-chloroperoxybenzoic acid 77% (14.45 mg, 0.065 mmol) in CH_2_Cl_2_ (0.8 mL) was added dropwise at 0 °C, and the reaction mixture was stirred for 2 h at 25 °C. Upon completion of the reaction (monitored by TLC), saturated aqueous Na_2_SO_3_ solution (2.0 mL) was added. The pH was adjusted to 7 by the addition of 5% aqueous NaHCO_3_ solution, and the reaction mixture was extracted with CH_2_Cl_2_ (3 × 15 mL). The combined organic layers were dried over anhydrous Na_2_SO_4_, concentrated under reduced pressure, dried in vacuo to afford a mixture of the diastereomeric epoxides **2** and **3** in a 65:35 ratio (15 mg, 75%), which was subjected to flash column chromatography (elution system: petroleum ether 40–65 °C/acetone, 40:60 to 95:10) and subsequently normal-phase HPLC using cHex/EtOAc (75:25) as eluent to afford **2** (9.5 mg) and **3** (3.8 mg) in pure form.

Compound **2**: Gummy solid; ^1^H NMR (600 MHz, CDCl_3_) *δ* 0.94 (3H, d, *J* = 6.9 Hz, H_3_-19), 0.97 (3H, d, *J* = 6.9 Hz, H_3_-20), 1.22 (1H, m, H-7b), 1.31 (3H, s, H_3_-15), 1.42 (3H, s, H_3_-16), 1.46 (1H, m, H-6b), 1.59 (1H, d, *J* = 10.8 Hz, H-9), 1.66 (1H, m, H-4), 1.68 (1H, m, H-12b), 1.73 (1H, m, H-6a), 1.74 (1H, m, H-12a), 1.75 (1H, m, H-7a), 1.87 (1H, ddd, *J* = 16.6, 6.4, 1.0 Hz, H-3b), 1.97 (1H, m, H-13β), 1.99 (1H, m, H-3a), 2.04 (1H, qd, *J* = 6.9, 1.6 Hz, H-18), 2.42 (1H, qd, *J* = 13.0, 4.3 Hz, H-13α), 2.72 (1H, dd, *J* = 10.8, 2.0 Hz, H-10), 3.26 (1H, m, H-2), 3.45 (1H, dd, *J* = 4.3, 2.0 Hz, H-1), 3.76 (1H, d, *J* = 10.9 Hz, H-17b), 3.98 (1H, dd, *J* = 13.0, 3.5 Hz, H-14), 4.21 (1H, dd, *J* = 10.9, 1.9 Hz, H-17a); ^13^C NMR (75 MHz, CDCl_3_) *δ* 14.0 (C-15), 19.9 (C-3), 20.8 (C-20), 24.9 (C-18), 25.7 (C-6), 25.8 (C-19), 30.1 (C-13), 33.3 (C-16), 35.9 (C-10), 36.2 (C-7), 39.4 (C-17), 41.0 (C-8), 41.8 (C-5), 42.7 (C-4), 46.0 (C-12), 48.8 (C-9), 54.5 (C-2), 55.1 (C-1), 68.3 (C-14), 72.6 (C-11); HR-ESIMS *m/z* 485.0662 [M+Na]^+^ (calcd. for C_20_H_32_^79^Br_2_O_2_Na, 485.0661).

Compound **3**: Gummy solid; ^1^H NMR (400 MHz, CDCl_3_) *δ* 0.85 (3H, d, *J* = 6.8 Hz, H_3_-19), 0.94 (3H, d, *J* = 6.8 Hz, H_3_-20), 1.13 (1H, td, *J* = 13.9, 3.0 Hz, H-7b), 1.18 (3H, s, H_3_-15), 1.40 (1H, ddd, *J* = 14.6, 12.3, 5.2 Hz, H-3b), 1.45 (3H, s, H_3_-16), 1.52 (1H, d, *J* = 11.8 Hz, H-9), 1.64 (1H, m, H-12b), 1.67 (1H, m, H-6b), 1.71 (1H, m, H-12a), 1.80 (1H, m, H-4), 1.85 (1H, m, H-10), 1.86 (1H, m, H-7a), 1.88 (1H, m, H-6a), 2.00 (1H, m, H-18), 2.03 (1H, m, H-13β), 2.05 (1H, m, H-3a), 2.49 (1H, qd, *J* = 13.0, 4.8 Hz, H-13α), 3.06 (1H, dd, *J* = 6.6, 4.8 Hz, H-1), 3.37 (1H, m, H-2), 3.38 (1H, d, *J* = 10.1 Hz, H-17b), 3.86 (1H, d, *J* = 10.1 Hz, H-17a), 3.98 (1H, dd, *J* = 13.0, 4.0 Hz, H-14); ^13^C NMR (400 MHz, CDCl_3_, determined through HMBC correlations) *δ* 15.7 (C-15), 18.5 (C-19), 22.1 (C-3), 23.4 (C-20), 24.7 (C-6), 27.0 (C-18), 30.7 (C-13), 33.2 (C-16), 36.6 (C-7), 40.6 (C-8), 41.1 (C-10), 42.1 (C-5), 43.8 (C-17), 44.2 (C-12), 45.4 (C-4), 50.7 (C-9), 53.9 (C-2), 54.3 (C-1), 67.7 (C-14), 72.7 (C-11); HR-ESIMS *m/z* 485.0662 [M+Na]^+^ (calcd. for C_20_H_32_^79^Br_2_O_2_Na, 485.0661).

#### 3.4.2. Synthesis of Analogs **4**–**6**

To a stirred solution of bromosphaerol (**1**) (63 mg, 0.14 mmol) in anhydrous THF (1.2 mL), a BH_3_.THF complex solution (1M in THF) (0.42 mL, 0.422 mmol) was added dropwise at −10 °C, and the reaction mixture was stirred for 3 h at 25 °C. Subsequently, water (0.5 mL) was added dropwise, and the resulting mixture was stirred for 15 min at 25 °C. Following that, NaBO_3_.4H_2_O (43 mg, 0.28 mmol) was added, and the mixture was stirred for 12 h at 25 °C. The precipitated solid was filtered off, washed with THF, and discarded. Subsequently, solid sodium chloride was added to the filtrate, and the mixture was extracted with EtOAc (3 × 20 mL). The combined organic layers were dried over anhydrous Na_2_SO_4_ and concentrated under reduced pressure. The residue was dissolved in anhydrous CH_2_Cl_2_ (2.9 mL), PCC (67 mg, 0.31 mmol) was added, and the resulting mixture was stirred at 25 °C for 2 h. The reaction mixture was diluted with CH_2_Cl_2_ (10 mL), and the mixture was filtered through a pad of celite and silica gel (1:1). The solids were washed with CH_2_Cl_2_ (50 mL), the filtrate was evaporated in vacuo, and the residue was purified by flash column chromatography (elution system: petroleum ether 40–65 °C/acetone, 90:10) to afford **6** (25 mg, 45% yield) and a mixture of **4** and **5** in a 1:0.7 ratio (27 mg, 41% yield) that was subsequently subjected to normal-phase HPLC using cHex/EtOAc (50:50) as eluent to yield **4** (11.1 mg) and **5** (6.7 mg) in pure form.

Compound **4**: Gummy solid; ^1^H NMR (600 MHz, CDCl_3_) *δ* 0.88 (3H, d, *J* = 6.9 Hz, H_3_-19), 0.97 (3H, d, *J* = 6.9 Hz, H_3_-20), 1.24 (1H, m, H-7b), 1.28 (3H, s, H_3_-15), 1.31 (3H, s, H_3_-16), 1.42 (1H, d, *J* = 10.6 Hz, H-9), 1.63 (1H, m, H-12b), 1.67 (1H, m, H-6b), 1.69 (1H, m, H-12a), 1.87 (1H, m, H-6a), 1.93 (1H, m, H-7a), 1.97 (1H, m, H-13β), 2.04 (1H, ddd, *J* = 6.6, 4.2, 2.2 Hz, H-4), 2.13 (1H, m, H-18), 2.31 (1H, m, H-3b), 2.33 (1H, m, H-2b), 2.41 (1H, m, H-13α), 2.47 (1H, m, H-3a), 2.84 (1H, m, H-2a), 2.87 (1H, d, *J* = 10.6 Hz, H-10), 3.65 (1H, dd, *J* = 11.0, 1.8 Hz, H-17b), 3.91 (1H, d, *J* = 11.0 Hz, H-17a), 3.95 (1H, dd, *J* = 12.6, 3.7 Hz, H-14); ^13^C NMR (400 MHz, CDCl_3_, determined through HMBC correlations) *δ* 14.2 (C-15), 19.6 (C-19), 24.9 (C-6), 25.0 (C-20), 26.5 (C-18), 30.0 (C-13), 34.8 (C-16), 36.3 (C-7), 36.2 (C-3), 36.5 (C-10), 38.3 (C-17), 40.9 (C-5), 41.0 (C-8), 44.0 (C-2), 44.1 (C-4), 46.2 (C-12), 51.8 (C-9), 67.9 (C-14), 72.5 (C-11), 212.1 (C-1); HR-ESIMS *m/z* 485.0663 [M+Na]^+^ (calcd. for C_20_H_32_^79^Br_2_O_2_Na, 485.0661).

Compound **5**: Gummy solid; ^1^H NMR (600 MHz, CDCl_3_) *δ* 1.02 (3H, d, *J* = 6.7 Hz, H_3_-20), 1.05 (3H, d, *J* = 6.7 Hz, H_3_-19), 1.22 (1H, m, H-7b), 1.32 (3H, s, H_3_-15), 1.38 (3H, s, H_3_-16), 1.50 (1H, m, H-3b), 1.53 (1H, m, H-2b), 1.59 (1H, m, H-12b), 1.60 (1H, m, H-6b), 1.61 (1H, d, *J* = 10.1 Hz, H-9), 1.67 (1H, m, H-3a), 1.74 (1H, m, H-4), 1.76 (1H, m, H-6a), 1.79 (1H, m, H-12a), 1.83 (1H, m, H-7a), 1.92 (1H, m, H-13β), 1.93 (1H, m, H-2a), 1.98 (1H, m, H-18), 2.29 (1H, t, *J* = 10.1 Hz, H-10), 2.44 (1H, qd, *J* = 13.2, 3.7 Hz, H-13α), 3.49 (1H, dd, *J* = 10.6, 1.4 Hz, H-17b), 3.97 (1H, d, *J* = 10.6 Hz, H-17a), 3.99 (1H, m, H-14), 4.00 (1H, m, H-1); ^13^C NMR (400 MHz, CDCl_3_, determined through HMBC correlations) *δ* 14.4 (C-15), 18.7 (C-3), 21.1 (C-19), 25.3 (C-20), 26.4 (C-18), 27.6 (C-6), 29.9 (C-13), 32.3 (C-16), 33.9 (C-2), 36.7 (C-7), 40.8 (C-17), 41.7 (C-8), 43.1 (C-4), 43.8 (C-5), 45.4 (C-12), 45.9 (C-10), 52.2 (C-9), 67.3 (C-1), 69.9 (C-14), 72.3 (C-11); HR-ESIMS *m/z* 463.0847 [M-H]^−^ (calcd. for C_20_H_33_^79^Br_2_O_2_, 463.0853).

Compound **6**: Gummy solid; ^1^H NMR (600 MHz, CDCl_3_) *δ* 0.94 (3H, d, *J* = 6.9 Hz, H_3_-20), 1.01 (1H, m, H-6b), 1.06 (3H, d, *J* = 6.9 Hz, H_3_-19), 1.16 (3H, s, H_3_-15), 1.22 (1H, m, H-4), 1.25 (1H, m, H-7b), 1.30 (3H, s, H_3_-16), 1.31 (1H, d, *J* = 11.2 Hz, H-9), 1.47 (1H, td, *J* = 13.3, 6.2 Hz, H-2b), 1.54 (1H, ddd, *J* = 14.0, 4.5, 3.0 Hz, H-12b), 1.58 (1H, m, H-3b), 1.63 (1H, ddd, *J* = 14.0, 13.0, 4.6 Hz, H-12a), 1.71 (1H, m, H-2a), 1.81 (1H, ddd, *J* = 14.3, 13.3, 7.3 Hz, H-3a), 1.86 (1H, m, H-7a), 1.88 (1H, m, H-6a), 2.06 (1H, dddd, *J* = 13.0, 4.6, 4.2, 3.0 Hz, H-13β), 2.10 (1H, qd, *J* = 6.9, 2.0 Hz, H-18), 2.18 (1H, d, *J* = 11.2 Hz, H-10), 2.47 (1H, qd, *J* = 13.0, 4.5 Hz, H-13α), 3.58 (1H, d, *J* = 8.3 Hz, H-17b), 3.87 (1H, d, *J* = 8.3 Hz, H-17a), 3.96 (1H, dd, *J* = 13.0, 4.2 Hz, H-14), 4.74 (1H, brd, *J* = 4.8 Hz, H-1); ^13^C NMR (150 MHz, CDCl_3_) *δ* 16.1 (C-15), 18.8 (C-3), 20.2 (C-19), 22.4 (C-6), 26.9 (C-20), 27.3 (C-18), 30.7 (C-13), 32.0 (C-2), 33.9 (C-16), 36.7 (C-7), 40.3 (C-8), 42.0 (C-10), 44.1 (C-12), 48.3 (C-9), 48.5 (C-5), 50.7 (C-4), 68.3 (C-14), 73.0 (C-11), 76.2 (C-17), 81.9 (C-1); HR-ESIMS *m/z* 385.1735 [M+H]^+^ (calcd. for C_20_H_34_^79^BrO_2_, 385.1737).

#### 3.4.3. Synthesis of Analogs **7** and **8**

To a stirred solution of bromosphaerol (**1**) (220 mg, 0.48 mmol) in anhydrous 1,4-dioxane (1.4 mL), a solution of 70% HClO_4_ (0.28 mL, 1.8 mmol) and water (1.4 mL) was added dropwise, followed by the addition of a suspension of *N*-bromoacetamide (0.13 g, 0.95 mmol) in water (0.5 mL). The reaction mixture was stirred for 2 h at 25 °C (the completion of the reaction was monitored by TLC), saturated aqueous Na_2_S_2_O_3_ solution (1 mL) was added, and the resulting mixture was stirred for 30 min. Subsequently, the mixture was poured into ice water and extracted with CH_2_Cl_2_ (3 × 15 mL). The combined organic layers were washed with NaHCO_3_, dried over anhydrous Na_2_SO_4_, and concentrated under reduced pressure. The residue was purified by flash column chromatography (elution system: petroleum ether 40–65 °C/acetone, 90:10) to afford compound **7** (141 mg, 40% yield) and **8** (152 mg, 38%).

Compound **7**: Gummy solid; ^1^H NMR (600 MHz, CDCl_3_) *δ* 0.84 (3H, d, *J* = 6.7 Hz, H_3_-19), 0.99 (3H, d, *J* = 6.7 Hz, H_3_-20), 1.14 (3H, s, H_3_-15), 1.34 (3H, s, H_3_-16), 1.39 (1H, m, H-7b), 1.44 (1H, m, H-6b), 1.50 (1H, m, H-12b), 1.64 (2H, m, H-6a, H-12a), 1.86 (1H, m, H-18), 1.92 (1H, m, H-7a), 2.00 (1H, m, H-4), 2.02 (1H, m, H-13β), 2.15 (1H, s, H-9), 2.24 (1H, m, H-3b), 2.38 (1H, dd, *J* = 13.1, 5.9 Hz, H-3a), 2.45 (1H, qd, *J* = 12.8, 5.7 Hz, H-13α), 3.55 (1H, d, *J* = 11.2 Hz, H-17b), 3.91 (1H, d, *J* = 11.2 Hz, H-17a), 4.02 (1H, dd, *J* = 12.8, 3.7 Hz, H-14), 6.97 (1H, s, H-1); ^13^C NMR (75 MHz, CDCl_3_) *δ* 15.2 (C-15), 18.7, 24.2, 26.1, 26.8, 29.7, 29.7, 33.5, 37.1, 37.2, 41.5, 44.6, 45.1, 46.9, 52.8 (C-9), 66.8 (C-14), 71.7 (C-11), 130.0 (C-1), 161.1 (C-10), 200.0 (C-2); HR-ESIMS *m/z* 461.0688 [M+H]^+^ (calcd. for C_20_H_31_^79^Br_2_O_2_, 461.0685).

Compound **8**: Gummy solid; ^1^H NMR (600 MHz, CDCl_3_) *δ* 0.97 (3H, d, *J* = 6.0 Hz, H_3_-19), 1.19 (3H, s, H_3_-16), 1.19 (3H, d, *J* = 6.0 Hz, H_3_-20), 1.21 (3H, s, H_3_-15), 1.23 (1H, m, H-7b), 1.32 (1H, d, *J* = 12.7 Hz, H-9), 1.64 (1H, ddd, *J* = 13.6, 4.1, 2.7 Hz, H-7a), 1.72 (1H, dd, *J* = 13.2, 7.7 Hz, H-12b), 1.78 (1H, m, H-6b), 1.83 (1H, m, H-4), 1.91 (1H, m, H-18), 2.03-2.07 (3H, m, H-3b, H-6a, H-12a), 2.22 (1H, m, H-13b), 2.32 (1H, ddt, *J* = 16.3, 11.0, 8.3 Hz, H-13a), 2.49 (1H, ddd, *J* = 16.8, 3.2, 1.4 Hz, H-3a), 2.58 (1H, dd, *J* = 12.7, 11.8 Hz, H-10), 3.34 (1H, dd, *J* = 11.8, 3.2 Hz, H-1), 3.47 (1H, dd, *J* = 10.5, 2.0 Hz, H-17b), 3.78 (1H, d, *J* = 10.5 Hz, H-17a), 3.87 (1H, t, *J* = 8.3 Hz, H-14), 4.58 (1H, q, *J* = 3.2 Hz, H-2); ^13^C NMR (75 MHz, CDCl_3_) *δ* 15.7 (C-15), 23.9 (C-20), 26.1 (C-19), 26.7 (C-18), 28.9 (C-16), 29.6 (C-6), 30.2 (C-3), 31.2 (C-13), 32.8 (C-12), 38.4 (C-17), 39.2 (C-7), 42.7 (C-8), 42.6 (C-5), 43.5 (C-10), 46.2 (C-4), 51.7 (C-2), 52.9 (C-9), 59.4 (C-14), 72.9 (C-1), 80.9 (C-11); HR-ESIMS *m/z* 546.9822 [M+Na]^+^ (calcd. for C_20_H_31_^79^Br_3_OΝa, 546.9817).

#### 3.4.4. Synthesis of Analogs **9** and **10**

To a solution of bromosphaerol (**1**) (100 mg, 0.216 mmol), a catalytic amount of trimethylsilyl triflate (2 μL, 0.011 mmol) was added in acetic anhydride (0.43 mL, 0.432 mmol) at −10 °C. The reaction was stirred for 1 h, quenched with water (1 mL), and extracted with CH_2_Cl_2_ (3 × 15 mL). The combined organic layers were washed with saturated aqueous NaHCO_3_ solution (5 mL), dried over anhydrous Na_2_SO_4_, and evaporated under reduced pressure. Compounds **9** (65 mg, 70% yield) and **10** (9 mg, 10% yield) were isolated by flash column chromatography (elution system: petroleum ether 40–65 °C/acetone 90:10). Comparison of the spectroscopic and physical characteristics of **9** and **10** with those reported in the literature [38] allowed for their identification.

#### 3.4.5. Synthesis of Analog **11**

To a solution of compound **7** (28 mg, 0.061 mmol) and triethylphosphonoacetate (0.14 g, 0.61 mmol) in a mixture of abs. EtOH and anhydrous THF (1:1) (0.40 mL), a solution of sodium ethanolate, prepared from Na (14 mg, 0.61 mmol) in abs. EtOH (0.29 mL) was added dropwise. The reaction mixture was stirred for 12 h at 25 °C, quenched with water (5.0 mL), and diluted with diethyl ether (10 mL). Subsequently, the aqueous layer was extracted with diethyl ether (3 × 15 mL). The combined organic layers were washed successively with 5% aqueous HCl solution, 5% aqueous NaHCO_3_ solution, and water, dried over anhydrous Na_2_SO_4_, and evaporated in vacuo. Compound **11** (29.5 mg, 90% yield) was isolated as a mixture of *Ε*,*Ζ* geometric isomers after purification by flash column chromatography (elution system: hexane/acetone 90:10).

Compound **11**: Gummy solid; ^1^H NMR (600 MHz, CDCl_3_) *δ* 0.85 and 0.90 (3H, 2d, *J* = 6.6 Hz), 1.00 and 1.03 (3H, 2d, *J* = 6.9 Hz), 1.12 and 1.15 (3H, 2s), 1.25-1.44 (7H, m), 1.63-2.10 (9H, m), 2.22-2.55 (2H, m), 3.47-3.71 (2H, m), 3.90 (1H, d, *J* = 11.2 Hz), 4.02-4.19 (3H, m), 5.54 and 5.67 (1H, 2s), 7.00 (0.4H, s), 8.19 (0.6H, s); ^13^C NMR (75 MHz, CDCl_3_) *δ* 14.6, 15.0, 15.5, 15.4, 18.9, 18.8, 21.4, 24.7, 24.6, 26.5, 26.3, 27.3, 27.2, 28.4, 29.7, 29.8, 29.9, 30.1, 30.5, 37.8, 38.1, 38.2, 41.7, 42.9, 44.4, 44.5, 44.9, 45.1, 46.2, 46.7, 52.6, 52.8, 59.6, 59.7, 67.4, 67.9, 72.2, 72.3, 112.7, 115.2, 126.5, 130.8, 147.1, 147.5, 153.0, 154.3, 166.8, 167.5; HR-ESIMS *m/z* 553.0921 [M+Na]^+^ (calcd. for C_24_H_36_^79^Br_2_O_3_Na 553.0923).

#### 3.4.6. Synthesis of Analogs **12**–**16**

To a solution of compound **7** (0.05 mmol) in anhydrous pyridine (0.45 mL), the appropriate alkoxyalkylamine salt (0.1 mmol) was added, and the reaction mixture was stirred for 12 h at 25 °C. Subsequently, the solvent was evaporated under reduced pressure, and to the residue, CH_2_Cl_2_ (10 mL) and water (5 mL) were added. The aqueous layer was extracted with CH_2_Cl_2_ (2 × 10 mL), and the combined organic layers were dried over anhydrous Na_2_SO_4_ and concentrated under reduced pressure. The final products were isolated after purification by flash column chromatography.

Following the general procedure described above, using compound **7** (21 mg, 0.045 mmol), pyridine (0.4 mL), and hydroxylamine hydrochloride (6.3 mg, 0.09 mmol), compound **12** was obtained (20.6 mg, 95% yield, mixture of *E*,*Z* geometric isomers) after purification by flash column chromatography (elution system: petroleum ether 40–65 °C/acetone, 80:20).

Compound **12**: Gummy solid; ^1^H NMR (600 MHz, CDCl_3_) *δ* 0.87 and 0.89 (3H, 2d, *J* = 6.7 Hz), 1.04–1.02 (3H, m), 1.13 and 1.14 (3H, 2s), 1.33–1.45 (2H, m), 1.43 and 1.48 (3H, 2d, *J* = 6.9 Hz), 1.61–1.65 (2H, m), 1.81–2.27 (8H, m), 2.40–2.55 (1H, m), 3.49–3.56 (1H, m), 3.87–3.94 (1H, m), 4.03 (1H, dd, *J* = 12.7, 3.7 Hz), 6.98 (0.67H, s), 7.31–7.33 (0.67H, bs), 7.57 (0.33H, s), 7.70 (0.33H, t, *J* = 7.7 Hz); ^13^C NMR (75 MHz, CDCl_3_) *δ* 15.4, 17.4, 18.8, 18.9, 24.0, 24.5, 24.6, 26.3, 26.4, 27.2, 27.3, 29.8, 29.9, 30.0, 30.1, 37.6, 37.7, 37.8, 38.1, 40.6, 42.6, 44.3, 44.9, 46.3, 47.2, 52.5, 52.7, 67.5, 67.7, 72.1, 118.1, 124.4, 136.4, 146.5, 149.6, 149.7; HR-ESIMS *m/z* 476.0796 [M+H]^+^ (calcd. for C_20_H_32_^79^Br_2_NO_2_, 476.0794), *m/z* 498.0617 [M+Na]^+^ (calcd. for C_20_H_31_^79^Br_2_NO_2_Νa, 498.0614).

Following the general procedure described above, using compound **7** (20 mg, 0.043 mmol), pyridine (0.4 mL), and methoxyamine hydrochloride (7.2 mg, 0.086 mmol), compound **13** was obtained (21 mg, 98% yield, mixture of *E*,*Z* geometric isomers) after purification by flash column chromatography (elution system: petroleum ether 40–65 °C/acetone, 90:10).

Compound **13**: Gummy solid; ^1^H NMR (600 MHz, CDCl_3_) *δ* 0.86 and 0.88 (3H, 2d, *J* = 6.7 Hz), 1.02–1.04 (3H, m), 1.14 and 1.15 (3H, 2s), 1.34–1.43 (2H, m), 1.39 and 1.42 (3H, 2s), 1.58–1.68 (2H, m), 1.78–2.31 (8H, m), 2.45–2.51 (1H, m) 3.49–3.54 (1H, m), 3.86 and 3.92 (3H, 2s), 3.85–3.90 (1H, m), 4.03 (1H, dd, *J* = 12.7, 3.9 Hz), 6.96 (0.29H, s), 7.46 (0.71H, s); ^13^C NMR (75 MHz, CDCl_3_) *δ* 15.4, 15.5, 18.0, 18.9, 24.1, 24.6, 24.7, 26.3, 26.4, 27.1, 27.2, 29.9, 29.96, 30.0, 37.6, 37.7, 37.76, 37.8, 38.1, 40.6, 42.6, 44.3, 44.4, 44.9, 46.2, 47.2, 52.4, 52.7, 61.8, 61.9, 67.4, 67.7, 72.17, 72.20, 118.6, 124.4, 146.5, 149.7, 156.2, 156.3; HR-ESIMS *m/z* 490.0952 [M+H]^+^ (calcd. for C_21_H_34_^79^Br_2_NO_2_, 490.0951), *m/z* 512.0772 [M+Na]^+^ (calcd. for C_21_H_33_^79^Br_2_NO_2_Νa, 512.0770).

Following the general procedure described above, using compound **7** (25.5 mg, 0.055 mmol), pyridine (0.45 mL), and *O-*(carboxymethyl)hydroxylamine hemihydrochloride (24.1 mg, 0.22 mmol), compound **14** was obtained (28.3 mg, 96% yield, mixture of *E*,*Z* geometric isomers) after purification by flash column chromatography (elution system: CH_2_Cl_2_/MeOH, 90:10).

Compound **14**: Gummy solid; ^1^H NMR (600 MHz, CDCl_3_/CD_3_OD) *δ* 0.81 and 0.84 (3H, 2d, *J* = 6.8 Hz), 0.97 and 0.99 (3H, 2d, *J* = 6.9 Hz), 1.07 and 1.09 (3H, 2s), 1.29 and 1.35 (3H, 2s), 1.32–1.40 (2H, m), 1.51–1.66 (2H, m), 1.76–2.16 (8H, m), 2.41–2.49 (1H, m), 3.45–3.50 (1H, m), 3.85–3.89 (1H, m), 3.98–4.02 (1H, m), 4.46–4.47 (2H, m), 6.91 (0.42H, s), 7.42 (0.58H, s); ^13^C NMR (150 MHz, CDCl_3_/CD_3_OD) *δ* 15.4, 15.5, 18.4, 18.8, 18.9, 21.9, 24.0, 24.5, 24.6, 26.3, 26.4, 27.2, 27.3, 29.88, 29.90, 29.94, 30.00, 37.5, 37.6, 37.7, 37.9, 40.5, 42.5, 44.4, 44.5, 44.7, 44.8, 46.3, 47.2, 52.6, 52.8, 67.2, 67.4, 70.2, 70.3, 72.1, 72.2, 118.4, 123.6, 148.5, 151.5, 156.1, 158.1, 173.9, 174.4; HR-ESIMS *m/z* 556.0674 [M+Na]^+^ (calcd. for C_22_H_33_^79^Br_2_NO_4_Νa, 556.0669).

Following the general procedure described above, using compound **7** (30 mg, 0.065 mmol), pyridine (0.5 mL), and methyl 2-(aminooxy)acetate hydrochloride (27.3 mg, 0.26 mmol) [39], compound **15** was obtained (20.5 mg, 56% yield, mixture of *E*,*Z* geometric isomers) after purification by flash column chromatography (elution system: petroleum ether 40–65 °C/acetone, 90:10).

Compound **15**: Gummy solid; ^1^H NMR (300 MHz, CDCl_3_) *δ* 0.85 and 0.89 (3H, 2d, *J* = 6.7 Hz), 1.02 and 1.04 (3H, 2d, *J* = 7.2 Hz), 1.14 and 1.16 (3H, 2s), 1.37 and 1.42 (3H, 2s), 1.37–1.45 (2H, m), 1.59–1.67 (2H, m), 1.78–2.26 (8H, m), 2.40–2.55 (1H, m), 3.48–3.56 (1H, m), 3.77 and 3.75 (3H, 2s), 3.86–3.93 (1H, m), 4.03 (1H, dd, *J* = 12.4, 3.7 Hz), 4.57–4.66 (2H, m), 6.93 (0.38H, s), 7.55 (0.62H, s); ^13^C NMR (75 MHz, CDCl_3_) *δ* 15.4, 15.5, 18.3, 18.8, 18.9, 24.0, 24.5, 24.7, 26.3, 26.4, 27.1, 27.3, 29.9, 29.93, 29.97, 30.0, 37.64, 37.67, 37.7, 38.0, 40.5, 42.4, 44.3, 44.4, 44.9, 46.3, 47.1, 52.0, 52.1, 52.5, 52.7, 67.4, 67.5, 70.4, 70.6, 72.1, 72.2, 118.7, 124.0, 147.3, 150.0, 155.0, 157.8, 170.8, 171.0; HR-APCIMS *m/z* 548.1008 [M+H]^+^ (calcd. for C_2__3_H_3__6_^79^Br_2_NO_4_, 548.1006).

Following the general procedure described above, using compound **7** (21 mg, 0.045 mmol), pyridine (0.4 mL), and *O*-(*N*,*N*-dimethylaminoethyl)hydroxylamine hydrochloride [40] (32 mg, 0.18 mmol), compound **16** was obtained (17.5 mg, 81% yield, mixture of *E*,*Z* geometric isomers) after purification by flash column chromatography (elution system: CH_2_Cl_2_/MeOH, 95:5).

Compound **16**: Gummy solid; ^1^H NMR (300 MHz, CDCl_3_/CD_3_OD) *δ* 0.78 (3H, d, *J* = 7.0 Hz), 0.94 (3H, d, *J* = 6.9 Hz), 1.05 (3H, s), 1.27 (3H, s), 1.17–2.11 (11H, m), 2.33 and 2.41 (6H, 2 s), 2.33–2.47 (2H, m), 2.71–2.83 (2H, m), 3.41–3.47 (1H, m), 3.81–3.87 (1H, m), 3.97 (1H, dd, *J* = 12.7, 3.7 Hz), 4.12–4.25 (2H, m), 6.89 (0.34H, s), 7.41 (0.66H, s); ^13^C NMR (75 MHz, CDCl_3_/CD_3_OD) *δ* 15.0, 15.1, 18.6, 23.8, 24.3, 24.4, 26.1, 26.2, 27.1, 29.0, 29.3, 29.6, 29.8, 29.9, 37.3, 37.4, 37.6, 37.9, 40.6, 42.5, 44.3, 44.4, 44.5, 44.8, 45.2, 46.1, 47.0, 52.5, 52.7, 57.3, 57.5, 67.8, 67.9, 69.9, 70.9, 118.4, 123.7, 151.0, 154.8; HR-APCIMS *m/z* 547.1533 [M+H]^+^ (calcd. for C_24_H_41_^79^Br_2_N_2_O_2_, 547.1529).

### 3.5. Evaluation of Settlement Inhibitory Activity

Cypris larvae were obtained from laboratory cultures of the crustacean cirriped *A. amphitrite* brood stock. Twenty to thirty adult barnacles were reared in 800 mL aerated beakers containing filtered natural seawater (FNSW) at 20 ± 1 °C, with a 16 h:8 h light:dark (L:D) cycle. They were fed every two days with nauplii of *Artemia salina* (100 mL, 20–35 larvae/mL) and *Tetraselmis suecica* (100 mL, 2 × 10^5^ cells/mL). Twenty beakers containing adults reared under the above-mentioned conditions produced nauplii throughout the year. Nauplii were collected with a 5 mL pipette by positioning the beaker near a light source and reared in 500 mL beakers containing 0.22 µm FNSW gently aerated at 28 ± 1 °C with a 16 h:8 h L:D cycle. Nauplii were fed every 48 h with *T. suecica* (5 × 10^5^ cells/mL) until, after 5–6 days, they reached the cyprid stage.

Newly metamorphosed cyprids were filtered and maintained in 0.22 µm FNSW at 6 °C for 4 days before being used in settlement assays [41]. Settlement tests were performed by adding 15–20 cyprids (for each replicate) to 24-well polystyrene plates containing 2 mL of bromosphaerol derivatives at different concentrations (0, 0.5, 5, and 50 mg/L). Four replicates were prepared for each concentration of each derivative, and the reported results are the mean values of the four replicates. The 24-well plates were stored for 72 h at 28 °C with a 16:8 L:D cycle. After 24, 48, and 72 h, the number of settled, non-settled, and dead larvae was measured under a stereomicroscope. EC_50_ values (concentration of bromosphaerol derivatives causing 50% settlement inhibition to exposed organisms) were calculated with the results obtained after 72 h. Additionally, at the same time, LC_50__(cypris)_ values were calculated as the concentration of bromosphaerol derivatives causing 50% mortality to the exposed organisms.

### 3.6. Evaluation of Toxicity

Acute environmental toxicity of bromosphaerol derivatives was tested by using stage II nauplii of *A. amphitrite*. Nauplii were obtained from adult brood stock as described above, collected, and immediately filtered in 0.22 µm FNSW. The toxicity assay was set within 2–4 h from nauplii collection. The test was performed by adding 15 to 25 stage II nauplii to 24-well polystyrene plates containing 2 mL of bromosphaerol derivatives at different concentrations (0, 0.5, 5, and 50 mg/L). Four replicates were prepared for each concentration of each derivative, and the reported results are the mean values of the four replicates. The plates were stored for 48 h at 20 °C with a 16:8 L:D cycle. After 24 and 48 h, the number of dead larvae was observed under a stereomicroscope. LC_50(nauplii)_ values were calculated as the concentration of bromosphaerol derivatives causing 50% mortality to the exposed organisms after 48 h of contact.

### 3.7. Statistical Analysis

Settlement inhibition (EC_50_) at 72 h and mortality (LC_50_) values at 48 h (for nauplii) and 72 h (for cyprids) were calculated using trimmed Spearman–Karber analysis [42]. The therapeutic ratio (TN) was defined as LC_50_/EC_50_. This index was calculated using mortality values measured for larvae at naupliar stage (TR_N_) and for larvae at cypris stage (TR_C_).

## 4. Conclusions

Following different synthetic routes, we successfully synthesized 15 structural analogs (**2**–**16**) of bromosphaerol (**1**) decorated with different functional groups. The anti-settlement activity (EC_50_) and the degree of toxicity (LC_50_) of the bromosphaerol derivatives were evaluated using cyprids and nauplii of *A. amphitrite* as a model organism. Derivatives **2**, **4**, and **6**–**16** showed diverse levels of antifouling activity, with four of them (**9**, **13**, **15**, and **16**) displaying a bioactivity comparable to the one of the naturally occurring molecule. Among them, compounds **9** and **13** can be considered as well-performing antifoulants, exerting their activity through a non-toxic mechanism. The chemical diversity of these derivatives provides new insights on structure–activity relationship studies regarding settlement inhibition, helping to understand the antifouling mechanism of bromosphaerol (**1**), while potentially serving as bioinspired alternatives in antifouling coatings and paints.

## Figures and Tables

**Figure 1:**
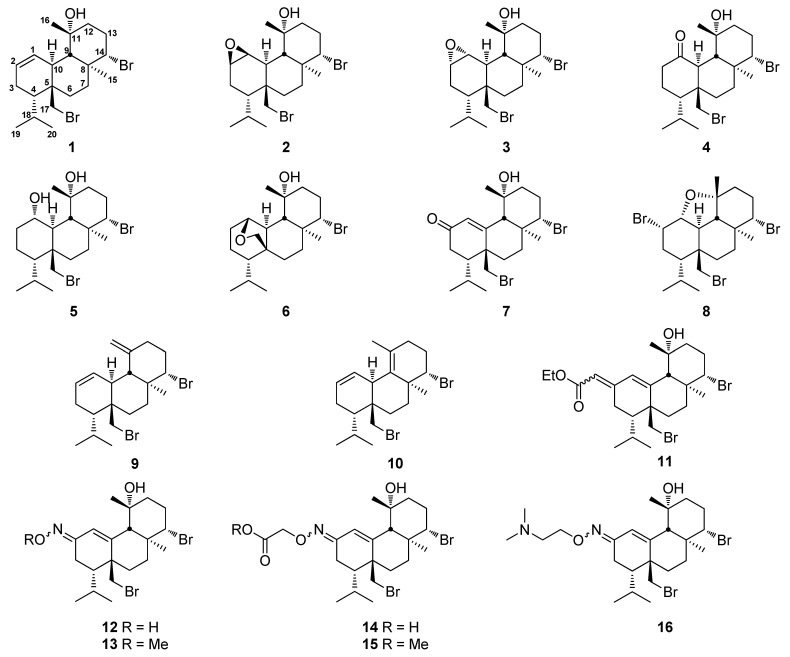
Bromosphaerol (**1**) and its synthetic derivatives **2**–**16**.

**Table 1:** EC_50_, LC_50(cypris)_, and LC_50(nauplii)_ values (in mg/L, with 95% confidence limits in parentheses) for cypris larvae settlement inhibition, cypris larvae mortality, and naupliar mortality of *Amphibalanus amphitrite* after exposure for 72, 72, and 48 h, respectively, to bromosphaerol derivatives **2**, **4**, and **6**–**16**, as well as therapeutic ratios for bromosphaerol derivatives **2**, **4**, and **6**–**16** calculated against both LC_50(nauplii)_ values from naupliar toxicity test (TR_N_) and LC_50(cypris)_ values from cyprids toxicity assay (TR_C_).

Compounds	EC_50_ (72 h) Cypris Larvae Settlement Inhibition	LC_50(cypris)_ (72 h) Cypris Larvae Mortality	LC_50(nauplii)_ (48 h) Naupliar Mortality	TR_N_ (LC_50(nauplii)_/EC_50_)	TR_C_ (LC_50(cypris)_/EC_50_)
**1**	0.23 (0.17–0.30)	>100	3.63 (3.05–4.33)	15.78	434.78
**2**	10.44 ^†^	25.2 ^†^	2.75 (2.47–3.07)	0.26	2.39
**4**	7.19 (4.57–11.3)	>50	1.27 ^†^	0.17	6.95
**6**	7.53 (5.82–9.73)	10.2 ^†^	7.57 (6.10–9.38)	1.00	1.32
**7**	8.75 (6.75–11.34)	>50	11.53 (9.53–13.95)	1.31	>5.71
**8**	>50	>50	>50	n.d. ^‡^	n.d. ^‡^
**9**	<0.5	>50	1.19 ^†^	2.38	>100
**10**	3.87 ^†^	>50	2.31 ^†^	0.59	12.90
**11**	>50	>50	21.64 (16.57–28.28)	n.d. ^‡^	n.d. ^‡^
**12**	>50	>50	1.21 ^†^	n.d. ^‡^	n.d. ^‡^
**13**	<0.5	>50	1.26 ^†^	2.52	>100
**14**	>50	>50	>50	n.d. ^‡^	n.d. ^‡^
**15**	<0.5	12.5 ^†^	1.81 ^†^	3.62	25.00
**16**	<0.5	2.7 ^†^	1.36 ^†^	2.72	5.40

^†^ Confidence limits could not be defined. ^‡^ TR values were not calculated for derivatives with high EC_50_ values (not considered promising as antifoulants).

## Data Availability

The data presented in this study are available in Supplementary Material.

## Supplementary Materials

The following are available online at https://www.mdpi.com/article/10.3390/md20010007/s1, Figures S1–S46: 1D and 2D NMR spectra of compounds **1**–**16**.
