# Supplementary material for: Synthesis and Antifouling Activity Evaluation of Analogs of Bromosphaerol, a Brominated Diterpene Isolated from the Red Alga Sphaerococcus coronopifolius
Source: Mar Drugs. 2021 Dec 22;20(1):7. doi: 10.3390/md20010007 (PMC8781426; doi:10.3390/md20010007)
Supplement: Supplementary file 1 [file marinedrugs-20-00007-s001.zip › marinedrugs-1498687-supplementary.pdf]

Supplementary Materials for

Synthesis and Antifouling Activity Evaluation of Analogues of Bromosphaerol, a Brominated Diterpene Isolated from the Red Alga *Sphaerococcus coronopifolius*

Kyriakos C. Prousis [1], Stefanos Kikionis [2], Efstathia Ioannou [2], Silvia Morgana [3], Marco Faimali [3], Veronica Piazza [3,*], Theodora Calogeropoulou [1,*] and Vassilios Roussis [2,*]

[1] Institute of Chemical Biology, National Hellenic Research Foundation, 48 Vassileos Constantinou Avenue, 11653 Athens, Greece; kyrprous@eie.gr (K.C.P.)

[2] Section of Pharmacognosy and Chemistry of Natural Products, Department of Pharmacy, National and Kapodistrian University of Athens, Panepistimiopolis Zografou, 15771 Athens, Greece; skikionis@pharm.uoa.gr (S.K.); eioannou@pharm.uoa.gr (E.I.)

[3] Institute for the Study of Anthropic Impacts and Sustainability in Marine Environment (IAS), National Research Council (CNR), Via De Marini 6, 16149 Genova, Italy; silvia.morgana@ias.cnr.it (S.M.); marco.faimali@ias.cnr.it (M.F.)

* Correspondence: veronica.piazza@ias.cnr.it (V.P.); tcalog@eie.gr (T.C.); roussis@pharm.uoa.gr (V.R.); Tel.: +39-010-6475409 (V.P.); +30-210-7273833 (T.C.); +30-210-7274592 (V.R.)

Table of Contents

Figure S1. ^{1}H NMR spectrum ($CDCl_3$, 400 MHz) of compound **1**. 4

Figure S2. ^{13}C NMR spectrum ($CDCl_3$, 100 MHz) of compound **1**. 4

Figure S3. ^{1}H NMR spectrum ($CDCl_3$, 600 MHz) of compound **2**. 5

Figure S4. ^{13}C NMR spectrum ($CDCl_3$, 75 MHz) of compound **2**. 5

Figure S5. HSQC spectrum ($CDCl_3$, 600 MHz) of compound **2**. 6

Figure S6. COSY spectrum ($CDCl_3$, 600 MHz) of compound **2**. 6

Figure S7. NOESY spectrum ($CDCl_3$, 600 MHz) of compound **2**. 7

Figure S8. ^{1}H NMR spectrum ($CDCl_3$, 400 MHz) of compound **3**. 7

Figure S9. HSQC spectrum ($CDCl_3$, 400 MHz) of compound **3**. 8

Figure S10. HMBC spectrum ($CDCl_3$, 400 MHz) of compound **3**. 8

Figure S11. COSY spectrum ($CDCl_3$, 400 MHz) of compound **3**. 9

Figure S12. ^{1}H NMR spectrum ($CDCl_3$, 600 MHz) of compound **4**. 9

Figure S13. HSQC spectrum ($CDCl_3$, 600 MHz) of compound **4**. 10

Figure S14. HMBC spectrum ($CDCl_3$, 400 MHz) of compound **4**. 10

Figure S15. COSY spectrum ($CDCl_3$, 400 MHz) of compound **4**. 11

Figure S16. ^{1}H NMR spectrum ($CDCl_3$, 600 MHz) of compound **5**. 11

Figure S17. HSQC spectrum ($CDCl_3$, 600 MHz) of compound **5**. 12

Figure S18. HMBC spectrum ($CDCl_3$, 400 MHz) of compound **5**. 12

Figure S19. COSY spectrum ($CDCl_3$, 400 MHz) of compound **5**. 13

Figure S20. ^{1}H NMR spectrum ($CDCl_3$, 600 MHz) of compound **6**. 13

Figure S21. ^{13}C NMR spectrum ($CDCl_3$, 150 MHz) of compound **6**. 14

Figure S22. HSQC spectrum ($CDCl_3$, 600 MHz) of compound **6**. 14

Figure S23. HMBC spectrum ($CDCl_3$, 600 MHz) of compound **6**. 15

Figure S24. COSY spectrum ($CDCl_3$, 600 MHz) of compound **6**. 15

Figure S25. ^{1}H NMR spectrum ($CDCl_3$, 600 MHz) of compound **7**. 16

Figure S26. ^{13}C NMR spectrum ($CDCl_3$, 75 MHz) of compound **7**. 16

Figure S27. COSY spectrum ($CDCl_3$, 600 MHz) of compound **7**. 17

Figure S28. ^{1}H NMR spectrum ($CDCl_3$, 600 MHz) of compound **8**. 17

Figure S29. ^{13}C NMR spectrum ($CDCl_3$, 75 MHz) of compound **8**. 18

Figure S30. HSQC spectrum ($CDCl_3$, 600 MHz) of compound **8**. 18

Figure S31. HMBC spectrum ($CDCl_3$, 600 MHz) of compound **8**. 19

Figure S32. COSY spectrum ($CDCl_3$, 600 MHz) of compound **8**. 19

Figure S33. NOESY spectrum ($CDCl_3$, 600 MHz) of compound **8**........ 20

Figure S34. ^1H NMR spectrum ($CDCl_3$, 600 MHz) of compound **9**. 20

Figure S35. ^1H NMR spectrum ($CDCl_3$, 600 MHz) of compound **10**. 21

Figure S36. ^1H NMR spectrum ($CDCl_3$, 600 MHz) of compound **11** (mixture of *E,Z* geometrical isomers). 21

Figure S37. ^{13}C NMR spectrum ($CDCl_3$, 75 MHz) of compound **11** (mixture of *E,Z* geometrical isomers). 22

Figure S38. ^1H NMR spectrum ($CDCl_3$, 600 MHz) of compound **12** (mixture of *E,Z* geometrical isomers). 22

Figure S39. ^{13}C NMR spectrum ($CDCl_3$, 75 MHz) of compound **12** (mixture of *E,Z* geometrical isomers). 23

Figure S40. ^1H NMR spectrum ($CDCl_3$, 600 MHz) of compound **13** (mixture of *E,Z* geometrical isomers). 23

Figure S41. ^{13}C NMR spectrum ($CDCl_3$, 75 MHz) of compound **13** (mixture of *E,Z* geometrical isomers). 24

Figure S42. ^1H NMR spectrum ($CDCl_3$ / CD_3OD, 600 MHz) of compound **14** (mixture of *E,Z* geometrical isomers)........ 24

Figure S43. ^{13}C NMR spectrum ($CDCl_3$ / CD_3OD, 150 MHz) of compound **14** (mixture of *E,Z* geometrical isomers)........ 25

Figure S44. ^1H NMR spectrum ($CDCl_3$, 300 MHz) of compound **15** (mixture of *E,Z* geometrical isomers). 25

Figure S45. ^{13}C NMR spectrum ($CDCl_3$, 75 MHz) of compound **15** (mixture of *E,Z* geometrical isomers). 26

Figure S46. ^1H NMR spectrum ($CDCl_3$ / CD_3OD, 300 MHz) of compound **16** (mixture of *E,Z* geometrical isomers)........ 26

Figure S47. ^{13}C NMR spectrum ($CDCl_3$ / CD_3OD, 75 MHz) of compound **16** (mixture of *E,Z* geometrical isomers)........ 27

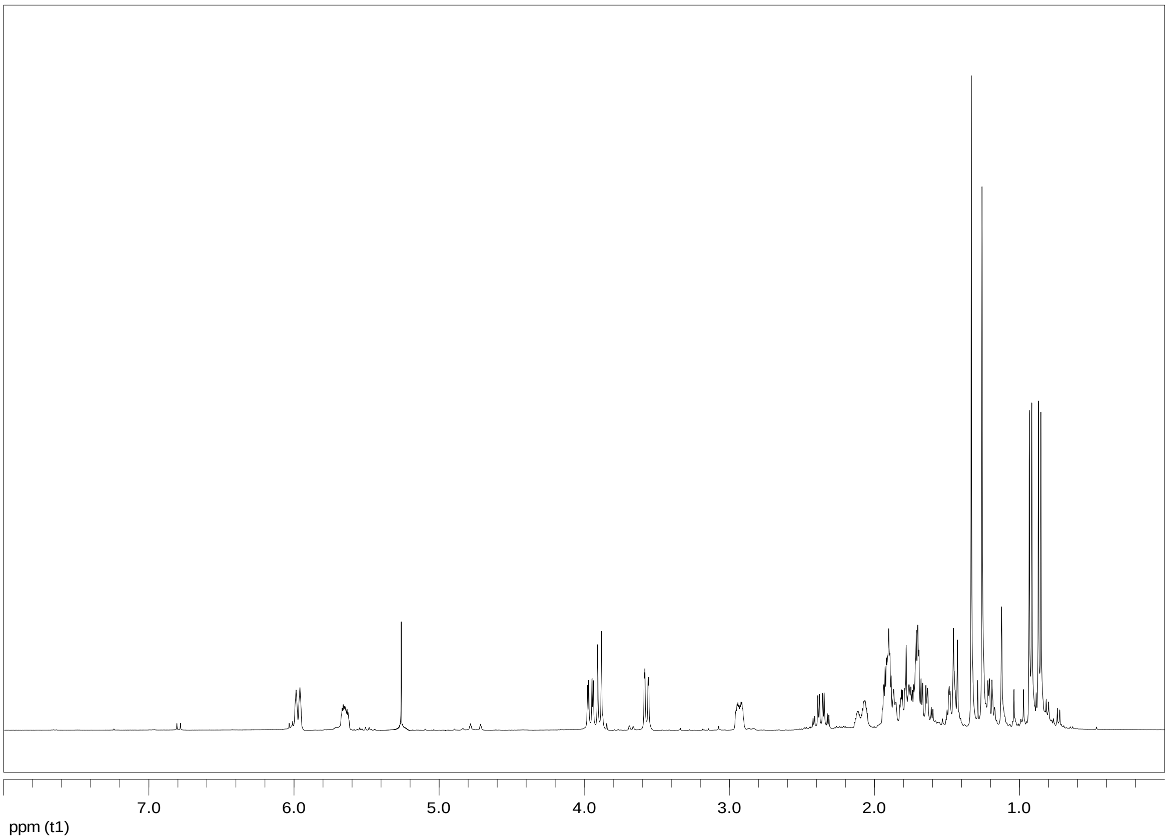

Figure S1. ^{1}H NMR spectrum ($CDCl_3$, 400 MHz) of compound **1**.

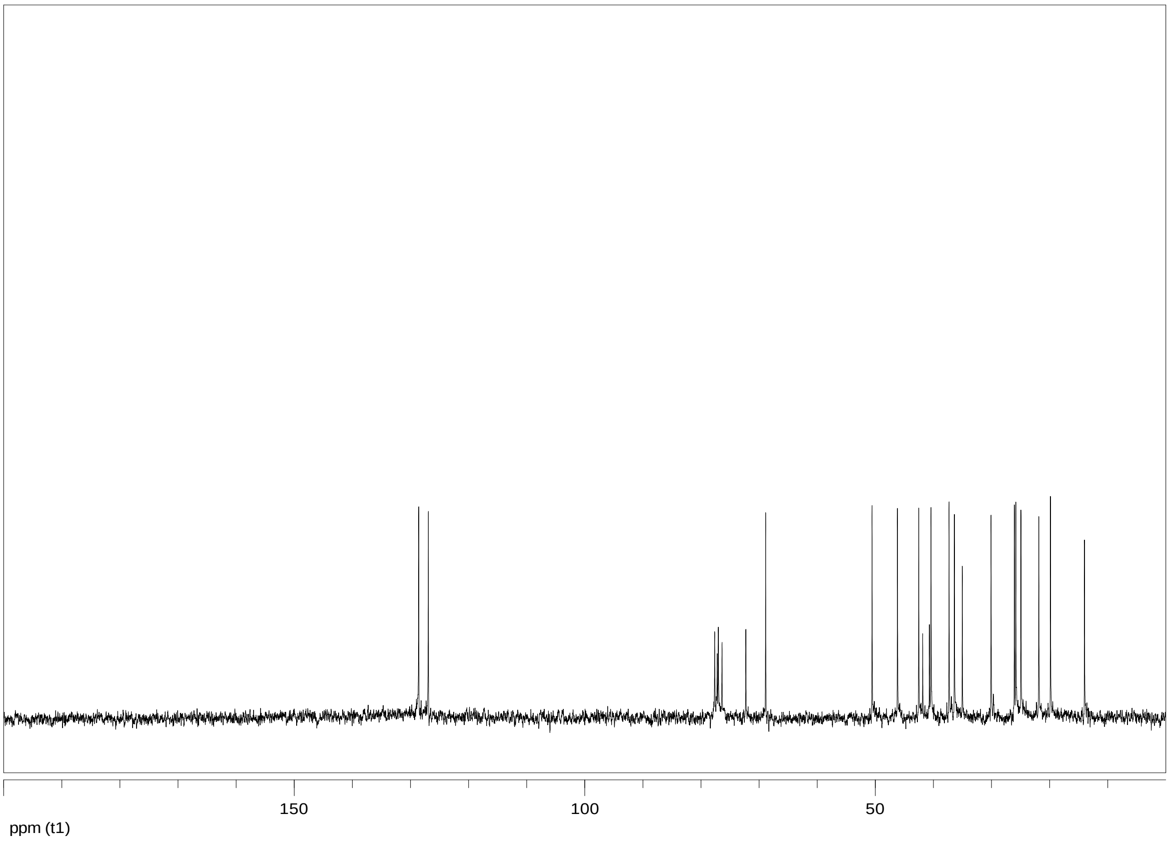

Figure S2. ^{13}C NMR spectrum ($CDCl_3$, 100 MHz) of compound **1**.

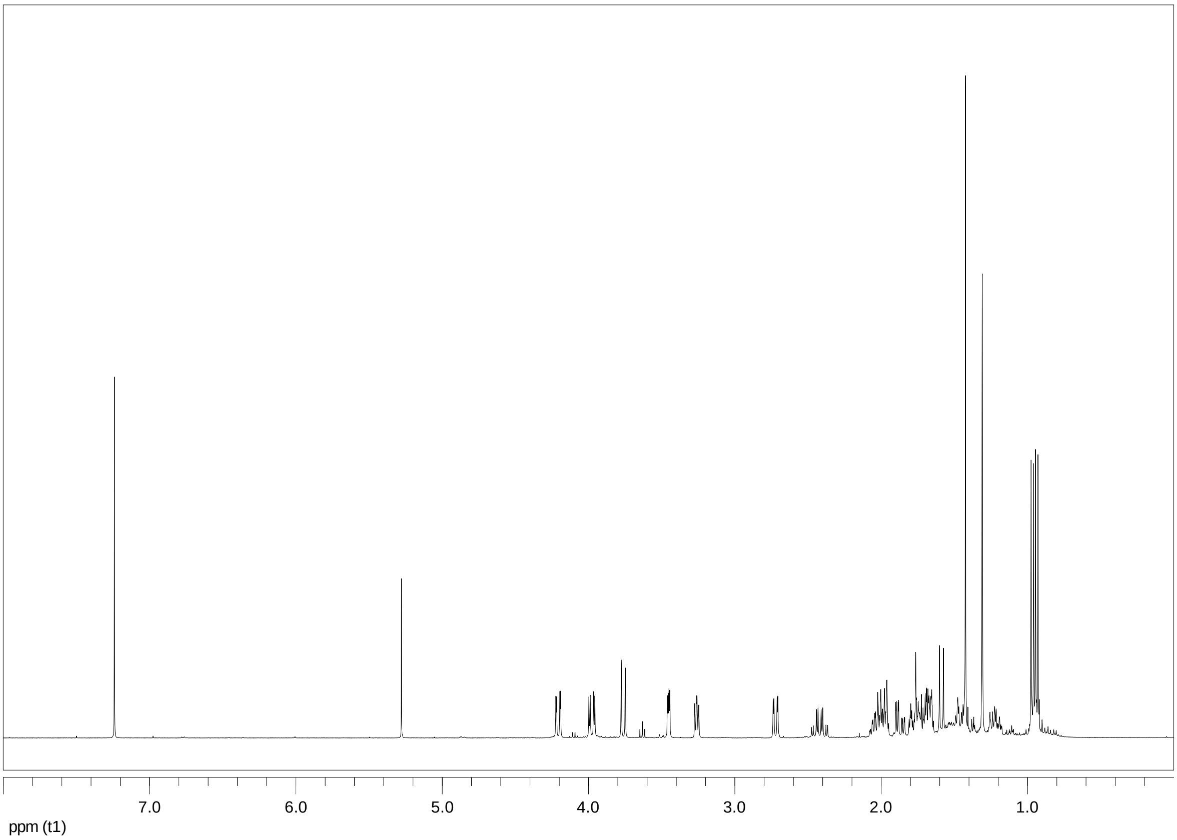

Figure S3. ^{1}H NMR spectrum ($CDCl_3$, 600 MHz) of compound **2**.

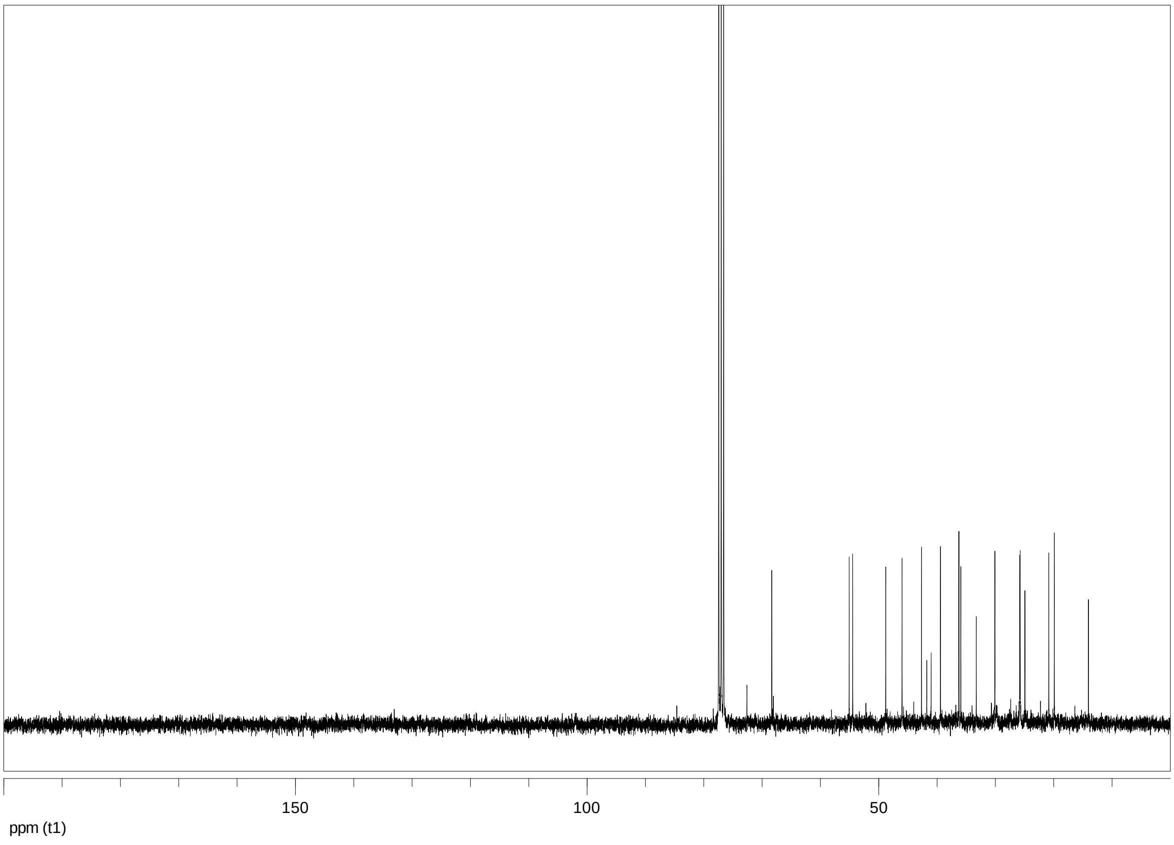

Figure S4. ^{13}C NMR spectrum ($CDCl_3$, 75 MHz) of compound **2**.

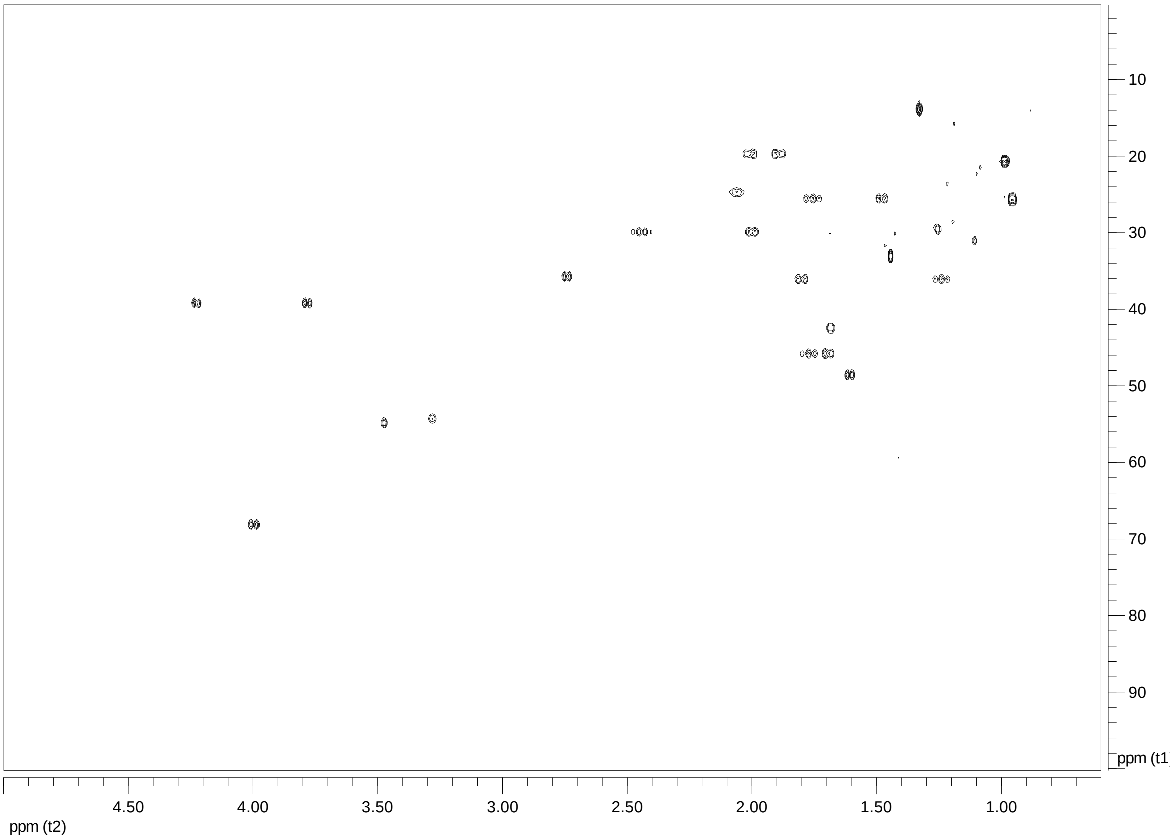

Figure S5. HSQC spectrum ($CDCl_3$, 600 MHz) of compound **2**.

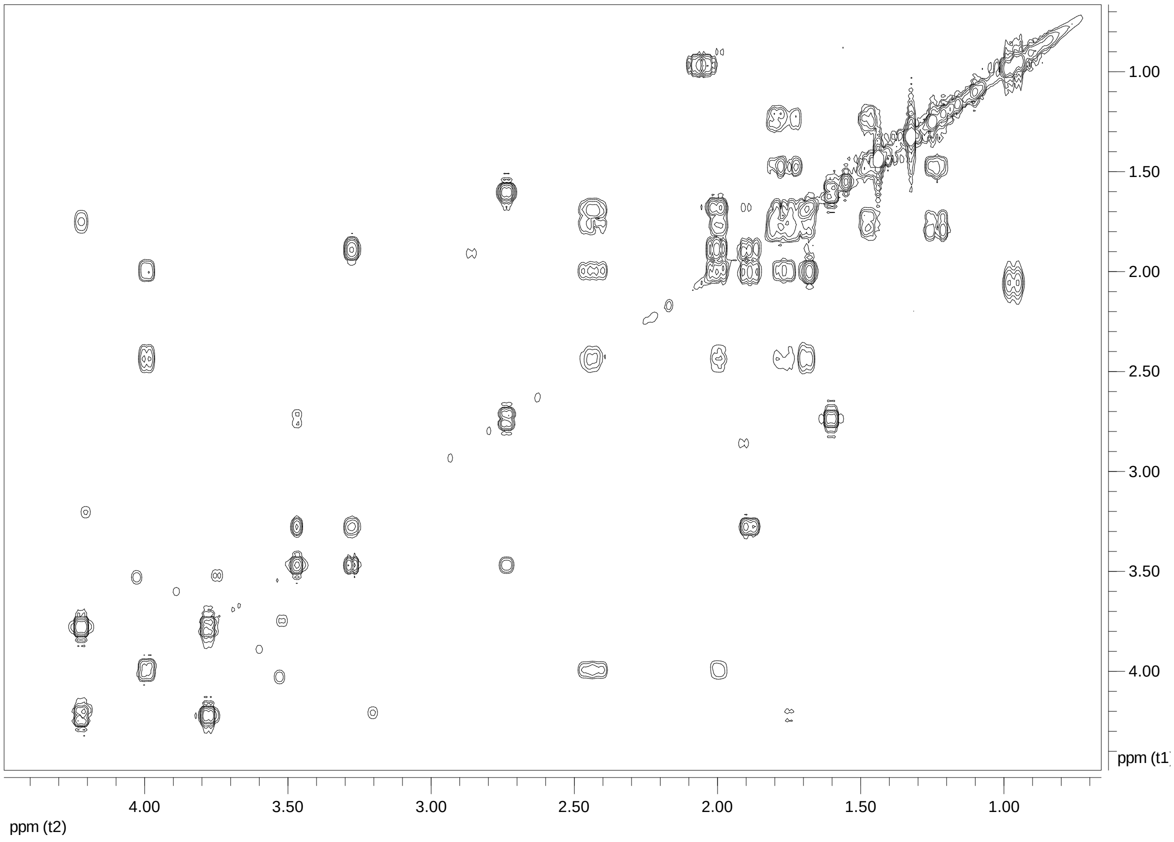

Figure S6. COSY spectrum ($CDCl_3$, 600 MHz) of compound **2**.

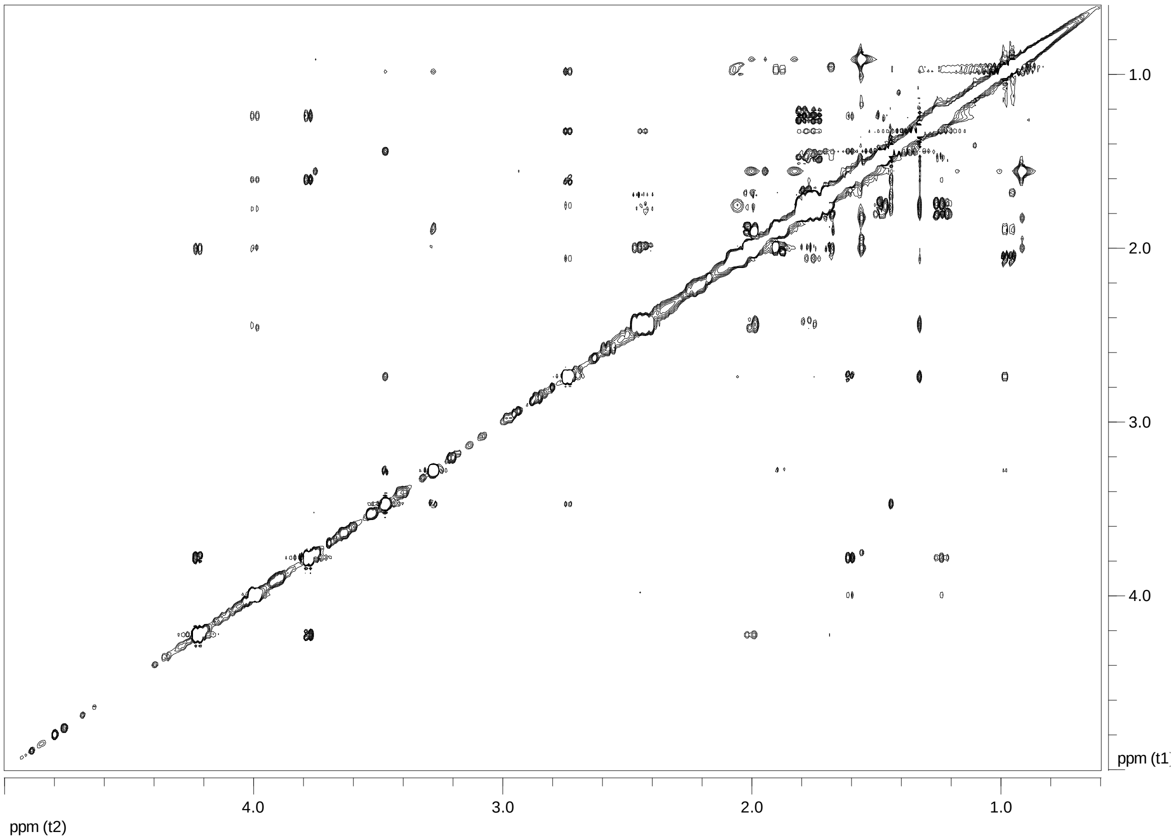

Figure S7. NOESY spectrum ($CDCl_3$, 600 MHz) of compound **2**.

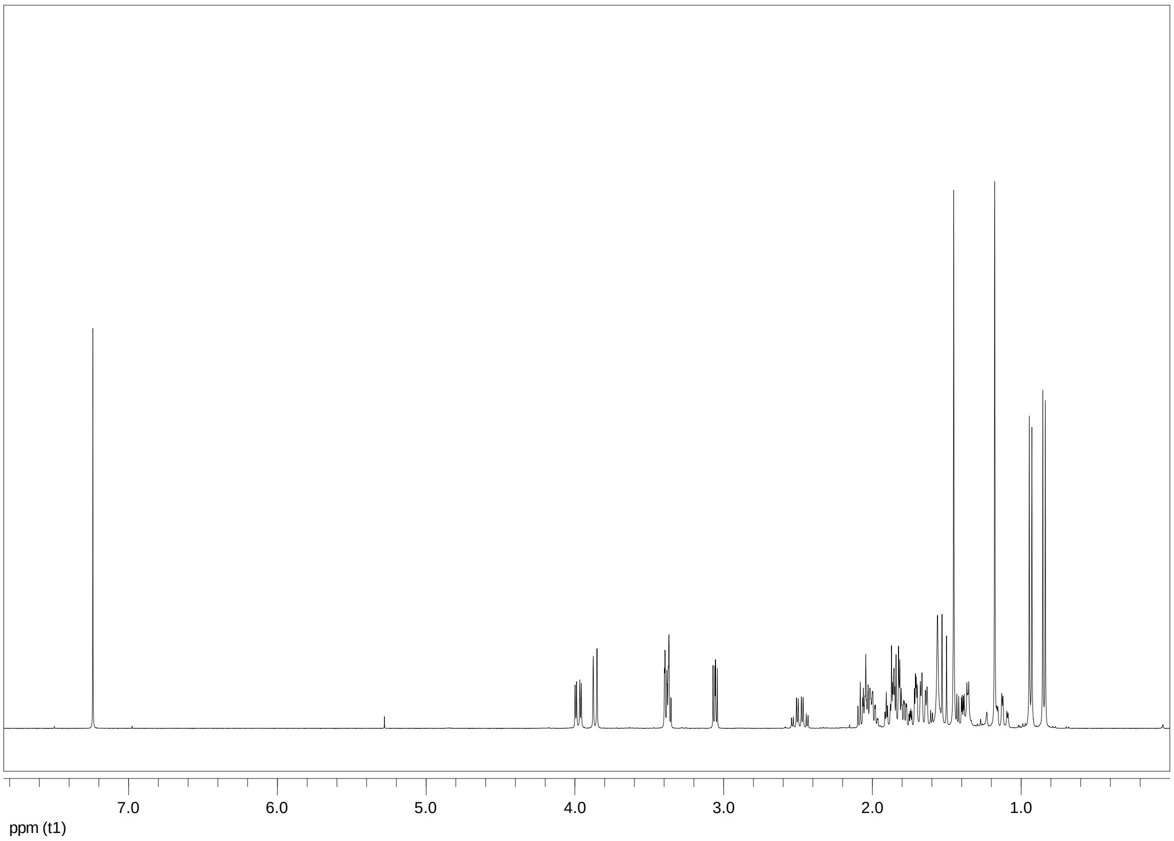

Figure S8. ^1H NMR spectrum ($CDCl_3$, 400 MHz) of compound **3**.

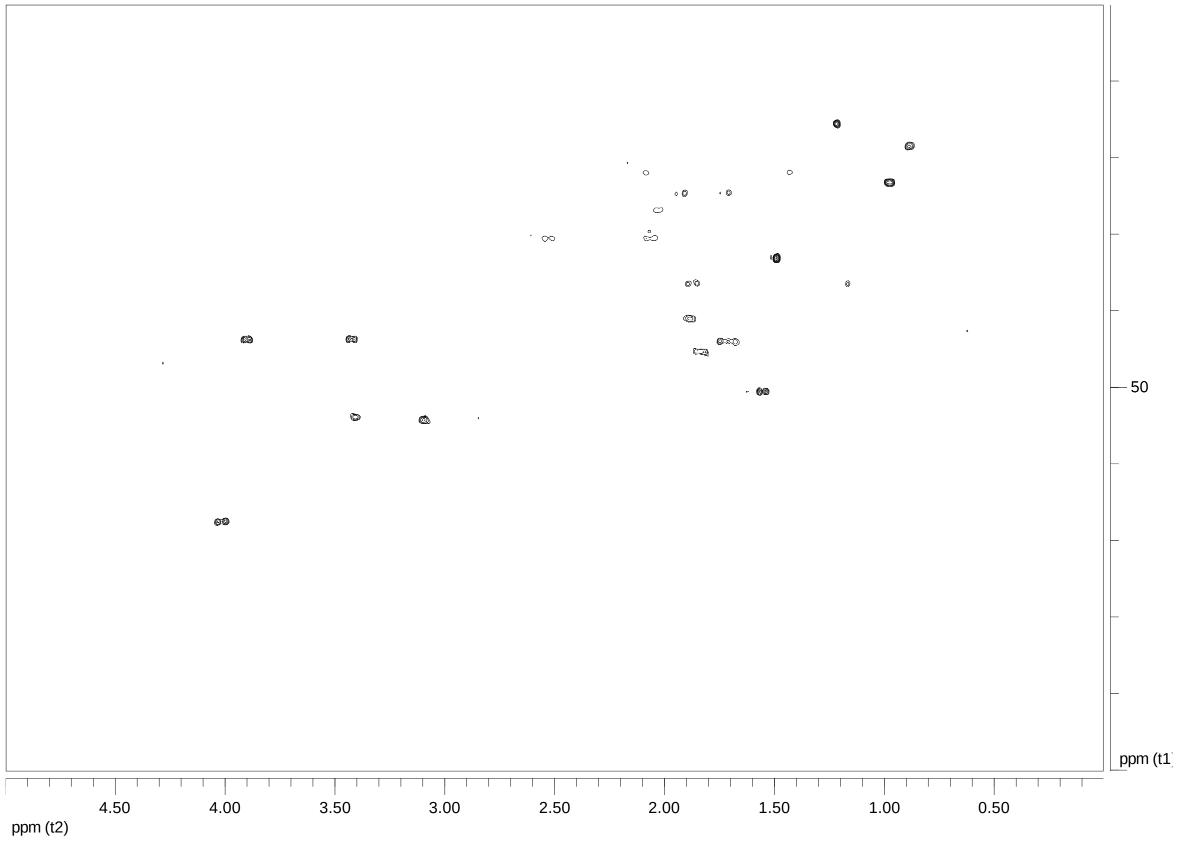

Figure S9. HSQC spectrum ($CDCl_3$, 400 MHz) of compound **3**.

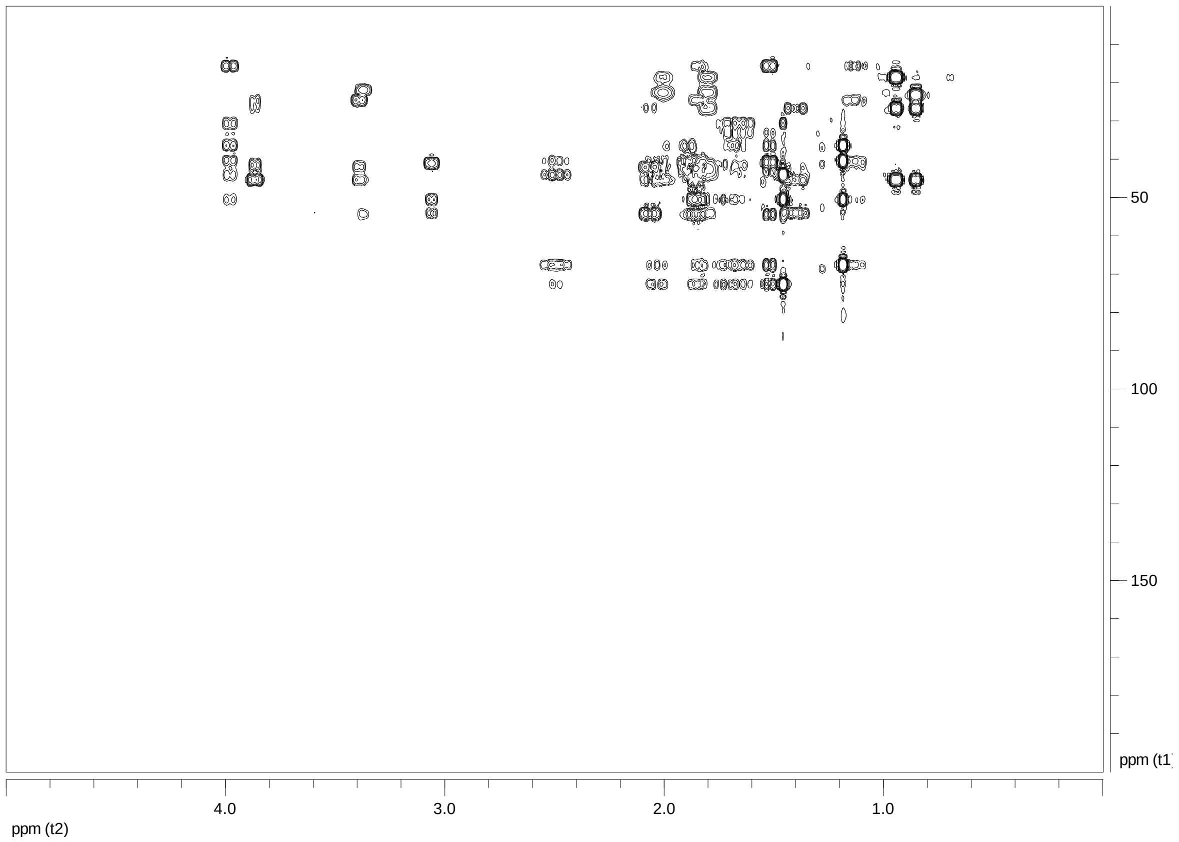

Figure S10. HMBC spectrum ($CDCl_3$, 400 MHz) of compound **3**.

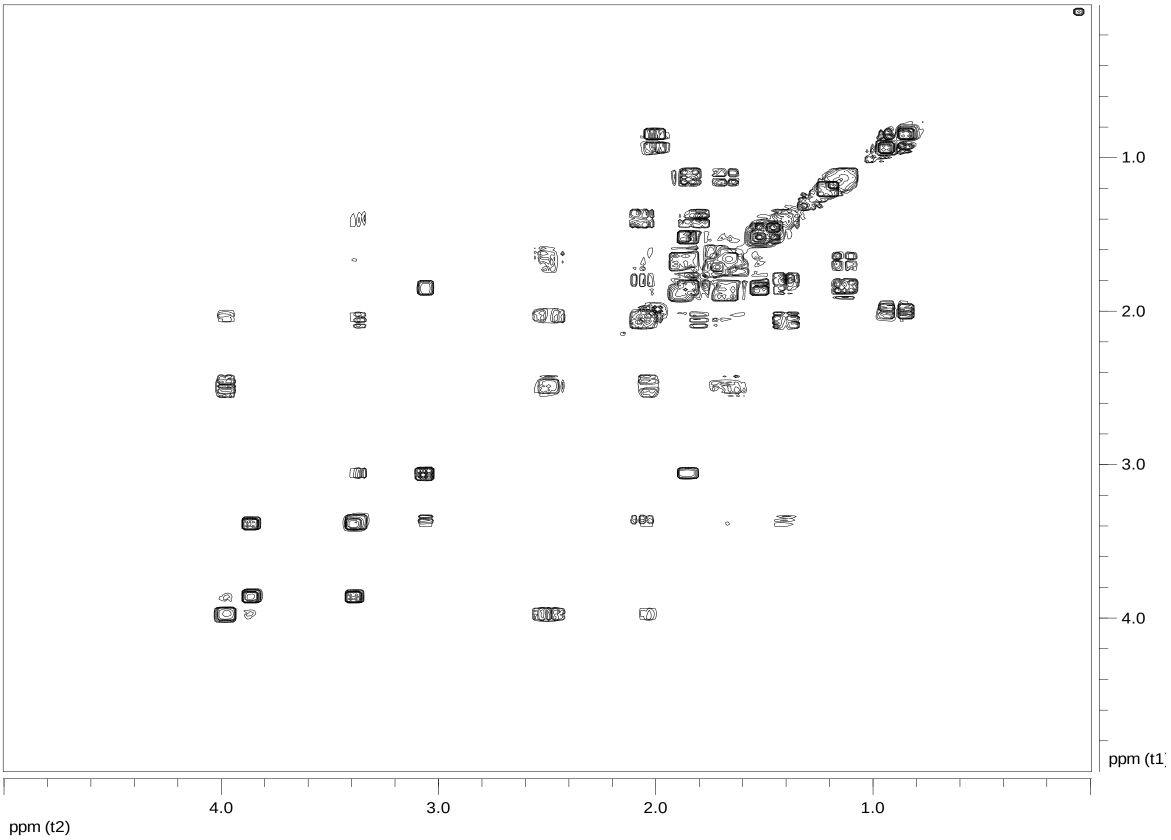

Figure S11. COSY spectrum ($CDCl_3$, 400 MHz) of compound **3**.

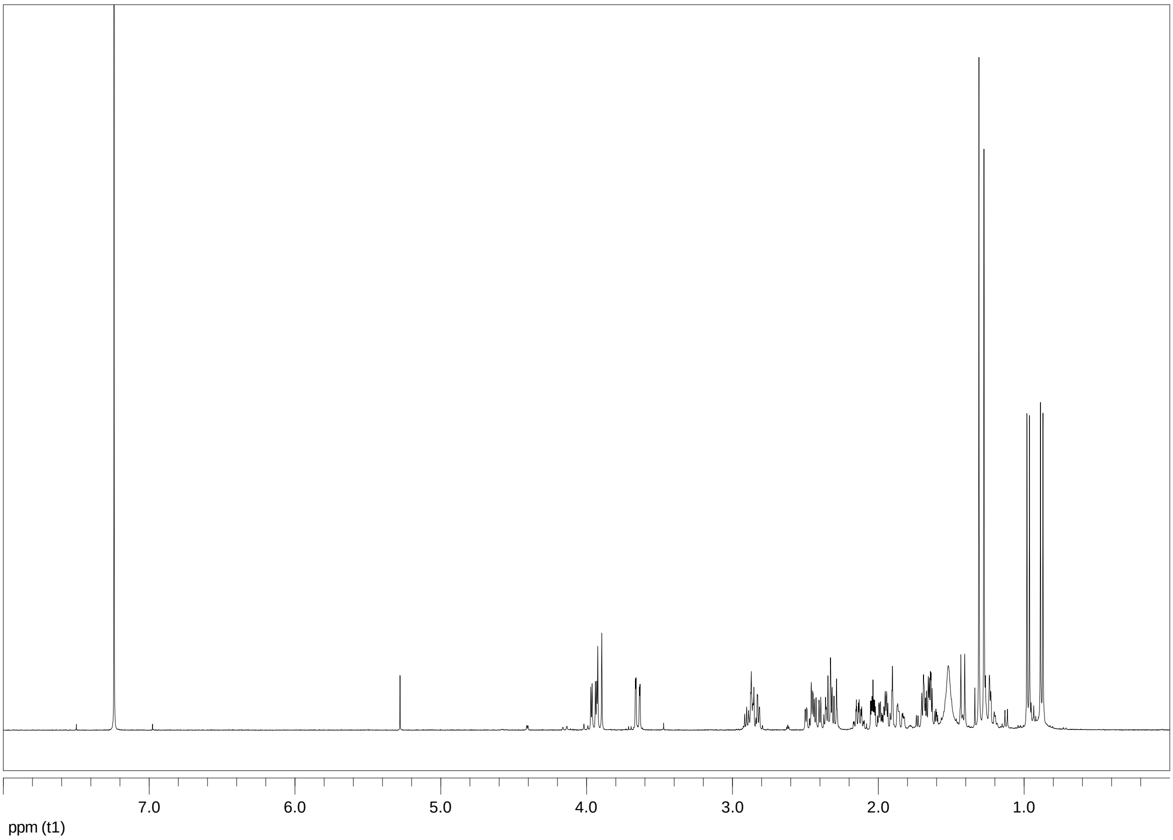

Figure S12. ^{1}H NMR spectrum ($CDCl_3$, 600 MHz) of compound **4**.

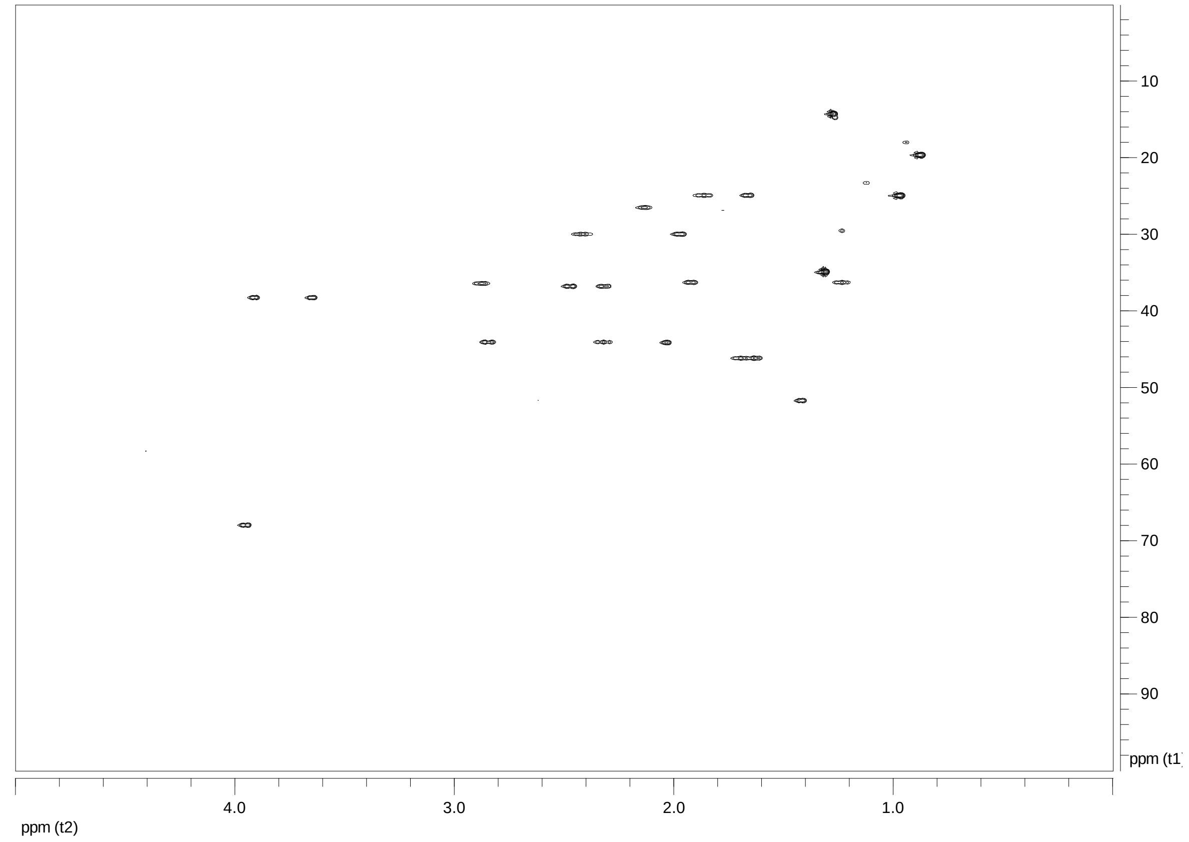

Figure S13. HSQC spectrum ($CDCl_3$, 600 MHz) of compound **4**.

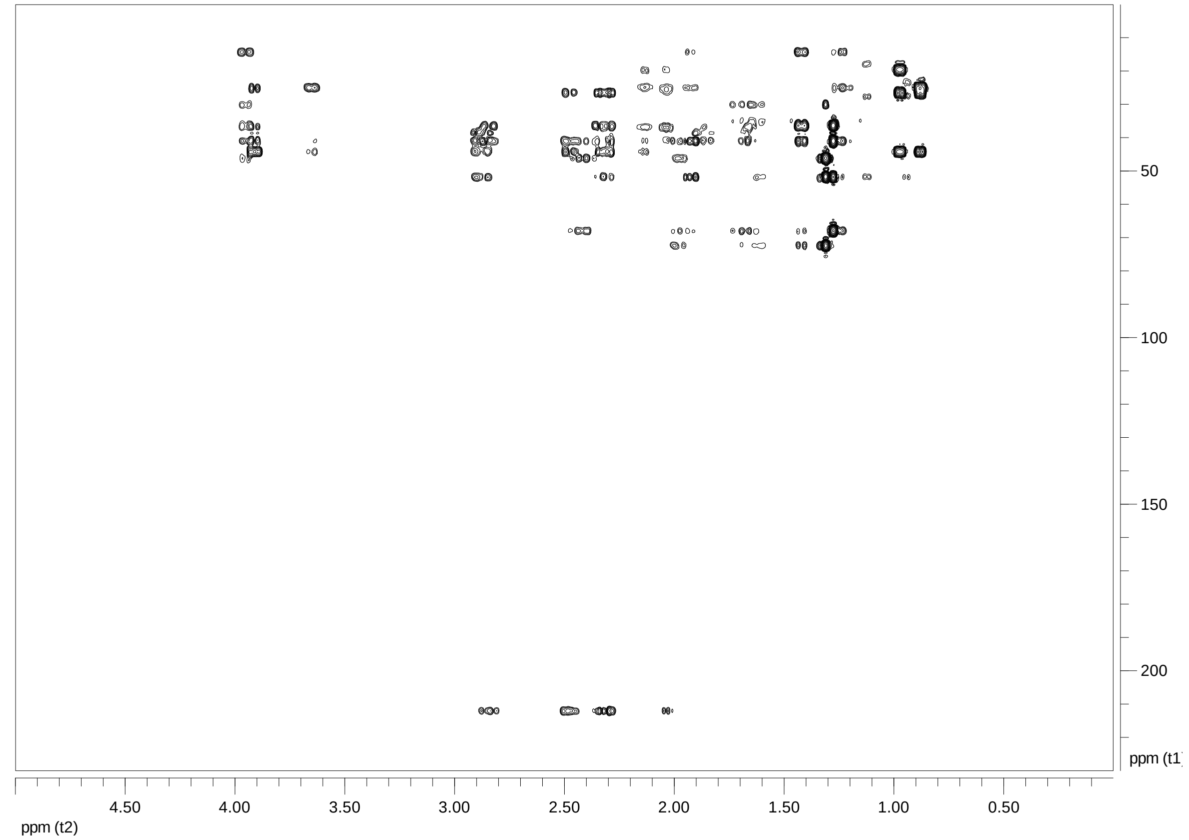

Figure S14. HMBC spectrum ($CDCl_3$, 400 MHz) of compound **4**.

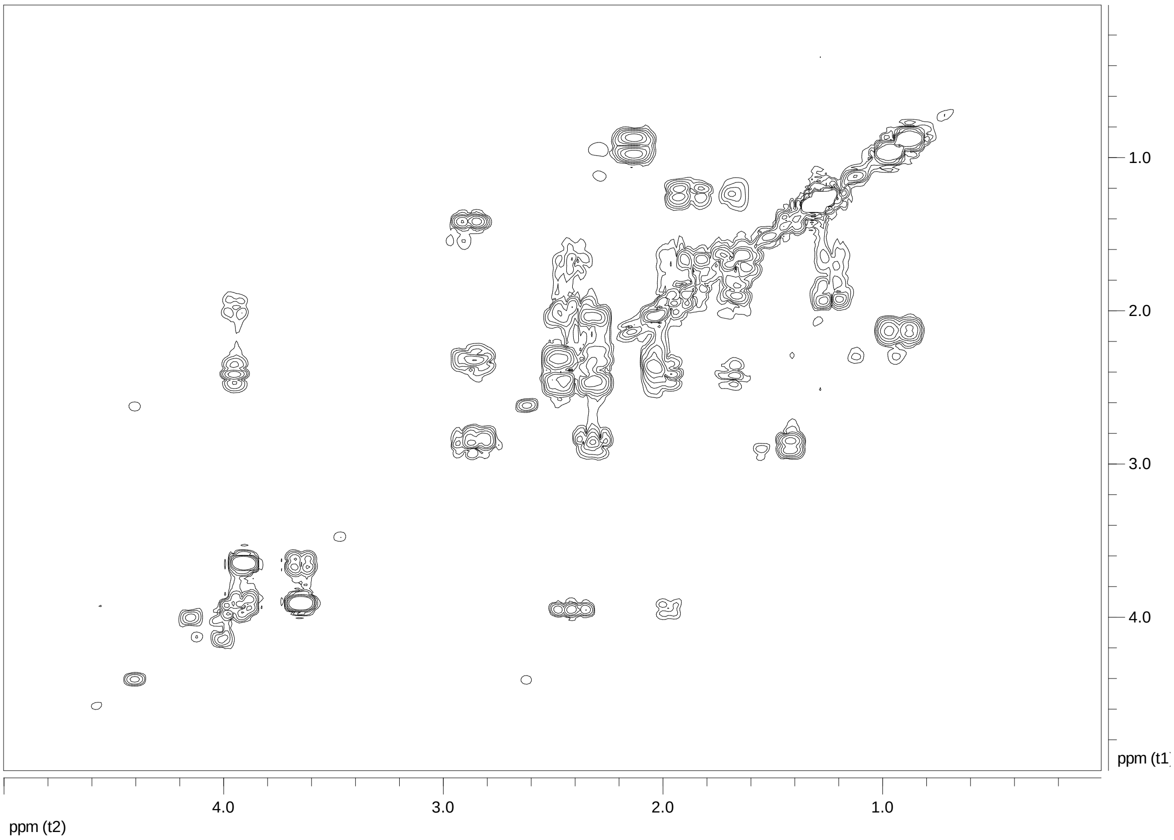

Figure S15. COSY spectrum ($CDCl_3$, 400 MHz) of compound **4**.

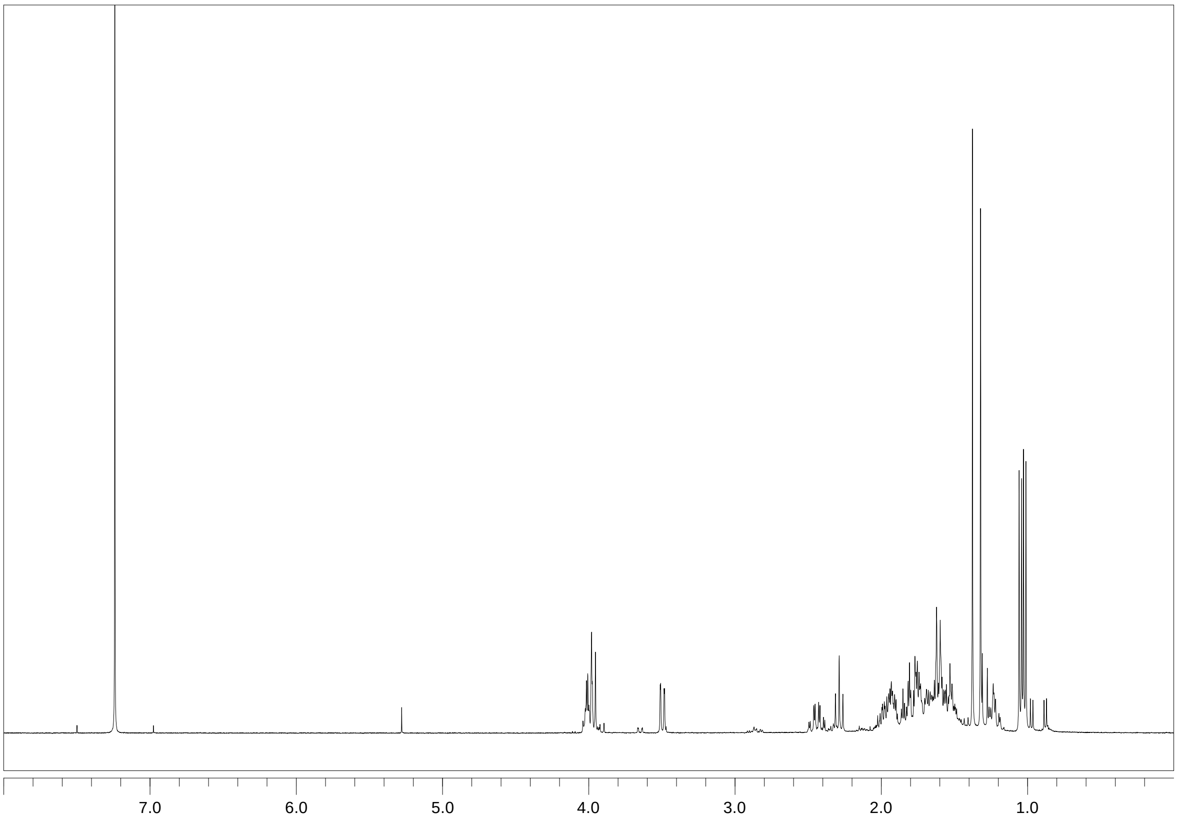

Figure S16. ^{1}H NMR spectrum ($CDCl_3$, 600 MHz) of compound **5**.

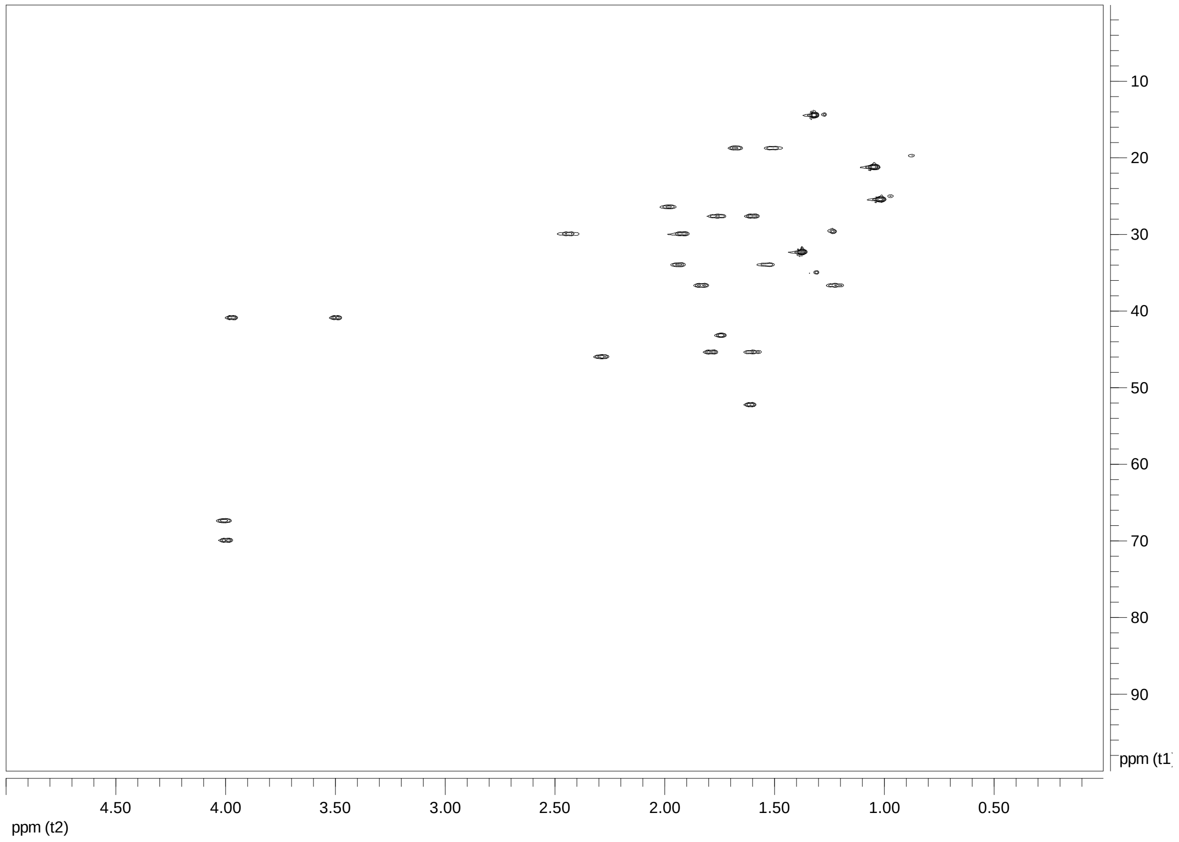

Figure S17. HSQC spectrum ($CDCl_3$, 600 MHz) of compound **5**.

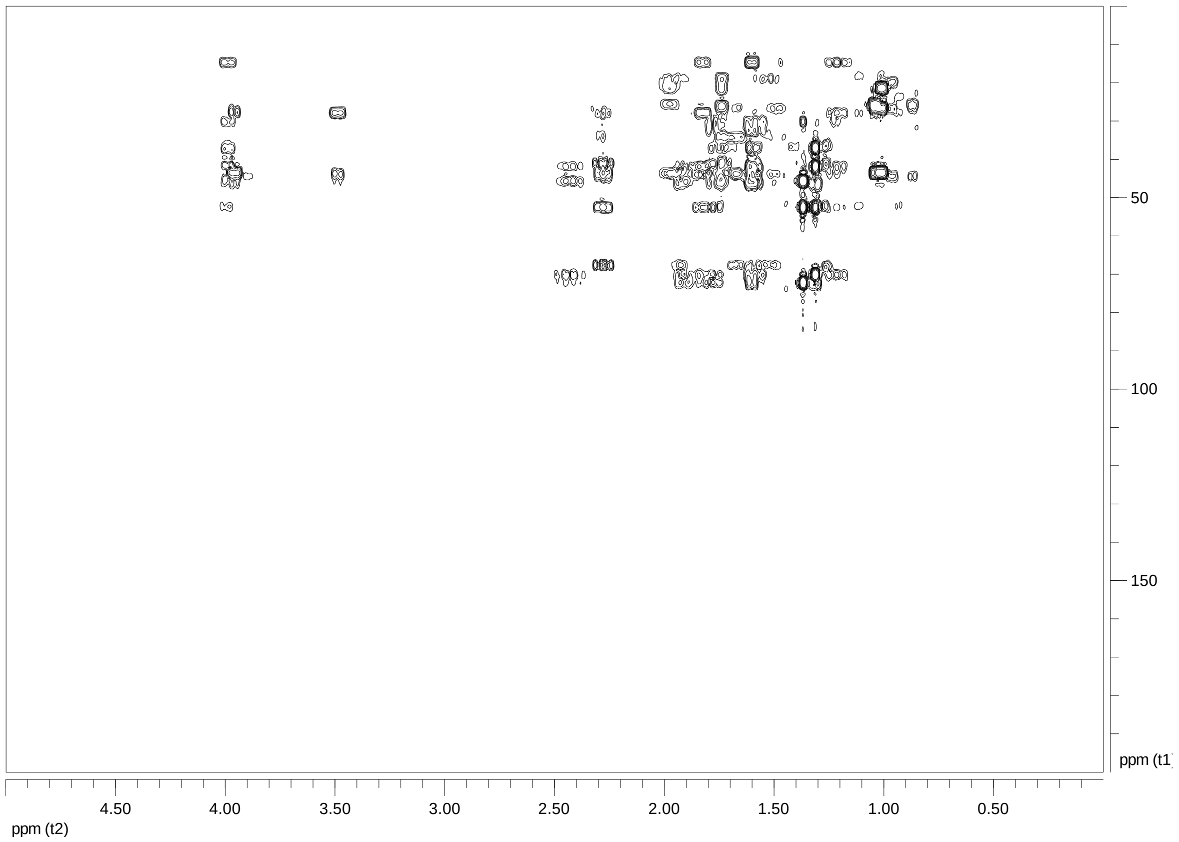

Figure S18. HMBC spectrum ($CDCl_3$, 400 MHz) of compound **5**.

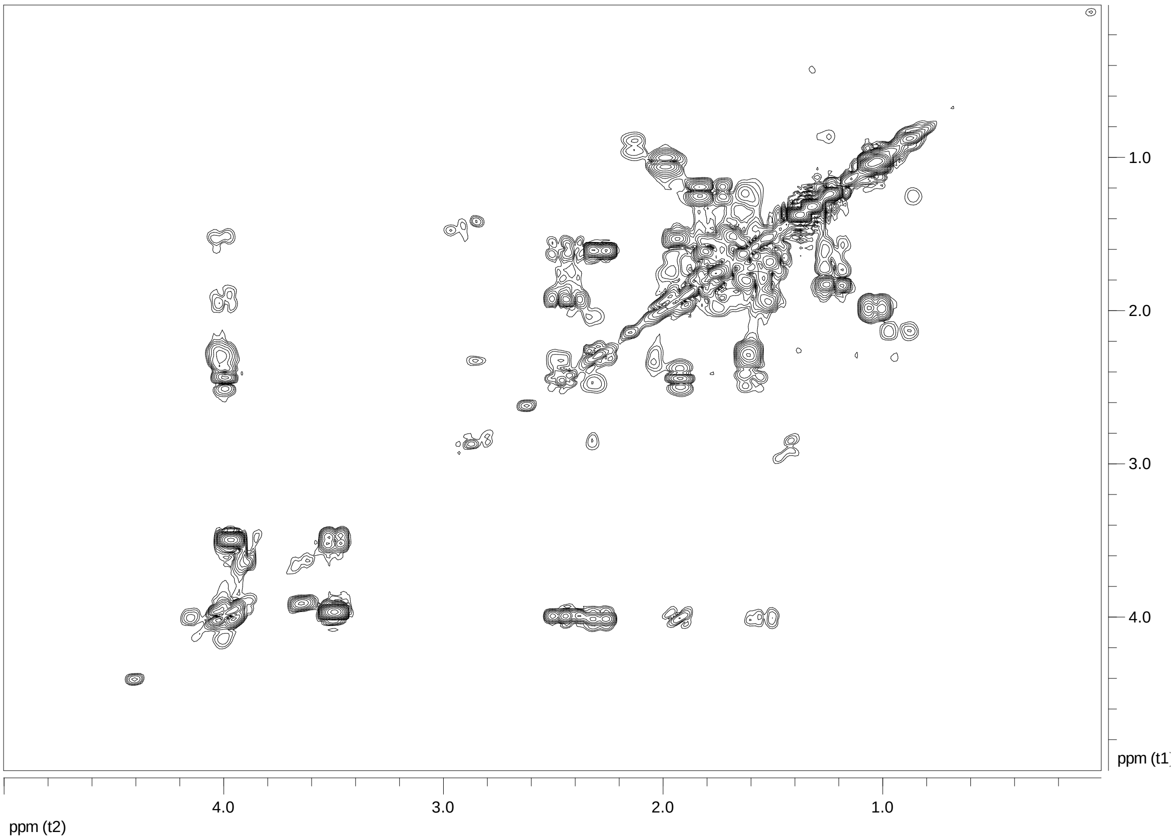

Figure S19. COSY spectrum ($CDCl_3$, 400 MHz) of compound **5**.

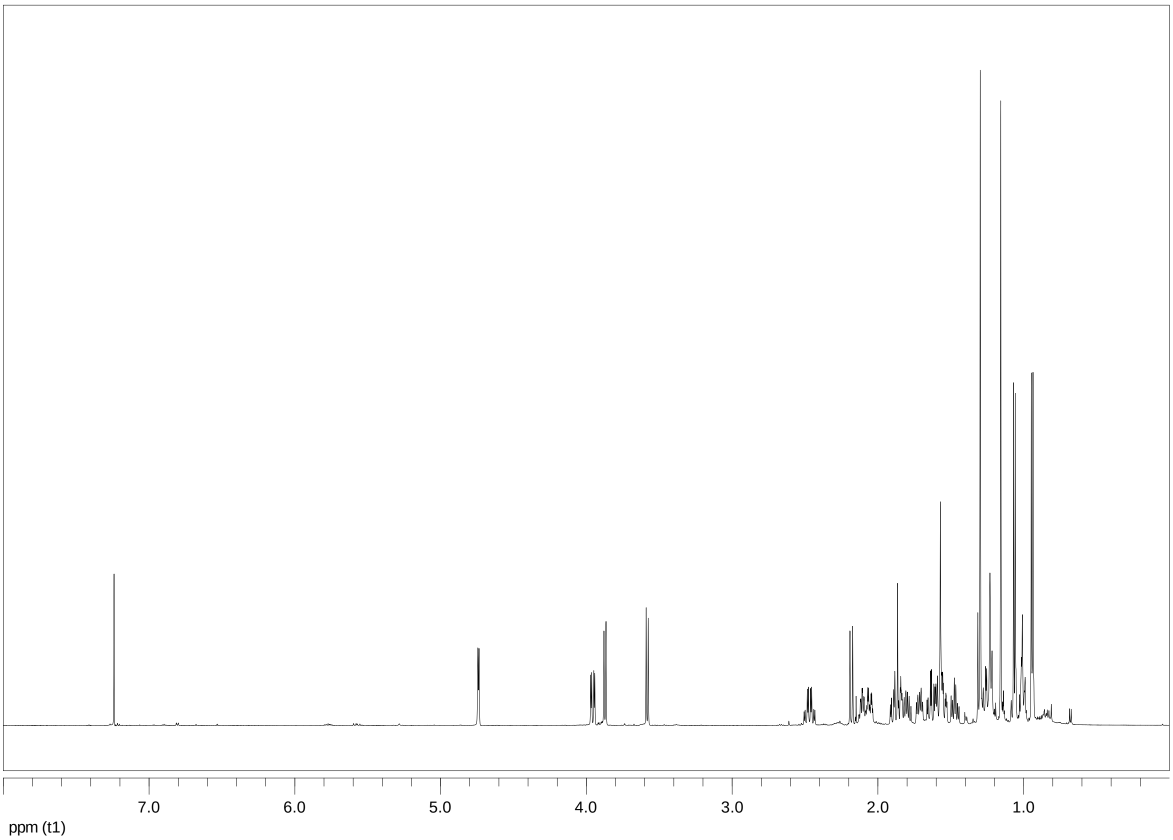

Figure S20. ^{1}H NMR spectrum ($CDCl_3$, 600 MHz) of compound **6**.

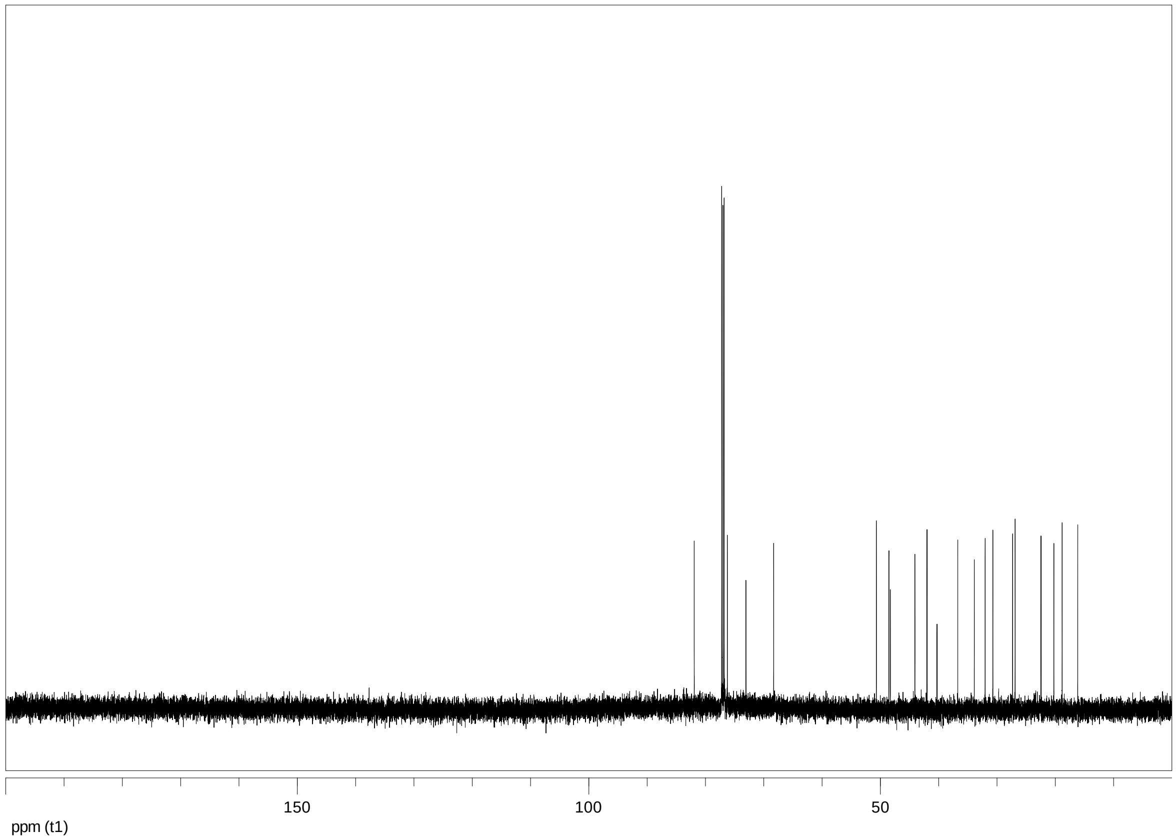

Figure S21. ^{13}C NMR spectrum ($CDCl_3$, 150 MHz) of compound **6**.

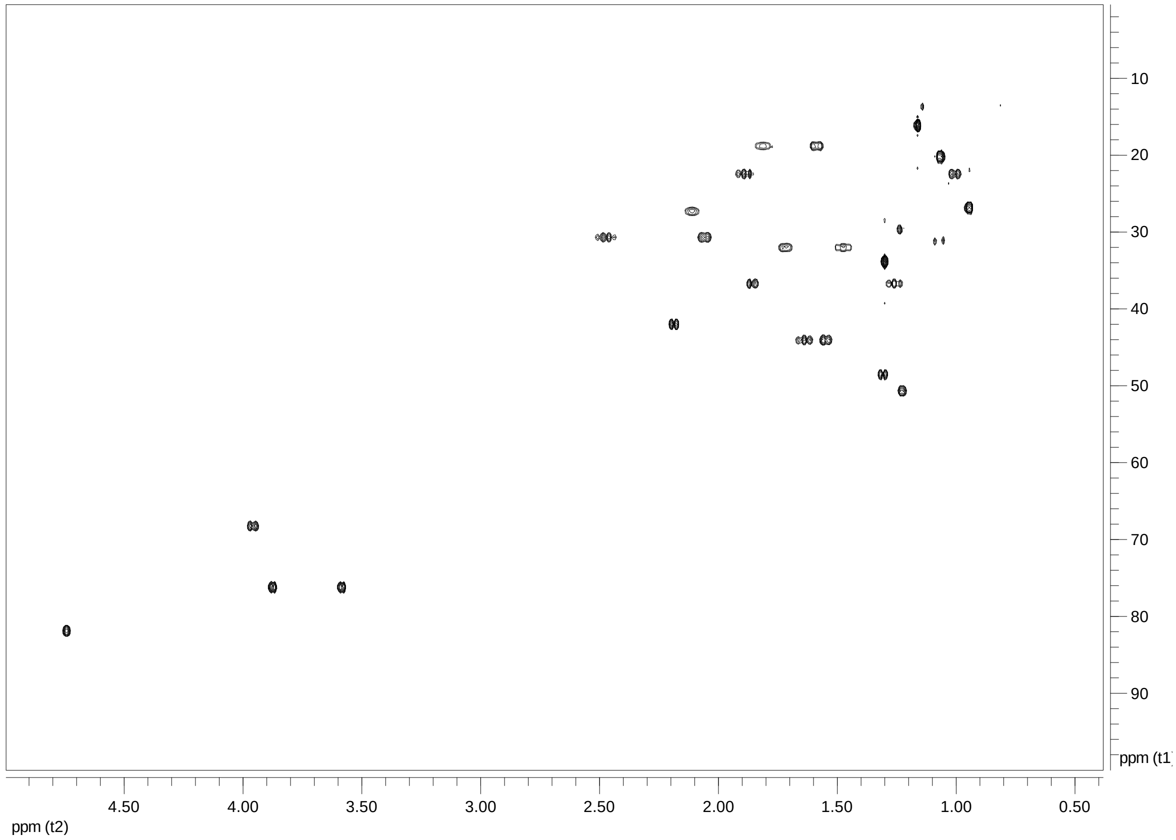

Figure S22. HSQC spectrum ($CDCl_3$, 600 MHz) of compound **6**.

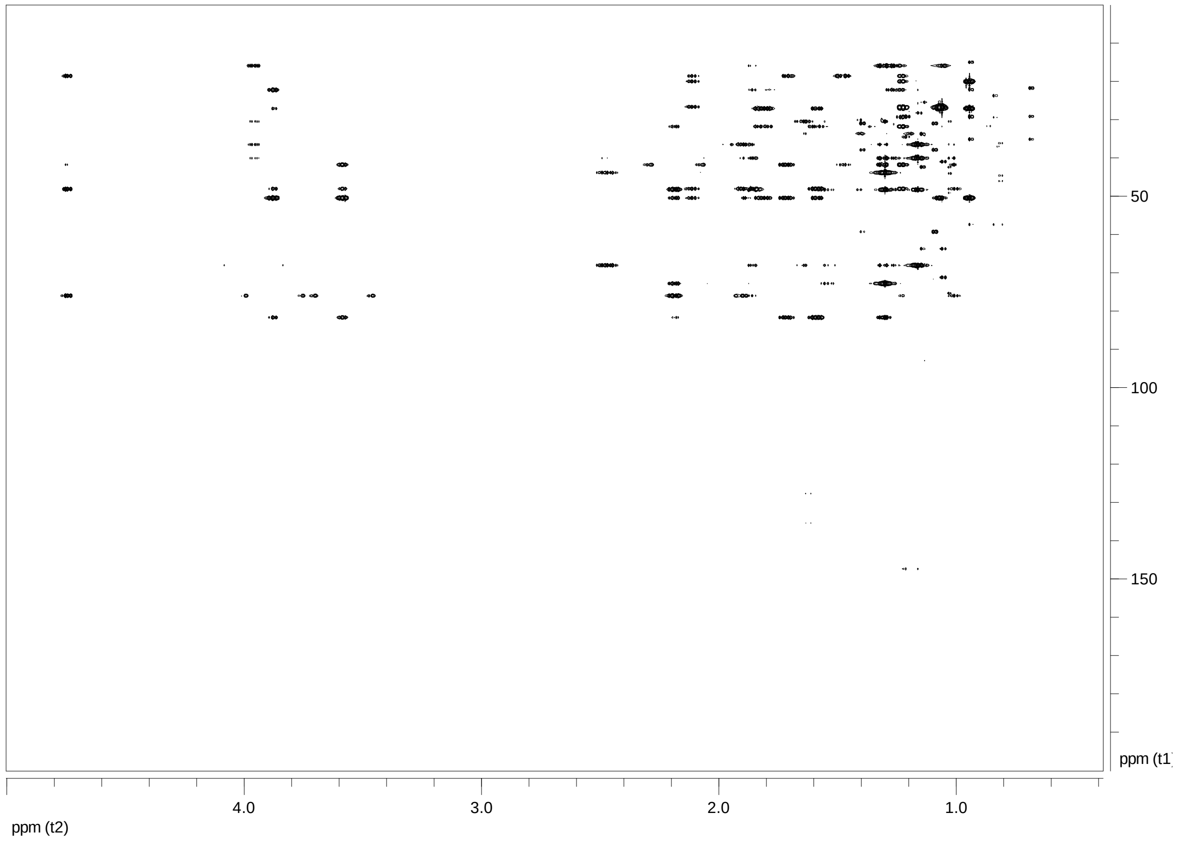

Figure S23. HMBC spectrum ($CDCl_3$, 600 MHz) of compound **6**.

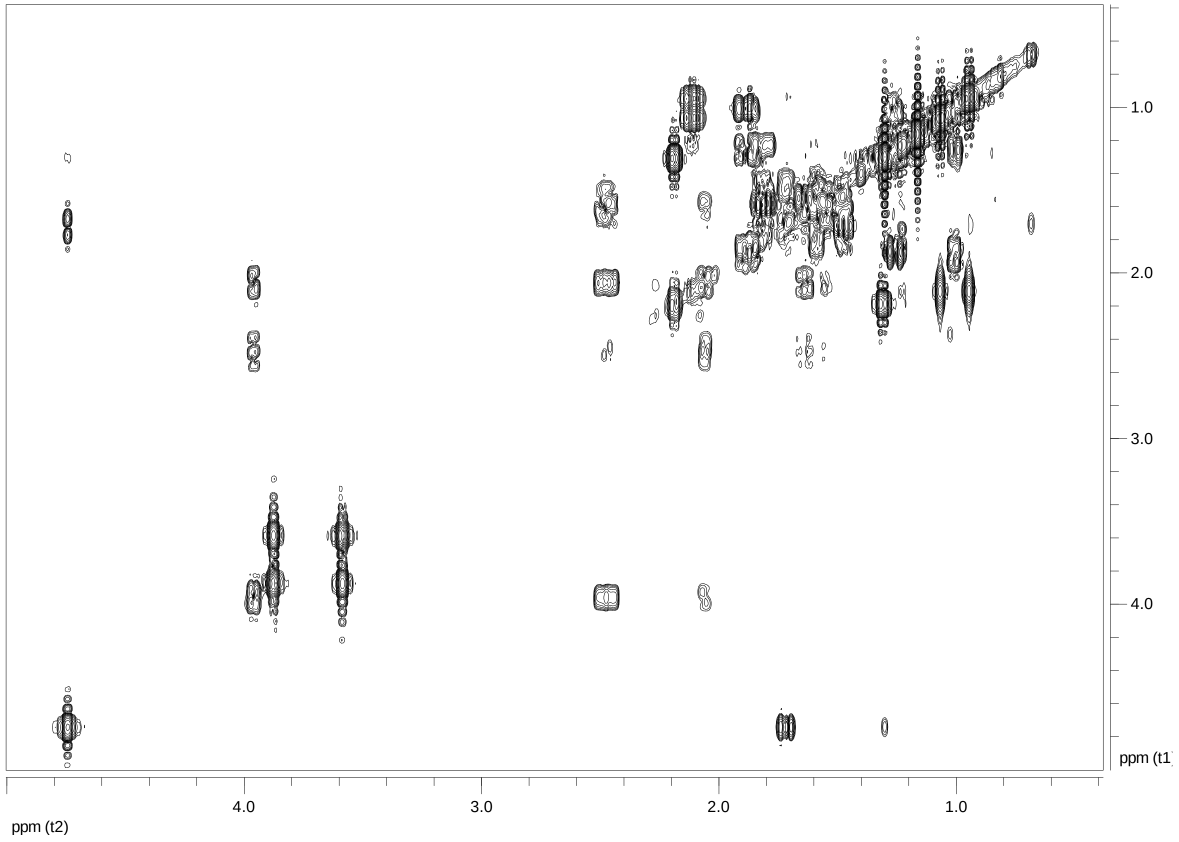

Figure S24. COSY spectrum ($CDCl_3$, 600 MHz) of compound **6**.

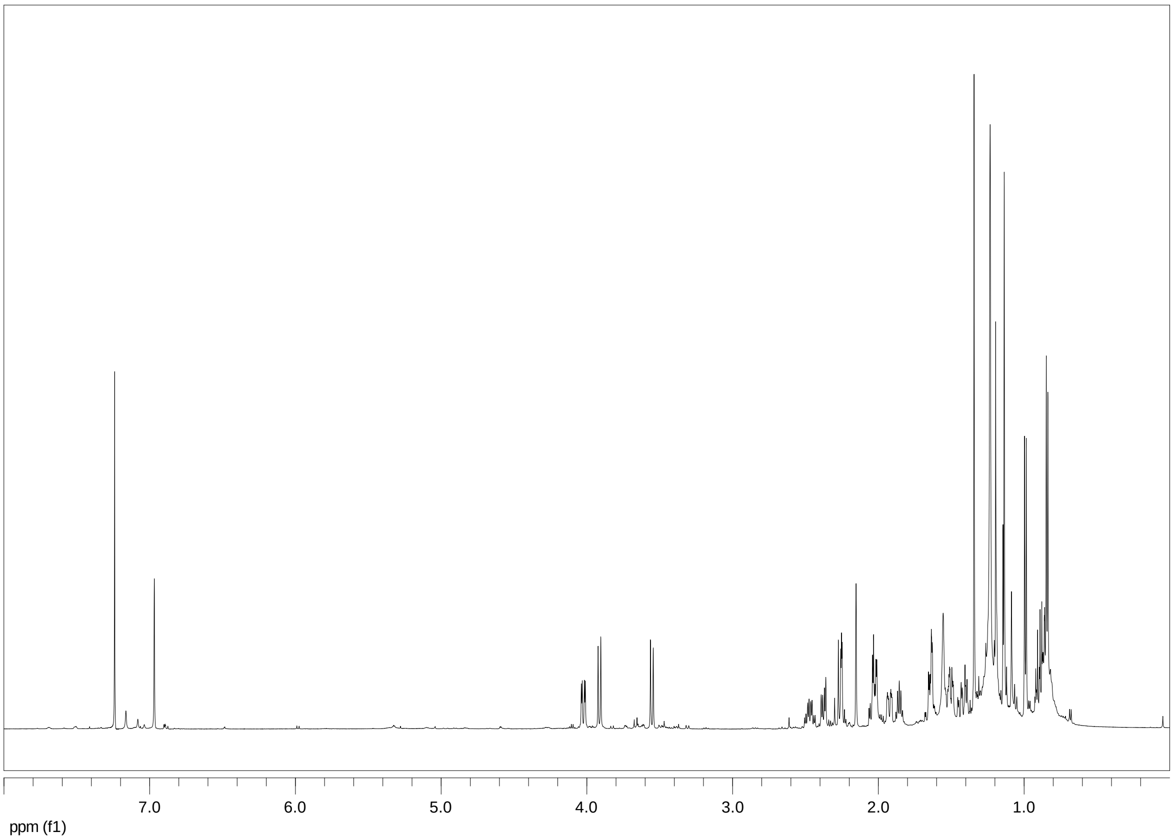

Figure S25. ^{1}H NMR spectrum ($CDCl_3$, 600 MHz) of compound **7**.

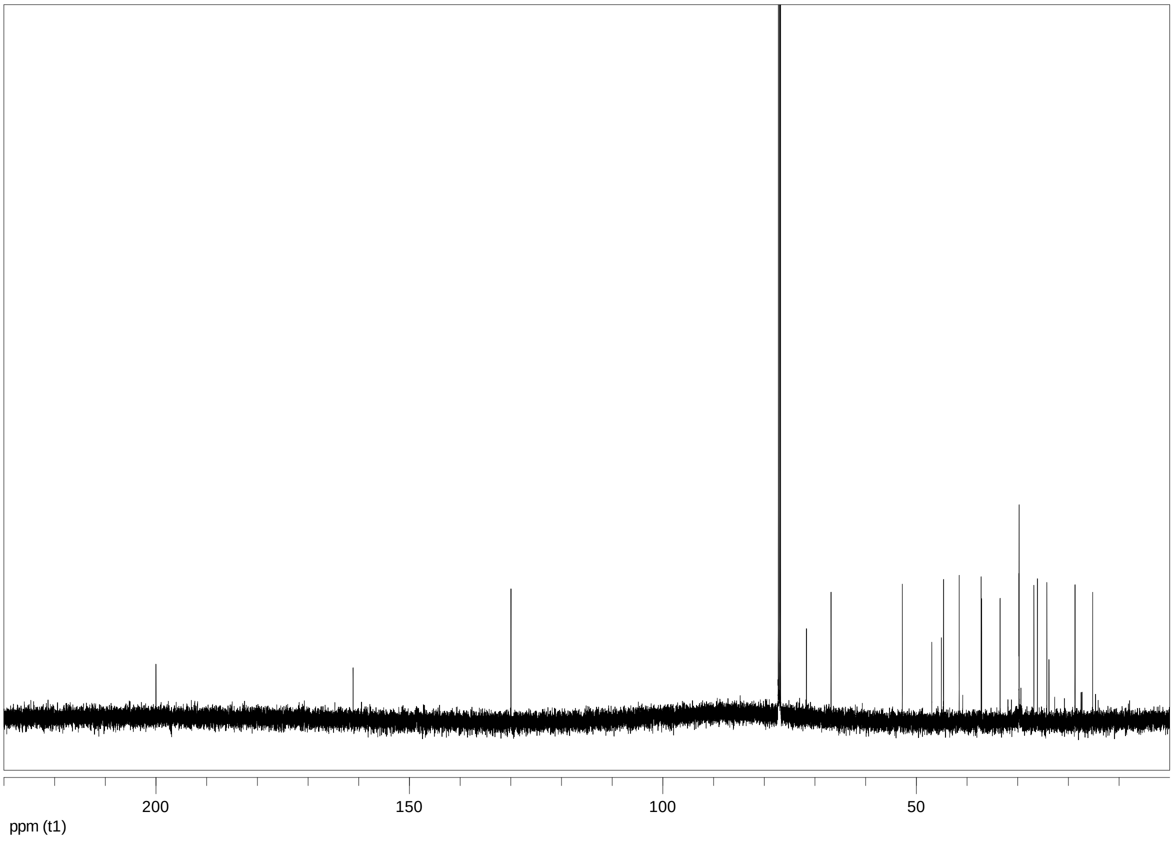

Figure S26. ^{13}C NMR spectrum ($CDCl_3$, 75 MHz) of compound **7**.

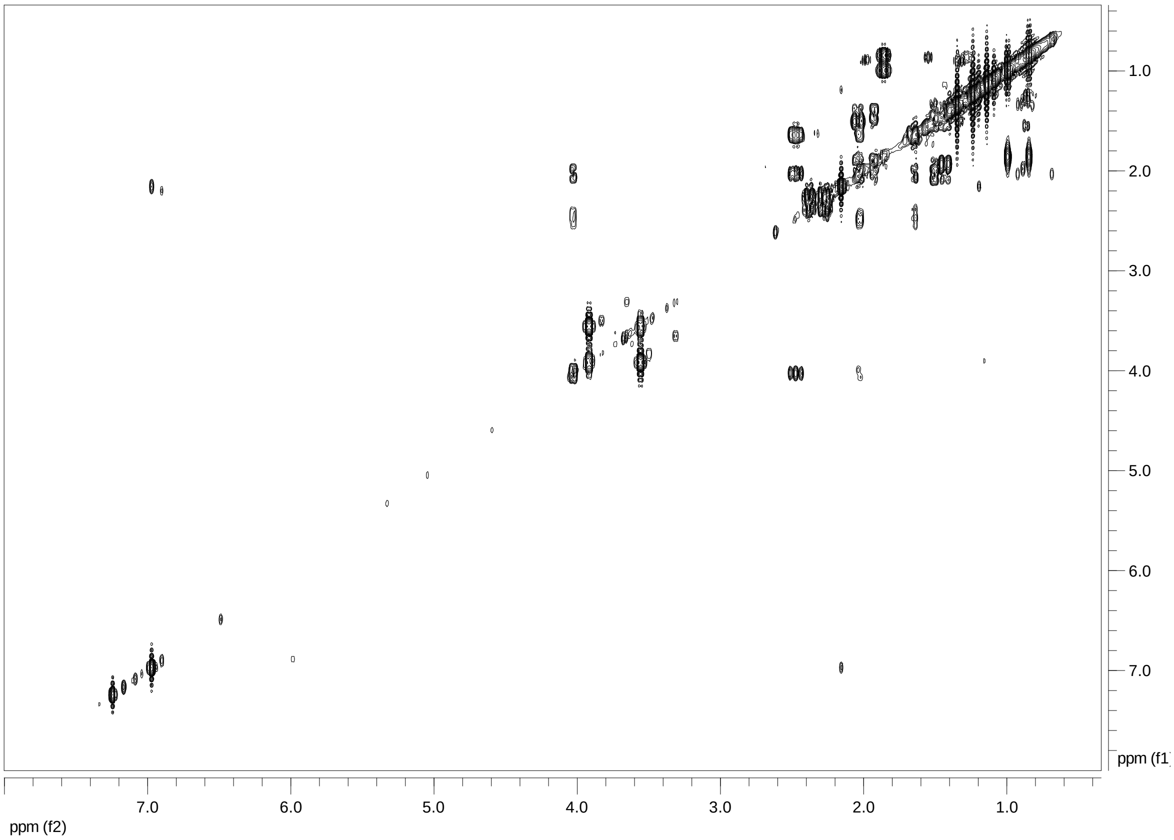

Figure S27. COSY spectrum ($CDCl_3$, 600 MHz) of compound **7**.

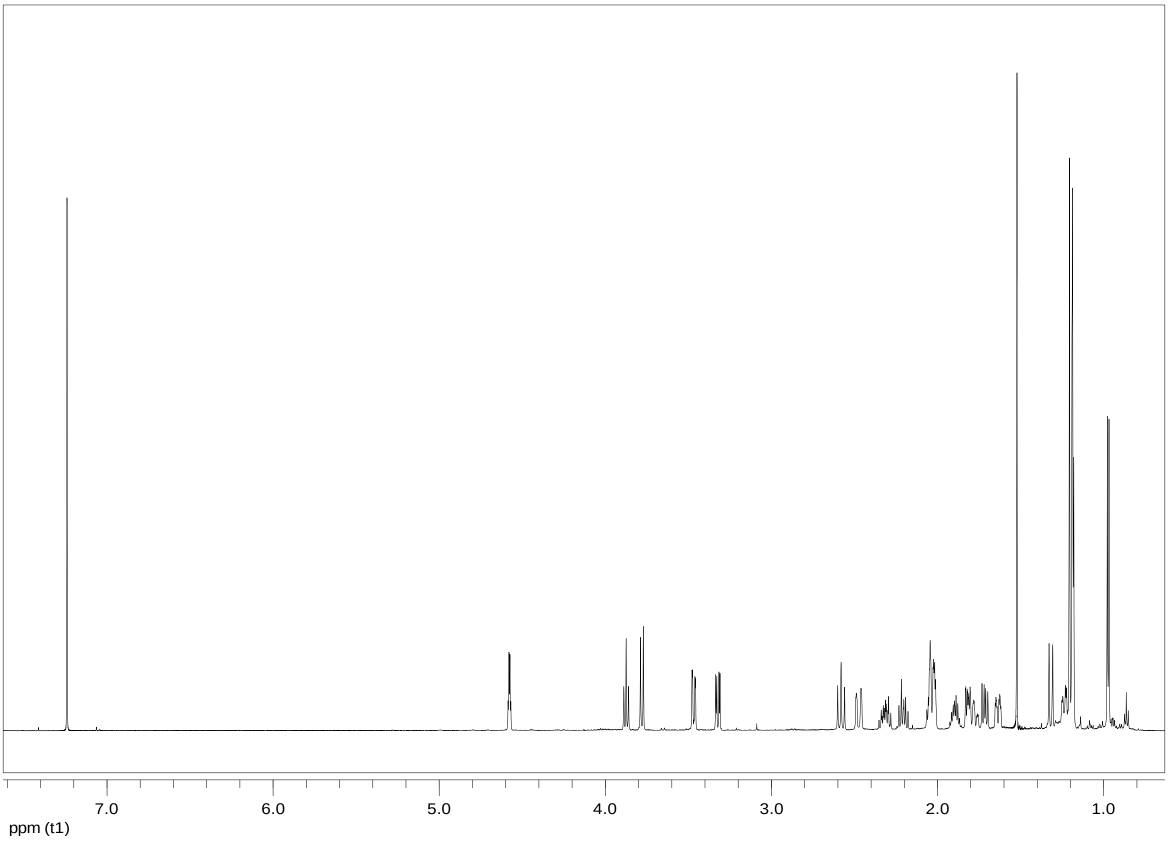

Figure S28. ^{1}H NMR spectrum ($CDCl_3$, 600 MHz) of compound **8**.

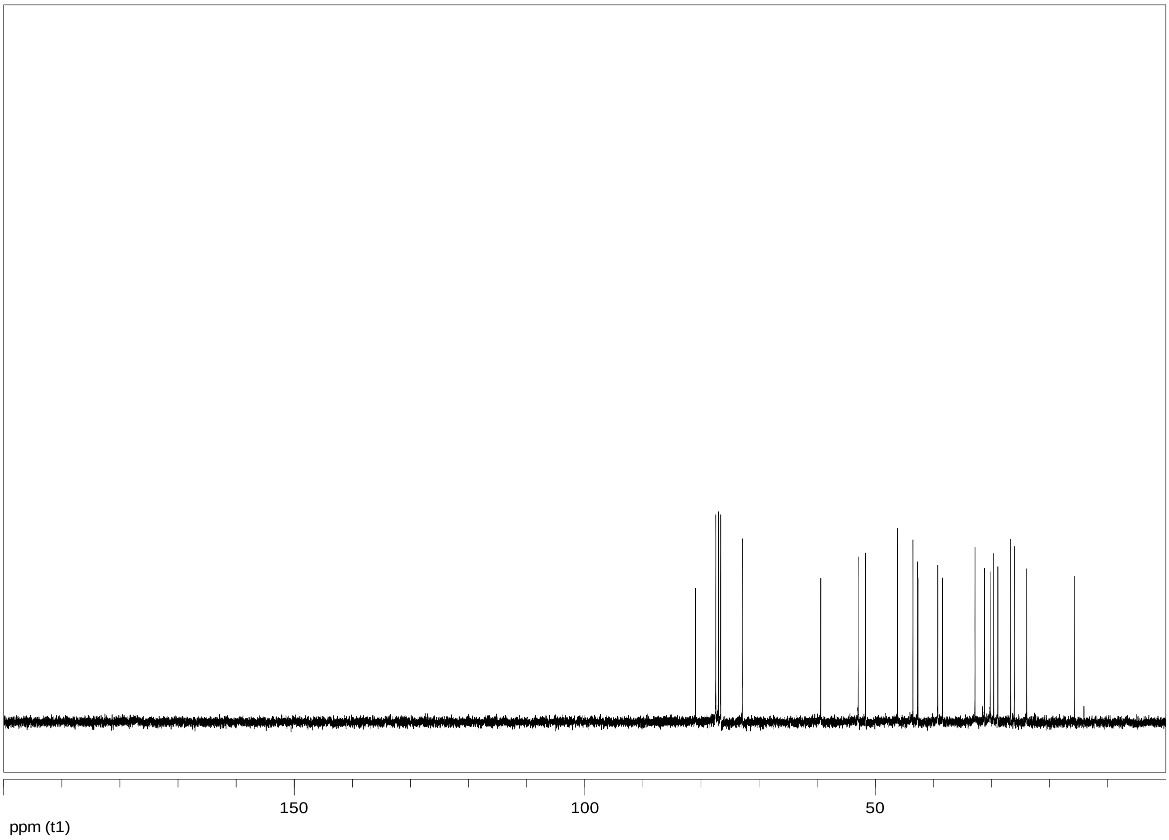

Figure S29. ^{13}C NMR spectrum ($CDCl_3$, 75 MHz) of compound **8**.

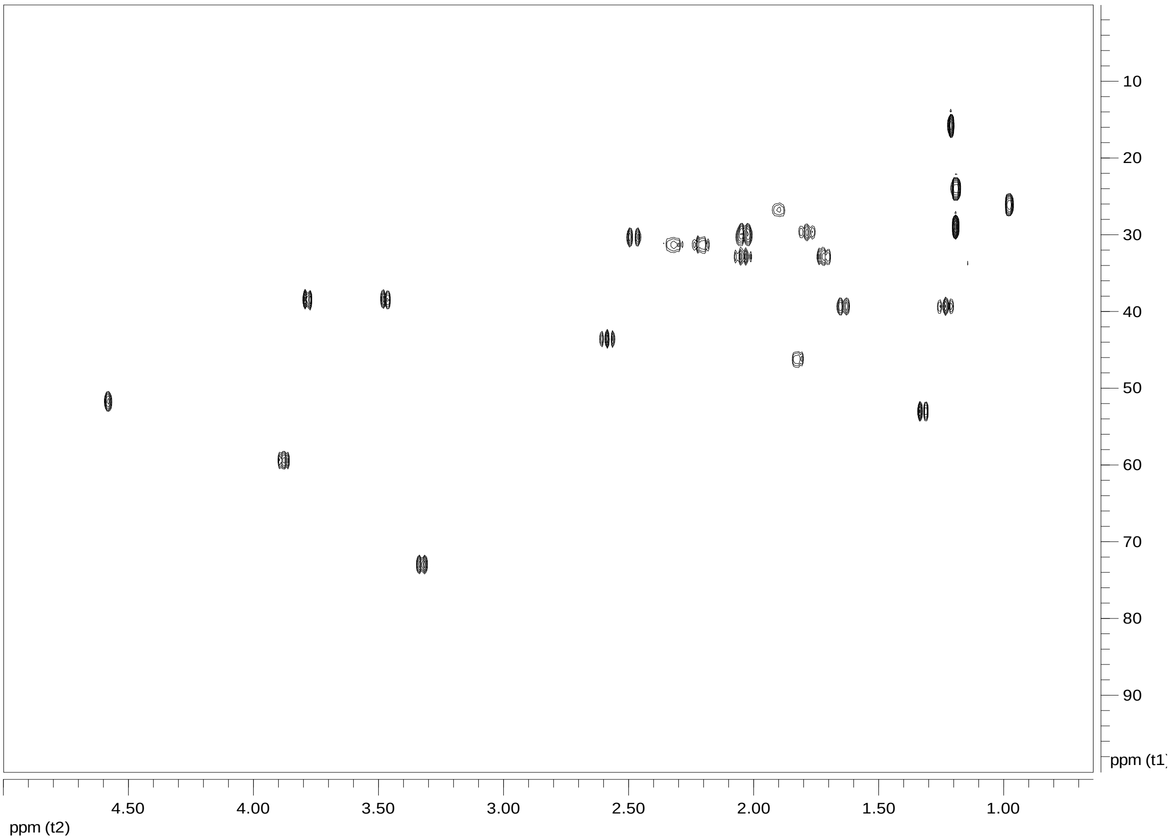

Figure S30. HSQC spectrum ($CDCl_3$, 600 MHz) of compound **8**.

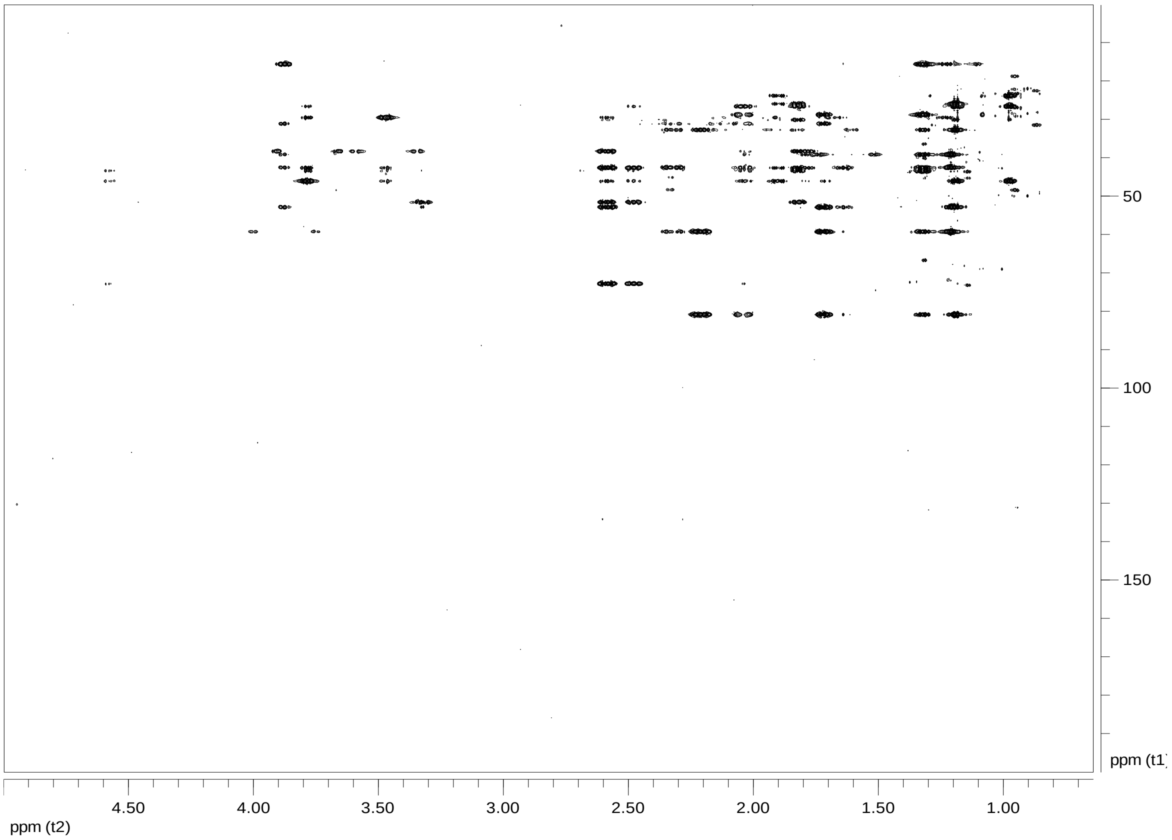

Figure S31. HMBC spectrum ($CDCl_3$, 600 MHz) of compound **8**.

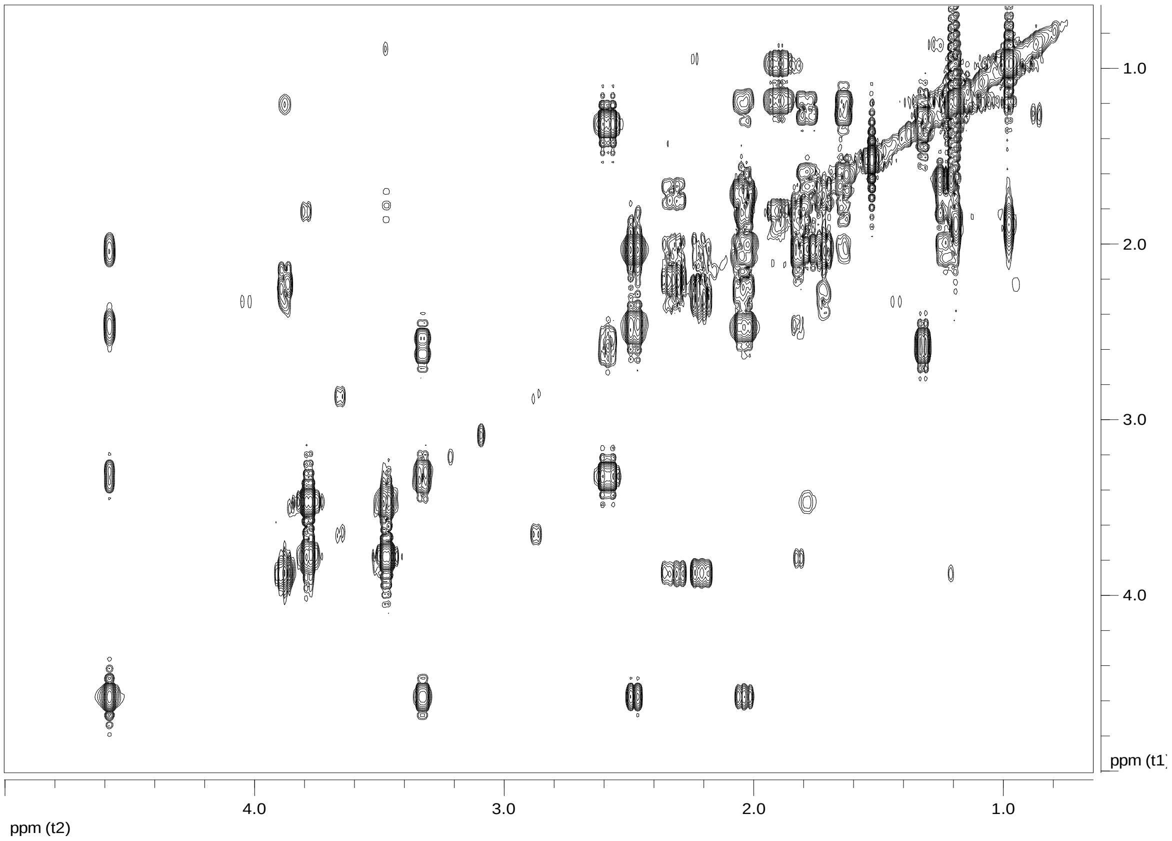

Figure S32. COSY spectrum ($CDCl_3$, 600 MHz) of compound **8**.

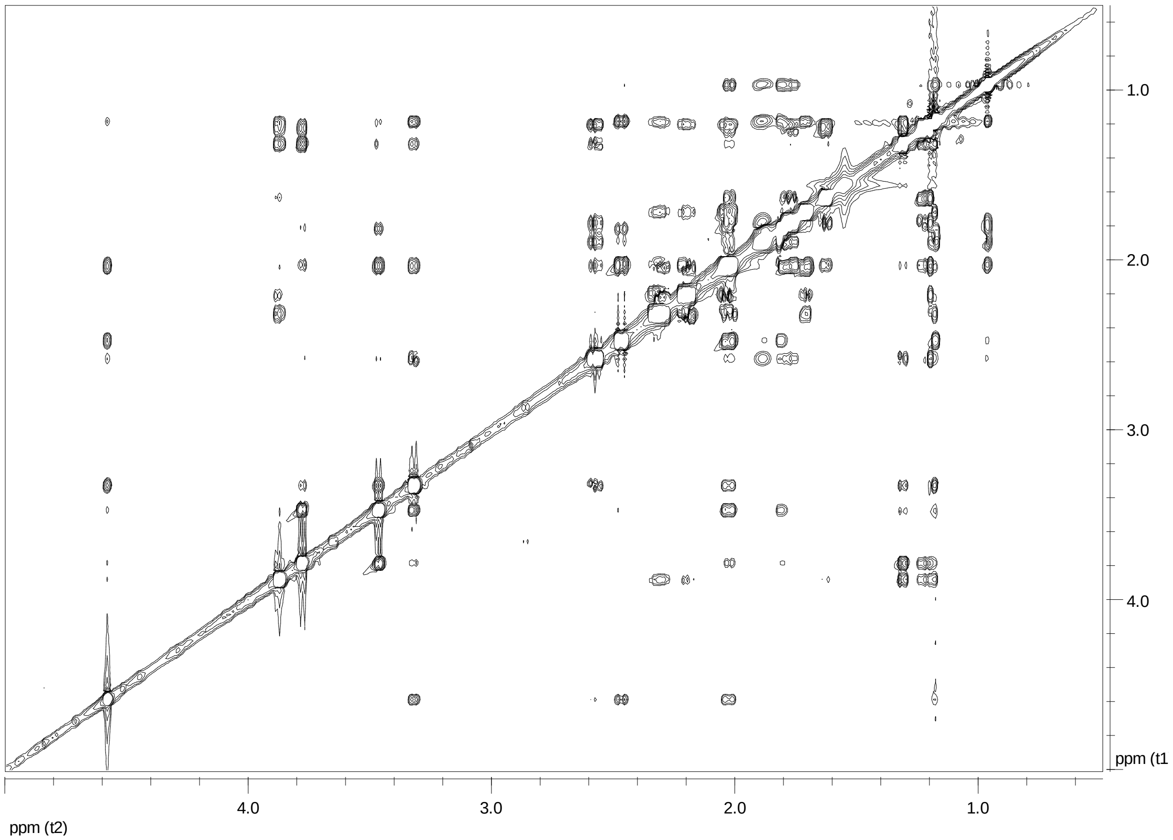

Figure S33. NOESY spectrum ($CDCl_3$, 600 MHz) of compound **8**.

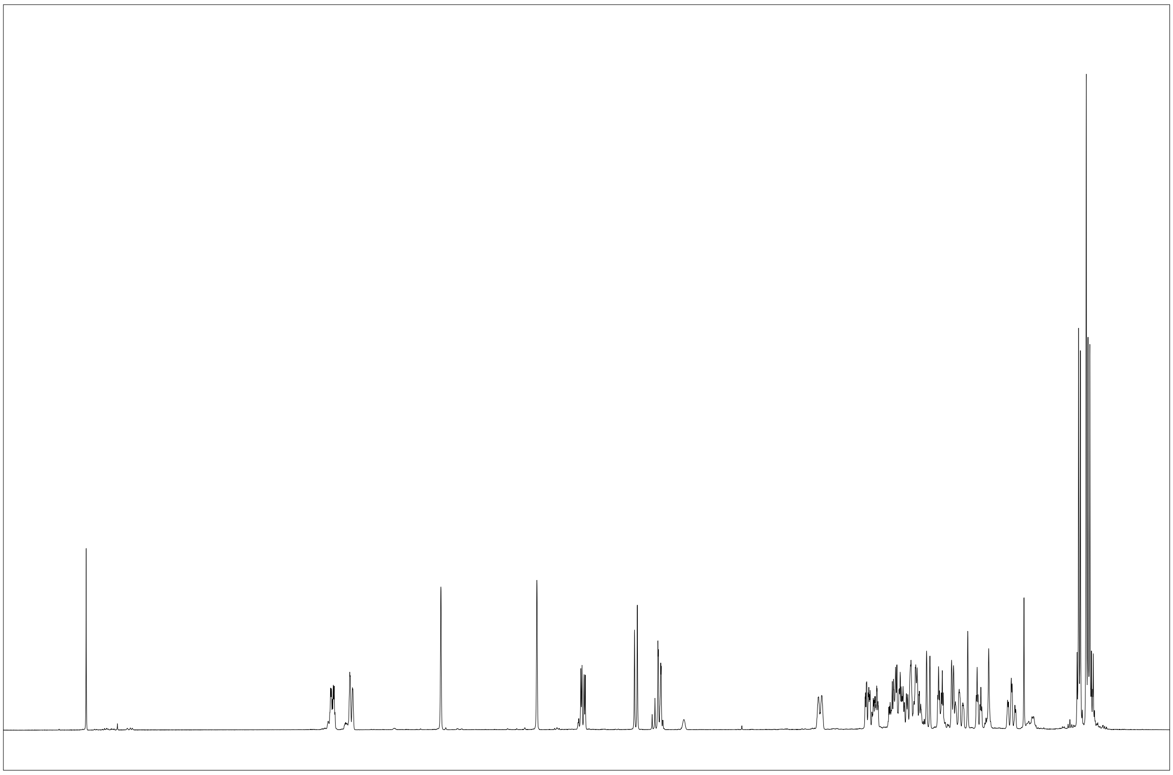

Figure S34. ^{1}H NMR spectrum ($CDCl_3$, 600 MHz) of compound **9**.

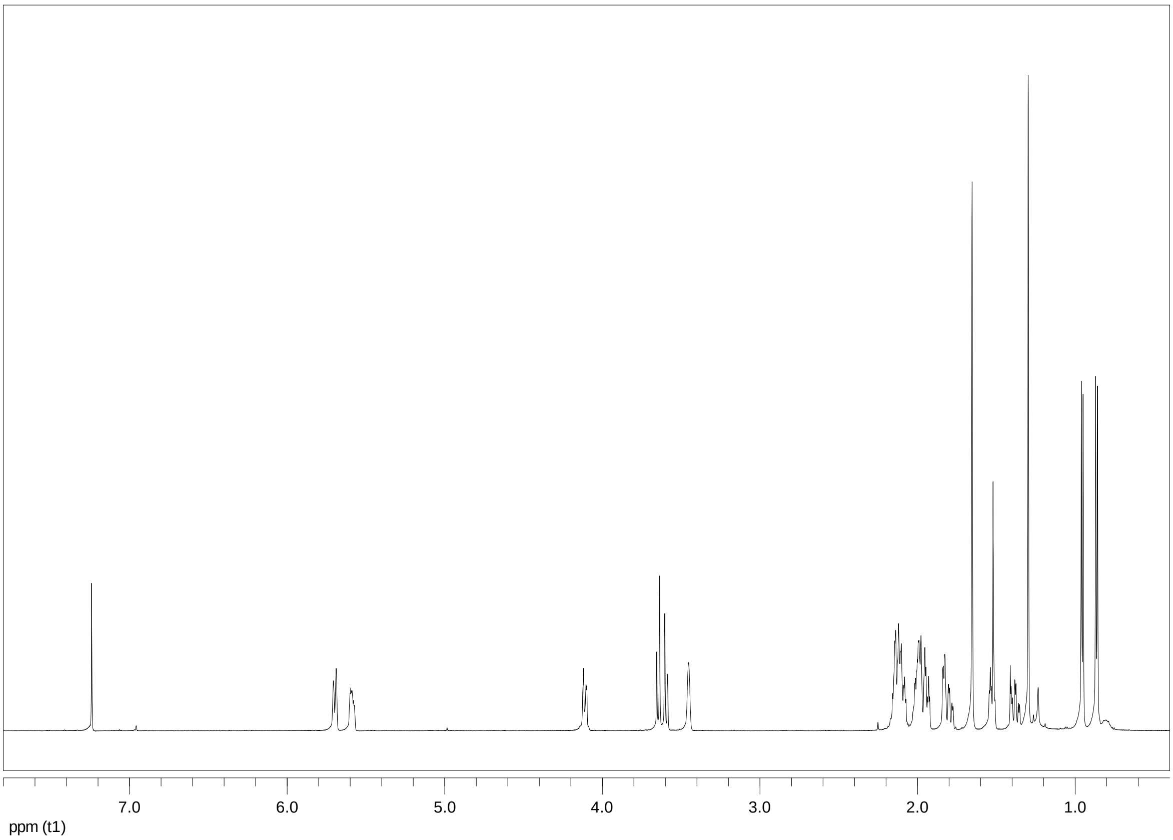

Figure S35. ^{1}H NMR spectrum ($CDCl_3$, 600 MHz) of compound **10**.

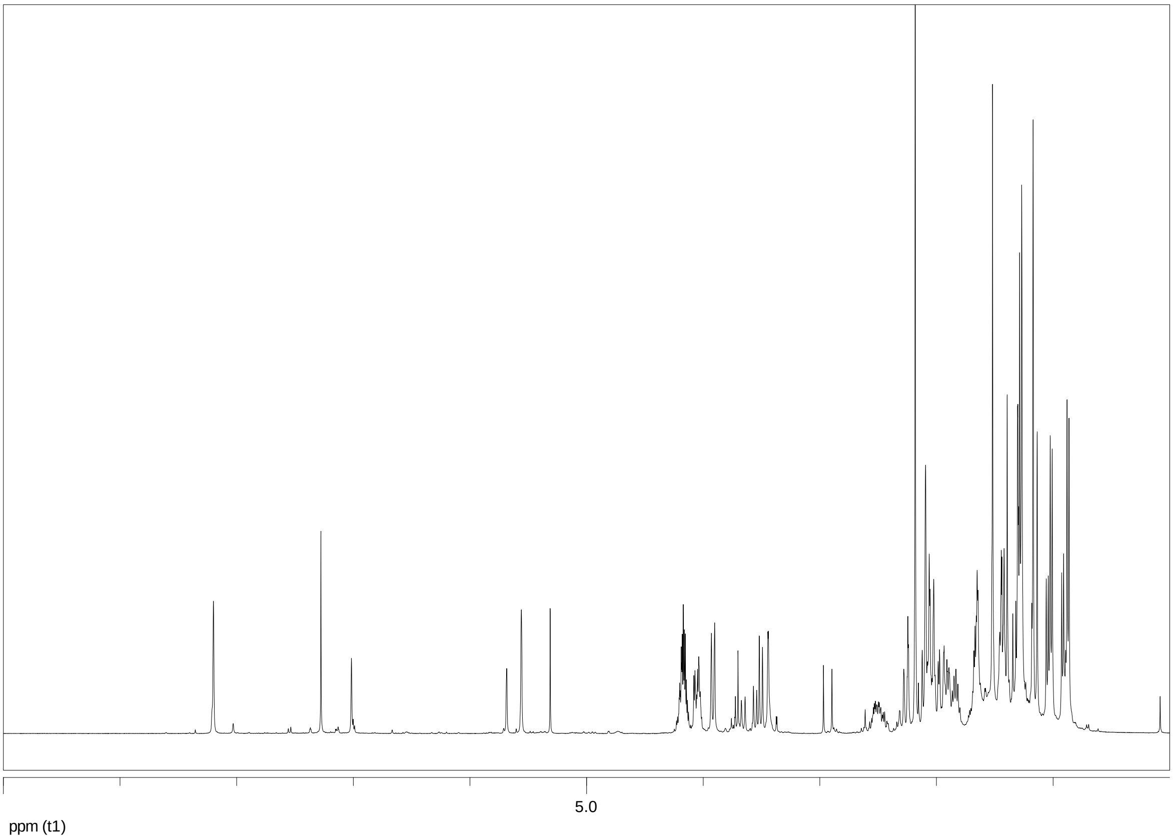

Figure S36. ^{1}H NMR spectrum ($CDCl_3$, 600 MHz) of compound **11** (mixture of *E,Z* geometrical isomers).

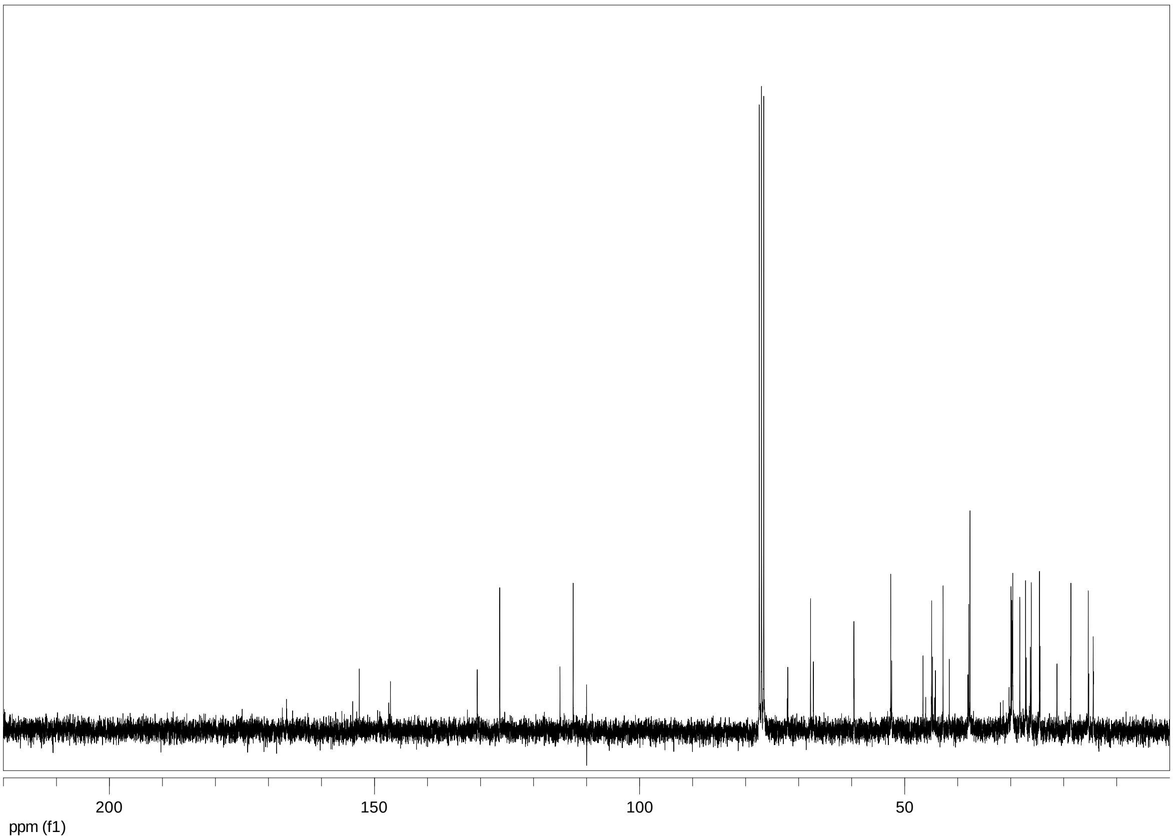

Figure S37. ^{13}C NMR spectrum ($CDCl_3$, 75 MHz) of compound **11** (mixture of *E*,*Z* geometrical isomers).

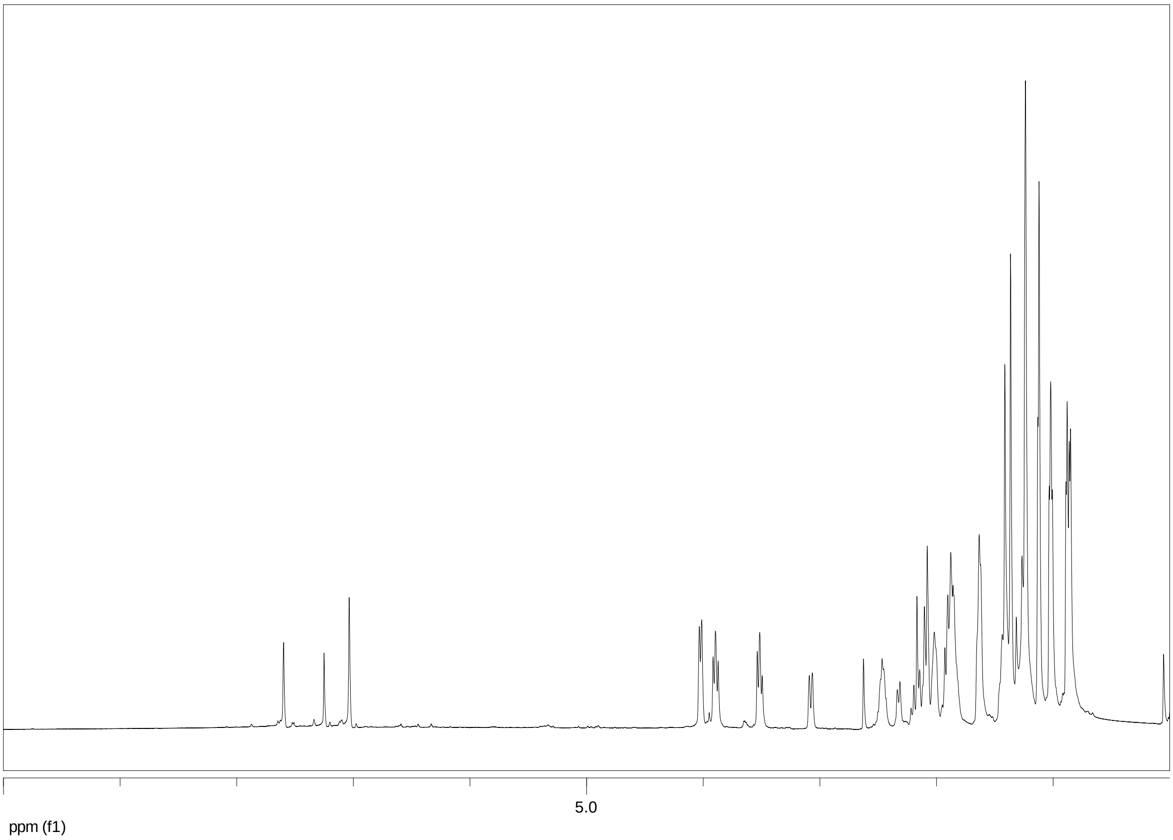

Figure S38. ^{1}H NMR spectrum ($CDCl_3$, 600 MHz) of compound **12** (mixture of *E*,*Z* geometrical isomers).

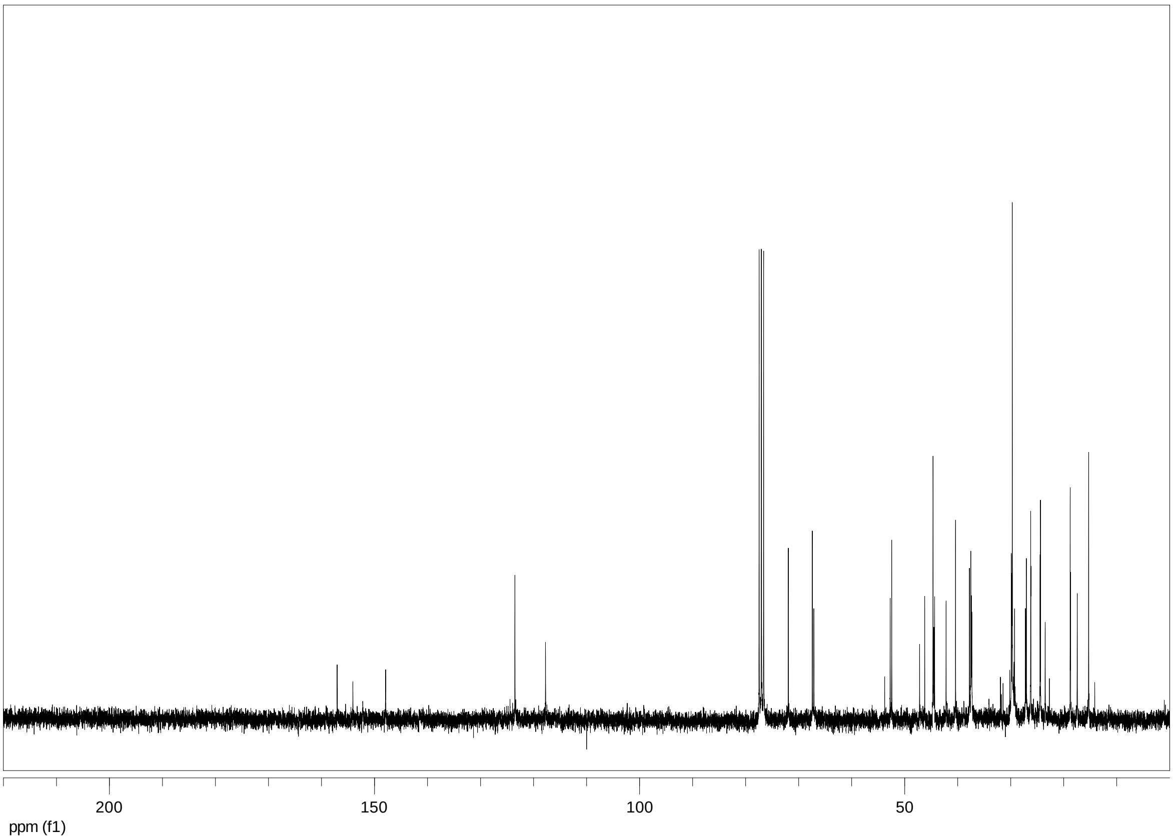

Figure S39. ^{13}C NMR spectrum ($CDCl_3$, 75 MHz) of compound **12** (mixture of *E,Z* geometrical isomers).

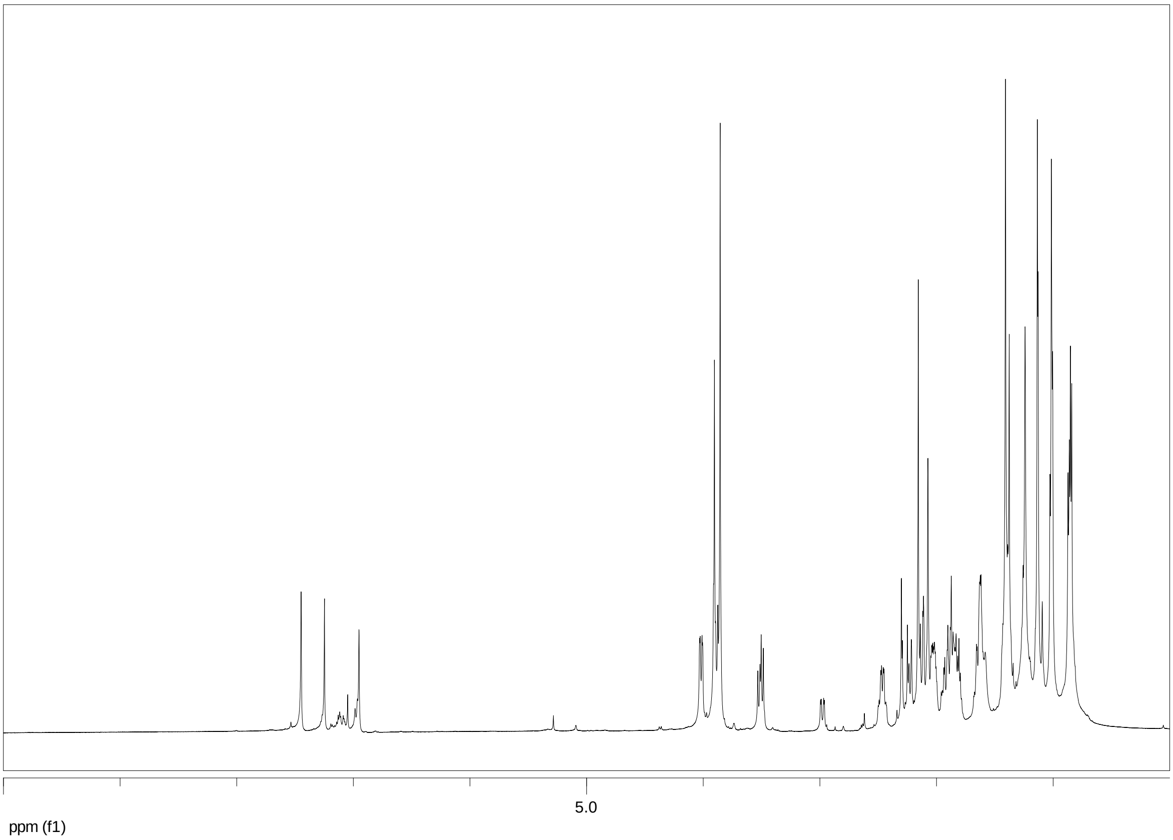

Figure S40. ^{1}H NMR spectrum ($CDCl_3$, 600 MHz) of compound **13** (mixture of *E,Z* geometrical isomers).

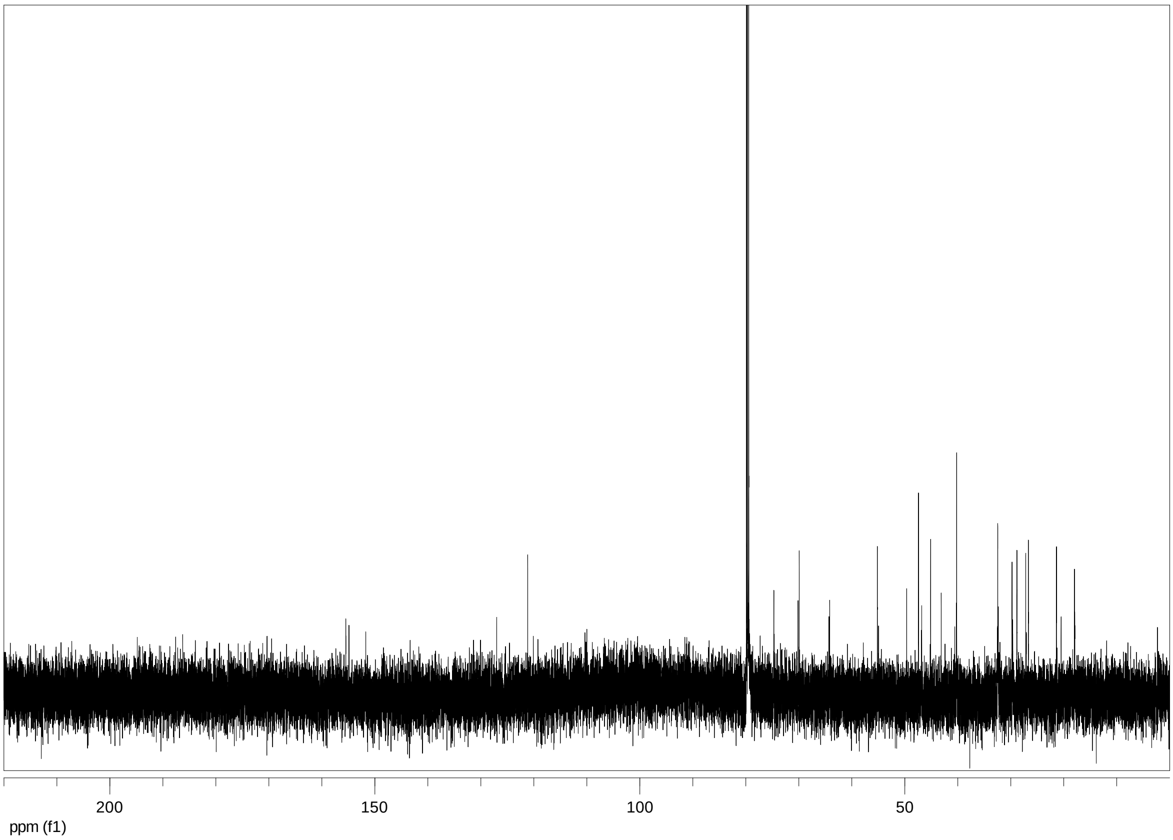

Figure S41. ^{13}C NMR spectrum ($CDCl_3$, 75 MHz) of compound **13** (mixture of *E*,*Z* geometrical isomers).

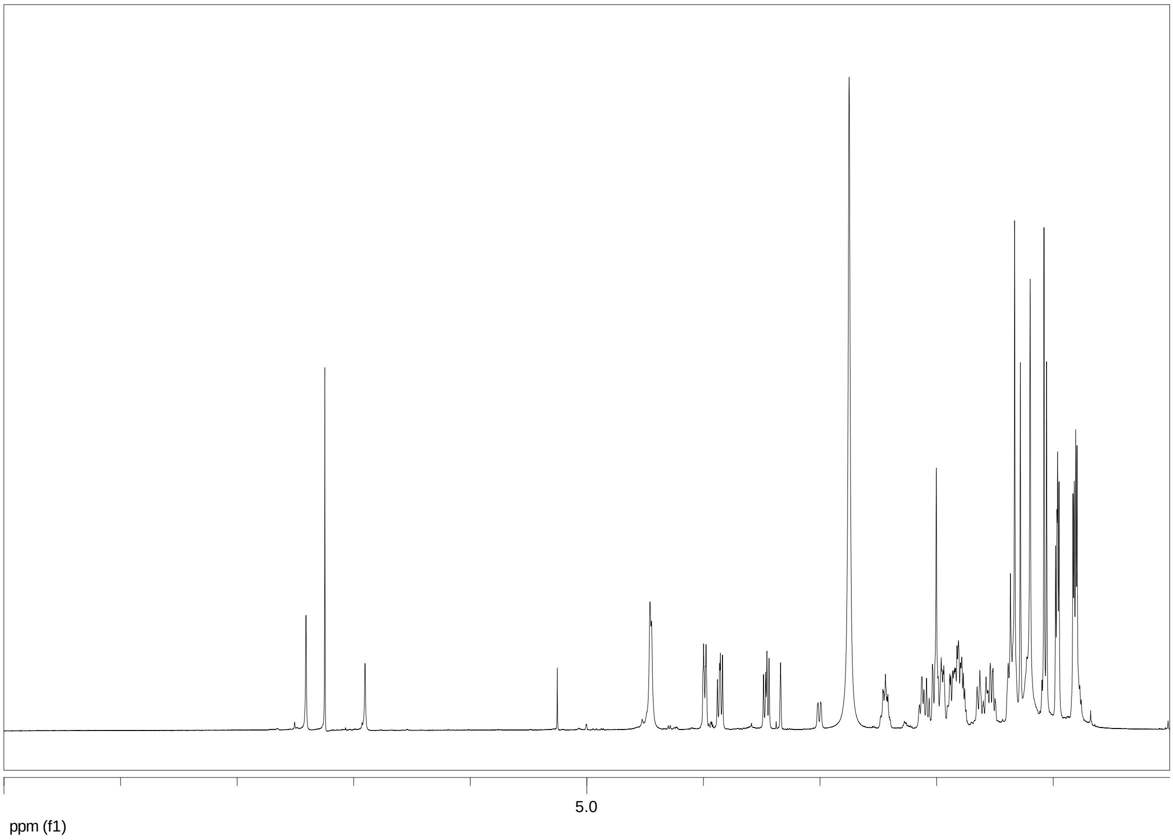

Figure S42. ^{1}H NMR spectrum ($CDCl_3$ / CD_3OD, 600 MHz) of compound **14** (mixture of *E*,*Z* geometrical isomers).

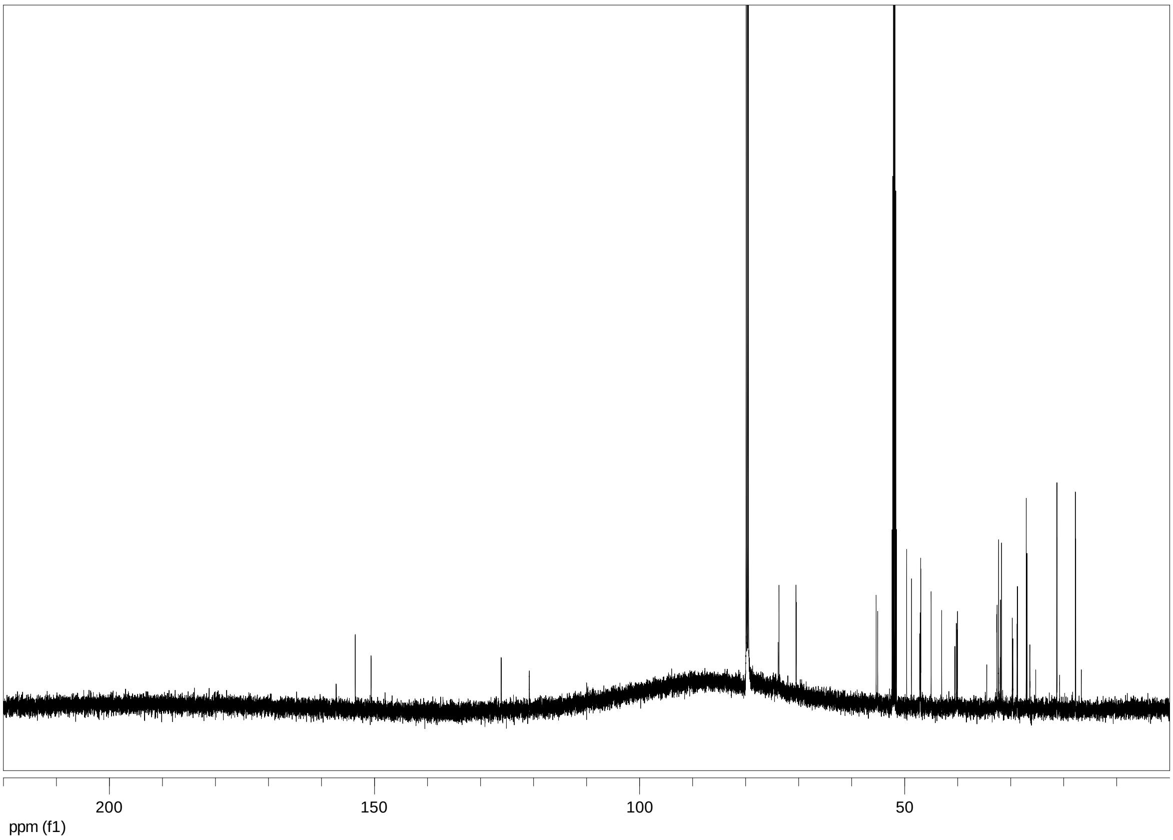

Figure S43. ^{13}C NMR spectrum ($CDCl_3$ / CD_3OD, 150 MHz) of compound **14** (mixture of *E,Z* geometrical isomers).

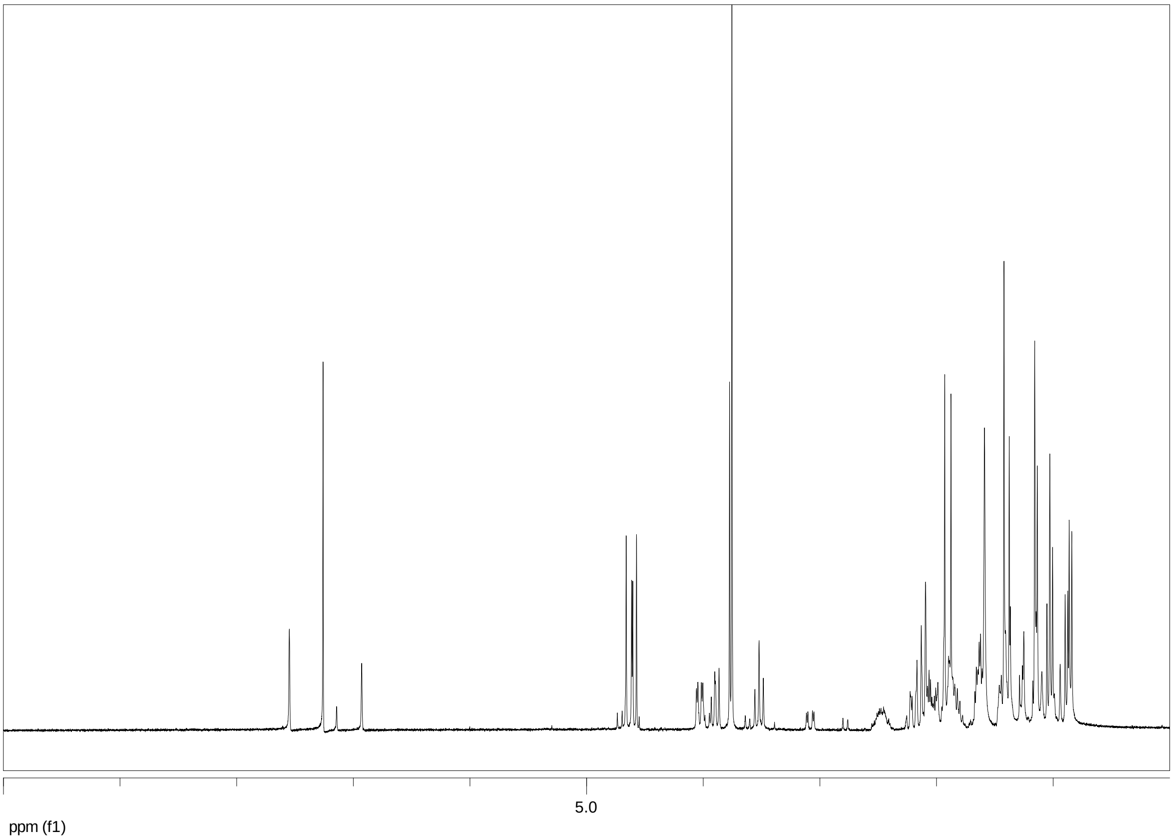

Figure S44. ^{1}H NMR spectrum ($CDCl_3$, 300 MHz) of compound **15** (mixture of *E,Z* geometrical isomers).

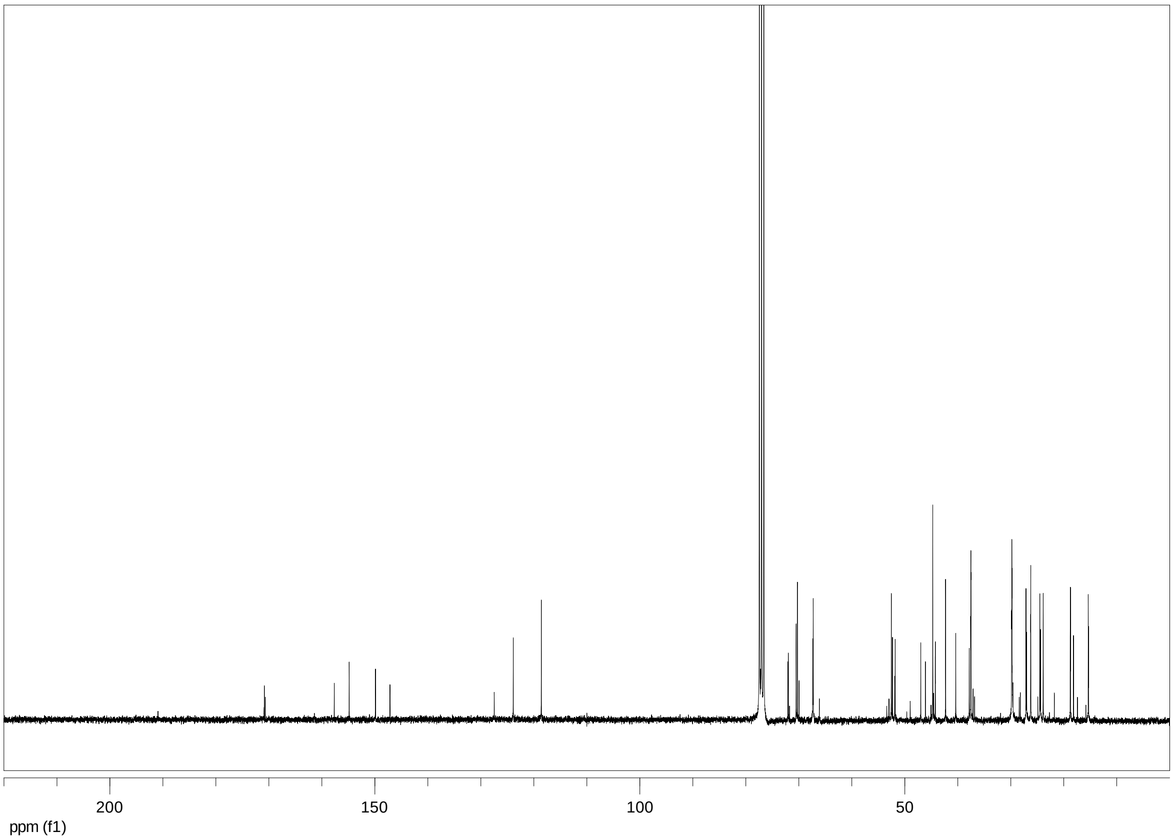

Figure S45. ^{13}C NMR spectrum ($CDCl_3$, 75 MHz) of compound **15** (mixture of *E*,*Z* geometrical isomers).

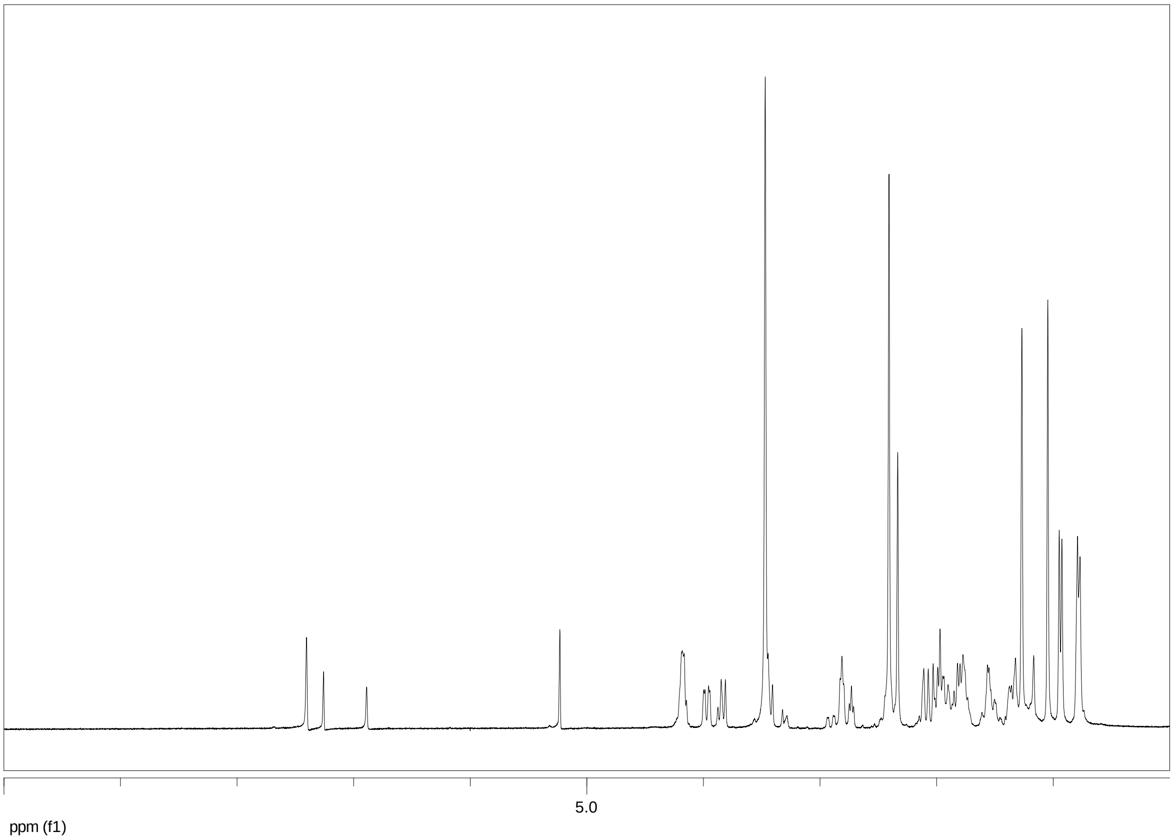

Figure S46. ^{1}H NMR spectrum ($CDCl_3$ / CD_3OD, 300 MHz) of compound **16** (mixture of *E*,*Z* geometrical isomers).

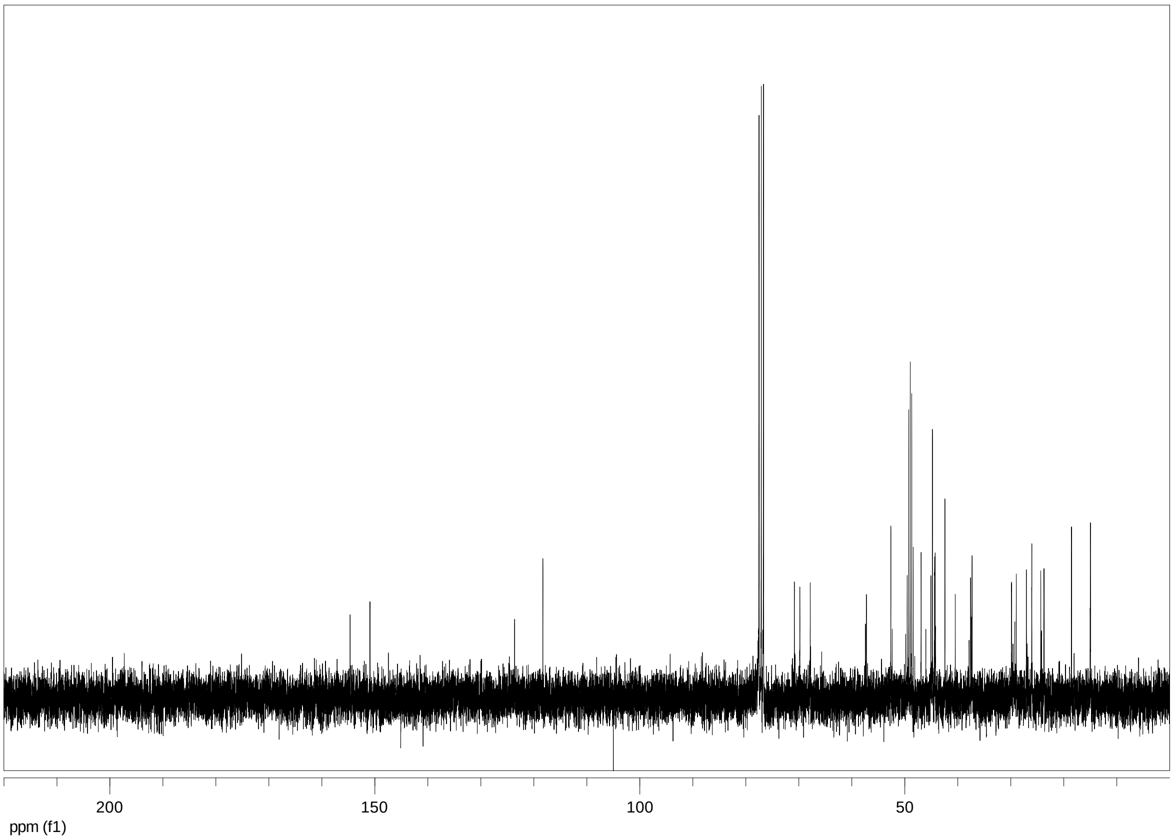

Figure S47. ^{13}C NMR spectrum ($CDCl_3$ / CD_3OD, 75 MHz) of compound **16** (mixture of *E,Z* geometrical isomers).
